# Supplementary material for: Impact of mindfulness on football coaches: A mixed design
Source: PLoS One. 2024 Oct 3;19(10):e0306824. doi: 10.1371/journal.pone.0306824 (PMC11449272; doi:10.1371/journal.pone.0306824)
Supplement: S1 File — (DOCX) [file pone.0306824.s001.docx]

**ENTREVISTA 1. ERIC (Coordinador, 30 años) – Código P.1**

**Realizada *online* el día 31 de marzo del 2021**

**E: A veure... Grabando... Perfecte. Vale Eric, si vols comença’t presentant, digues el teu nom i el càrrec al club i jo et faré preguntes a partir d’aquesta informació. Jo la faré servir per fer una avaluació qualitativa.**

P: Perfecte!

**E: Vale...**

P: Nom i cognoms?

**E: Sí.**

P: Nom i cognoms... Eric i ara mateix coordinant l’etapa pre-competició, nou equips.

1. **E: Molt bé... Com diries que el programa t’ha impactat si és que ho ha fet d’alguna manera?**

P: Jo crec que sobretot el que m’ha generat és il·lusió de... si ho hagués de resumir amb les sensacions, il·lusió de veure gent connectada... al final era una activitat que mai havia fet en grup, amb gent propera o amb gent que compartís el dia a dia i m’ha sorprès positivament que... la predisposició de la gent, l’actitud oberta de voler compartir (O.2), de voler aprendre, de voler obrir-se a una activitat nova (RI.4) i bé, a nivell personal m’ha servit tant els dies que practicàvem... els dies que practicàvem m’han servit com per establir una pausa molts dies... eh... molts dies ho hem fet al migdia, i bueno una mica per estar allà a la meitat del dia m’ha servit per baixar revolucions molts dies i... com començar el dia novament... (AG.4) fem una pausa i dient “vale, recol·loquem, posem tot a lloc i continuem” (PE.2) . I... després, al llarg de les setmanes, els dies que no practicàvem, que no practicàvem en grup, però havíem de practicar cadascú amb si mateix... mmm... crec que ha estat un reforç important a prendre consciència de la importància d’estar present... bé, de tenir aquesta actitud conscient, davant de moltes situacions (AG.1). Crec que ha estat un reforç, no? jo ja personalment estava seguint un procés personal i el fet d’haver... haver-me inserit en aquesta activitat ha estat un reforç important, perquè al final bueno, quan un porta un temps ja en etapes... bueno almenys jo, personalment, en etapes en que estic més connectat, etapes en que estic més desconnectat... a vegades certes circumstàncies exteriors poden convertir-se amb excuses per al cap d’un temps estar més desconnectat, per dir “bueno, avui... deixo de banda fer meditació o deixo de banda el que sigui”... i el que comença per un dia al final s’acaba allargant en el temps... ja no només la meditació, el prendre’s la vida d’una determinada manera... (AG.3) a vegades hi ha unes certes crisis, sobretot quan un sent que fa coses de forma incoherent, no?, que es van generant incoherències entre el que sent, el que pensa, el que fa... i quan això es va incrementant en el temps i s’allarga... un es desconnecta i puff!... En el meu cas moltes vegades sento que perdo... anava a dir... perdo el control, però perdo com una mica... em sento desorientat quan entro en aquestes etapes i... i bé, crec que haver estat en grup en aquest programa de *mindfulness,* no és que et converteixis en buda de sobte, però, t’ajuda a centrar-te més (AG.3).

**E: Molt bé, i a nivell professional, què creus que t’ha impactat més a nivell professional en el sentit de que puguis aplicar?**

P: Doncs mira, més que l’eina... ens vas comentar l’eina de... a nivell de coordinadors seguir estratègies de concentrar-te en una tasca sense inputs externs, sense deixar entrar determinades coses (PE.3). En el meu cas, més que això, mmm... m’ha ajudat i m’ha donat reforç a la hora d’interactuar en reunions d’estar amb gent de Barça Escola i d’escoltar més activament (RI.2) i actuar de forma menys impulsiva... o sigui de reflexionar millor què dir, perquè dir-ho, en quin sentit i no actuar de forma impulsiva que, fins i tot moltes vegades he actuat de forma impulsiva, en determinats moments (AG.3), però crec que... que fer aquest curs ha estat un reforç molt important per incrementar la consciència en aquests moments, a vegades hi ha moments de tensió, moments de gestió de situacions a nivell intern... emm... amb la resta d’*staff* i.... crec que m’ha ajudat a estar en un rol més d’observador (AG.1) i a l’hora de participar... d’actuar, però d’una forma molt més conscient i menys impulsiva.

1. **E: Molt bé. Llavors, bastant lligada no, perquè la següent pregunta és si has modificat o has incorporat algun comportament, o alguna estratègia o algun aprenentatge en aquesta... en aquest rol laboral... com resumiries el que acabes de dir?**

P: Bé... en relació al que hem comentat al principi de l’estratègia de fer... em sembla de 45 minuts, eh... de... concentració en una tasca (PE.3), 15 minuts de descans, de recuperació emm... perquè m’ha ajudat també en aquest sentit a prendre més consciència de a vegades simplificar (AG.3), de dir “vale, què és el més important? Vale, això és el més important i em centro amb això”. Ser més minimalista en aquest sentit i... prioritzar moltes vegades les coses importants i... i abordar a vegades aquests *inputs* externs, però a vegades no en aquell moment i dir “vale, m’arriba això, això ho faré en aquest moment però ara estic centrat en això que és el més important (AG.1)” i... i ja està. Bueno també crec que vam tenir una parada al Nadal per vacances i... en tota la dinàmica que portem de la pandèmia, etc., jo crec que tots estem una mica més conscients que abans de la importància que té cada dia perquè bueno, potser un dia anem a entrenar i el dia següent ja no tornem... A mi em va passar a Canadà que, que vaig anar un dia a la oficina pensant que el dia següent tornaria i mai més vaig tornar a la oficina i... i crec que també participar en aquest curs m’ajuda a connectar a l’hora de dir “ostres!, les interaccions amb els entrenadors són importants o poden ser transcendents en el dia a dia”. No podem deixar per endavant el que avui ja podem fer i totes aquestes connexions que a nivell personal es poden donar s’han de viure avui ... és una mica la filosofia que des que vam començar aquest 2021 vaig, vaig començar entrenaments i vaig pensar ostres!... no sabem quant temps estarem entrenant, no sabem quant temps estaré com a coordinador jo per exemple i vull aprofitar cada dia (AG.3) aquesta interacció amb entrenadors i no convertir-ho una mica en el dia de demà i dir, “vale un dia més aquí, un altre entrenament,...”; no convertir-ho en una cosa rutinària sinó que pugui tenir una transcendència, no de la meva part cap als entrenadors sinó a la relació entre nosaltres (RI.1).

**E: Molt bé... i a nivell personal Eric, has modificat o has incorporat algun comportament o alguna estratègia o algun aprenentatge?**

P: Si... sobretot m’ha ajudat a prendre consciència de determinades coses i, per exemple, el tema de la... jo sóc força quisquillós... bueno quisquillós no seria la paraula, diria emm... cuidadós amb el tema dels àpats per exemple...bueno, m’agrada força menjar bé, menjar en condicions, no estar davant d’una pantalla dinant (PE.2.2). Vaig estar a Canadà durant un any i mig dinant al *Dome*, com la bombolla on entrenàvem, amb els professors, venien els alumnes per allà mentre dinàvem... era una mica a vegades incòmode... no sentia que tenia una intimitat per dinar, portant un taper de casa allà al... a l’escola i ara tinc la oportunitat de dinar a casa meva i... i bé, ho valoro molt. També ho vaig començar a valorar en temps de confinament, el poder fer les coses més tranquil·lament i més naturalment diria, no?

**E (So d’assentiment)**

P: Com seria potser natural i... bé com entrenem per les tardes moltes vegades dino i surto corrent cap a... surto... acabo i marxo cap a la feina i... bé, m’ha ajudat també a aquest procés fer-lo... a dies eh, perquè hi ha hagut dies que no ho he aconseguit, però almenys he estat conscient del que vull i ara he d’entrenar en coherència amb el que vull que és poder fer-ho tranquil·lament, sense presses (PE.4)... una cosa que em senta molt bé és acabar de dinar i tenir un temps de repòs, de no ficar-me l’últim tros de menjar a la boca i dir, ja estic vaig a fer una altra cosa...

**E (So d’assentiment)**

P: Doncs, tenir una mica aquesta pausa emm... m’ha ajudat a ser conscient en aquesta activitat. Hem fet com diverses activitats així pràctiques, com la de dutxar-se conscientment, conduir conscientment (PE.2.2)... tema dutxar-me em costa, perquè tinc l’hàbit de començar el dia amb moltes idees i moltes vegades estic allà i em fico a imaginar, imaginar, imaginar... també començo amb una mica d’aigua freda...

**E (riu): Sempre va bé...**

P: Que em treu tot del cap i...

**E: Digues si volies dir alguna cosa més...**

P: No, ja està.

1. **E: Què creus que és, si haguessis de destacar el més beneficiós per tu... del programa?**

P: Doncs, m’ha ajudat a... en el meu cas seria a aprofundir en la relació amb algunes persones (RI.4) com tu, com el Cesc, el Andreu per exemple, vam tenir l’últim dia la xerrada aquesta que estava programada per 6 minuts i ens va durar al final 20 minuts. Van sortir temes personals força interessants...

**E (So d’assentiment)**

P: I vam sentir que havíem d’aprofundir i... en el meu cas, crec que veure a la gent en una altra tessitura diferent a vegades més allunyats de formalitats diàries i... i més allunyats de... no, al final jo crec que quan estem a la Ciutat Esportiva, i més aquesta any que no tenim espai de vestidors, que no ens en anem a esmorzar junts, que no hem fet cap sopar d’entrenadors... eh... doncs la gent arriba i l’hora i quart que està entrenant està concentrat en el seu equip i són moments en que un té el focus en els jugadors i és difícil allunyar-se d’això i... potser fins i tot no pertoca allunyar-se d’això i... centrar-se en les relacions i... ha generat també un espai d’intercanvi molt interessant i poc superficial, no? Jo crec que la gent el que deia ho deia amb un sentit i una intenció i no ho deia d’una forma superficial com moltes vegades ens relacionem o es relaciona la gent en determinats moments del dia. Això, m’ha alegrat molt la veritat... m’ha alegrat molt que tinguem aquest espai (O.2).

1. **E: Si haguessis de destacar algun repte o dificultat, que t’han sorgit per completar el programa, si és que n’hi ha hagut...**

P: Bé, emm... dificultat crec que va haver una setmana que no vaig estar present a la sessió i això em va desconnectar força de la dinàmica i... i bé, en el meu cas hi ha hagut dies en que no... o sigui, que he oblidat durant la setmana les activitats conscients que ens havíem planificat i... i bé, la major dificultat potser ha estat donar continuïtat a les coses que ens havíem plantejat al llarg de la setmana (PE), quan altres situacions a nivell personal, laboral... que al final és el mateix, totes són personals, totes són... o sigui totes són personals, es van donant... emm... continuar mantenint en el focus personal allò que ens hem plantejat a les sessions i mantenir-ho diàriament al llarg de la setmana.

**E: Què t’anava a dir... la sessió que jo tinc aquí apuntada, la sessió 4, que em sembla que era la de valors, la vas arribar a veure per vídeo?**

P: La sessió de valors vaig estar...

**E: Ah, la d’emocions, perdona.**

P: No, no l’he vist.

**E: Vale, vaig a anotar-ho aquí...**

1. **E: En quina mesura ha complert el programa amb les teves expectatives inicials?**

P: Bueno...estava pensant si tenia expectatives jo... tenia la imatge no? de... tenia com la imaginació, sí, expectativa però imaginava un curs on farem meditació... on la gent, bueno, pel que coneixia d’entrenadors, trobaria un espai nou, de novetat, d’entrar en contacte amb una activitat com és la meditació i que al final també seria... seria sorprenent, el que pot impactar imaginar un espai on ens relacionéssim d’una altra manera tots, que generés confiança (O.2)... emm... dels uns als altres, crec que al final, bé, almenys jo la experiència que vaig tenir amb l’Andreu em transmetia que havia ajudat molt aquest espai perquè la gent confiés a expressar-se i.... el que m’ha sorprès i no sabia... no coneixia els integrants del grup, no sabia què plantejaríeu, quines activitats plantejaríeu, els continguts al principi fins que els vas presentar, desconeixia com s’estructuraria el curs i crec que el que potser no imaginava i que ha potser ha sortit és molta informació útil (PE.3) que ara hem d’integrar o podem integrar els que hem participat... ha sortit informació de moltes temàtiques. Com tu comentaves, és un curs pensat per... per... bueno, donaria per més hores de les que hem fet i... i bé, en el meu cas personal he de pensar quines coses m’han impactat o quines coses m’han interessat més, i almenys una lligar-me-la, crec que seria interessant... de tot el que hem parlat, perquè ha sortit molta informació i almenys començar per una i integrar-la.

1. **E: No sé si és repetida o no, però per concretar, què destacaries del curs? Què valoraries com lo millor? Amb què et quedes?**

P: Bé, crec que... que a nivell de gestió humana, en el sentit de... tu has moderat els, les trobades però també s’ha donat veu als integrants del grup, s’ha donat veu a les persones que han participat i crec que en general aquest equilibri o la forma en que tothom ha participat ha estat molt fluida, ha estat molt natural...ha tingut un punt d’estructuració però també ha tingut un punt de fluir no? (EO.1) Sempre has recordat tu durant el curs que si algú no volia parlar durant les 6 setmanes no calia que parlés, però al mateix temps encoratjaves a la gent a a expressar-se, a que no els jutjarien... i jo en el meu cas el que més destacaria és que s’ha generat un espai molt idoni i que almenys jo personalment em sentia molt còmode a l’hora de compartir i que crec que era una cosa comuna (R1.4). Sí que a vegades pel límit de temps... pel límit de temps que... de les sessions... d’una hora i quart, emm... a vegades trobava a faltar com que poguéssim parar una mica més el temps i intercanviar sobre el que... sobre la informació que s’estava donant (EO.4 + EO.2). Perquè a vegades saltàvem d’un punt a un altre punt, d’una diapositiva a una altra diapositiva de temàtiques diferents, no? I... i potser els dies que hi ha hagut més informació, són dies que hi ha hagut menys intercanvis entre els entrenadors. Però bé, clar, quan es dona molta informació surten moltes idees molt interessants i molt rellevants i el que em plantejo, jo no sé com fer-ho... però és com amb tota aquesta informació es poden donar eines per integrar-la a nivell personal... perquè un al final acabi encarnant tota aquesta informació en el seu dia a dia, en la seva forma de viure i també heu plantejat activitats durant les formacions, hem fet algunes activitats no només fora de les trobades, sinó durant que crec que també han estat interessants i han ajudat en aquest sentit (EO.2).

1. **E: Molt bé. Com valores aquest tipus de programa pel teu rol professional específic?**

P: Doncs el meu rol professional, molt positivament, jo ara estic de coordinador però crec que si estigués d’entrenador igualment seria el mateix.... emm... tot el que siguin activitats que considero a dia d’avui, en un futur potser ja no ho serà tant però ...a dia d’avui molt transgressora, en una dinàmica com la tenim. I a més, transgressora però a l’hora molt alineada amb el que prediquem, o sigui... quan parlem a nivell pedagògic com ens volem apropar als jugadors, quina és la proposta de Barça Escola... crec que conèixer el que és el *mindfulness* o conèixer el que és la meditació o viure meditativament té molt de sentit i al mateix temps és molt transgressor perquè vivim molt condicionats per la societat i pel paradigma mental en el que gairebé tots vivim... dual, això està bé, això esta malament, de jutjar, de ser molt mentals, poc sensitius... donar poc espai a vegades a les emocions i als sentiments i portar-ho tot aquí (senyala el cap) (O.2). I bé, ens ha obert un món nou (CT.1) i bueno, el meu rol, la veritat, jo... treballo, obro durant una sèrie d’hores a la setmana i al final tenir també activitats com aquesta per mi resulta trencadora amb la rutina diària...per mi és com una mica una vitamina també de fer una cosa motivant... (AG.4) el que m’agradaria seria a cada instant a la feina estar fent coses que em motivessin a l’instant, que no fos una excepció i el que al fer-ho una vegada a la setmana també m’agrada.... potser si ho haguéssim fet cada dia se m’hagués convertit més en una activitat més monòtona, però el fet de trobar-nos els dilluns, tots... emm... també fer-ho al principi de la setmana m’ha agradat (EO.1).

1. **E: Súper! Ja la última... quines millores implementaries en el programa?**

P: Ha estat molt bé... m’ha agradat molt les últimes sessions, el generar grups, subgrups (O.1), potenciar interacció entre els membres del grup (EO.2) i... el que et comentava de la informació que s’ha donat, que s’ha donat molta informació, potser reservar espais dins de les pròpies sessions, més que a fora perquè al final... jo almenys, a mi m’agrada quan m’apunto a un curs, és una mica com a l’escola, no?, que entre que els nens s’emportin els deures a casa o els facin allà a classe, jo prefereixo que els facin a classe. I que després si hi ha alguna cosa que poden mantenir en el temps, que la mantinguin però sobretot que... que s’aprofiti aquest espai per integrar. El tema del temps és una dificultat, però jo potser reduiria informació, potser alguna informació no l’hauria donat i me l’hauria guardat per futurs cursos i... fer activitats que generin interacció entre els entrenadors i, bueno... els entrenadors i els empleats del club que han participat i... i que serveixin per integrar els continguts que s’estaven compartint. Ja s’ha fet, però potser ampliaria, ampliaria això durant les trobades (EO.2).

1. **E: Perfecte. I t’agradaria afegir algun comentari més final?**

P: Bueno, eh, gràcies, ja us hi vaig dir l’últim dia. M’ha agradat molt el que es transmetia entre vosaltres (O.1), entre els membres del grup, entre tu, el Cesc, el P, el Marc... no sé si hem deixo algú...

**E: Bueno, estaven el Xavi i el Sergio, però han assistit menys.**

P: Sí, que no han estat en les nostres trucades....

**E: No.**

P: ...però bueno, sí que el que es transmetia entre vosaltres era la sensació de que estàveu treballant realment en equip i... bé, m’ha agradat molt, de veritat (O.1).

**E: Vale, perfecte Eric. Paro la gravació.**

**ENTREVISTA 2. DAVID (Director Academia Internacional, 29 años) – Código P.2**

**Realizada online el día 31 de marzo de 2021**

**E. La idea és que et presentis, diguis el teu nom i el càrrec que tens... no em sents ara?**

P. Bueno, he escoltat lo del càrrec que tens només...

**E. Bueno, no, que diguis el teu nom i càrrec que tens i et començo jo a fer preguntes i** **contestes el que creguis.**

P. Vale, em dic David, sóc director del projecte local del FC Barcelona a Jordània.

1. **E. Perfecte. P, com diries que el programa t’ha impactat, si és que ho ha fet d’alguna manera?**

P. Eh... bueno, sens dubte sí que m’ha impactat eh... a més a més, jo també estic vivint un context que és difícil i ha sigut un moment quasi perfecte, que estava amb el cap que em volava i a més a més m’ha ajudat molt a nivell de tècniques (AG.5), no? A experimentar noves maneres de meditar (PE.1) ja que vaig iniciar la meditació quan era molt.... tenia... bueno, farà 10 anys ja, tenia 20 anys, i no me’n vaig sortir. No sé, potser no estava en un moment vital que no... que no hi vaig sentir i vaig tornar a iniciar farà uns 2-3 mesos... i ho vaig iniciar amb Borja Vilaseca, que em sembla que t’ho vaig comentar...

**E. Sí....**

P. ...però de forma molt introductòria... ell ens va ajudar amb l’escàner, a mi em va ajudar moltíssim... bueno lo que et vaig dir, conèixer noves tècniques, algunes tècniques em costa més, per exemple a mi la respiració pum, me’n vaig... o sigui, no connecto. En canvi, l’escàner i altres maneres m’ajuden més (PE.1)... Tot i que també els hàbits que ens has inculcat amb aquesta formació cada cop em costa menys fer aquesta meditació...

**E. Aha...**

P. ...tot i fer-la amb Borja Vilaseca, que em va ajudar molt, era molt irregular... i ara amb tu he agafat un hàbit i em posa a qualsevol moment del dia, sigui quin sigui i bueno... no em costa tant, he agafat un hàbit molt positiu (AG.1+AG.4). Per tant, m’ha impactat molt positivament (O.1), m’està ajudant molt per trobar un balanç, un balanç personal (AG.4), també professionalment a l’hora... ara que comparteixo amb els meus entrenadors sóc més conscient del que dic (RI.2), no? a vegades, posava aquest pilot automàtic (PE.4). Et dic més experiències o hi ha més preguntes?

**E. No, ves dient si vols...**

P. Vale... m’he adonat de coses quotidianes que no li donava importància, com per exemple rentar-se les dents (PE.2). El meu cap actua d’una manera cartesiana, o sigui jo em rento les dents i començo a analitzar tot el dia... ni me n’havia adonat o sigui ha sigut, ostres! Jo normalment al matí m’aixeco, a les 11 reunió, a les 12 no sé què... i amb el pilot automàtic... i quan arribo a la nit una mica el mateix, però com repassant, una espècie de repàs... una cosa que he dit, quina passada, ni me n’havia adonat (PE.4). El tema menjar, crec que mai havia sigut conscient ni dels sabors, simplement menjava, posava telèfon, la tele o un vídeo a *YouTube* i pilot automàtic (PE.2), literalment... Per tant, coses molt simples que marquen molt la diferència crec, no? No estic aquí.. sóc P però no estic aquí.

**E (Riu).**

P.. I després a partir de vídeos... per sort o per desgràcia he tingut temps ara i llavors m’he mirat tots els vídeos, *Netflix*, estic llegint bastant, estic aprofitant per llegir...i bueno, m’ha ajudat moltíssim... (O.1)

**E. Molt bé...**

1. **E. Bé, la segona pregunta...has anat dient coses, però anirem concretant. Has modificat o incorporat algun comportament, estratègia o aprenentatge a nivell laboral primer?**

P. A nivell laboral, ha coincidit que justament quan vam iniciar, a la segona tercera setmana ens van parar i per tant no he tingut massa... però les primeres dos setmanes sí que estava molt conscient als entrenaments, que a vegades estàs allà pensant i pum!, te’n vas a endemà... i començar a veure que despertava dels nanos, que a vegades quan vas amb la boragine, i que ho fas molt al principi i quan ets entrenador, no? Però havia arribat un moment, amb tanta boragine que arribava als entrenos i més o menys tot molt controlat, més o menys a nivell conceptual sabíem lo que volíem... però jo arribava i començava a veure la SSP (aclariment: exercici d’entrenament), i al començar a veure la SSP, el comportament d’aquest nano, el que buscàvem o el que fos... i ja me n’anava, literalment, als 5-10 minuts ja estava pensant, ostres, he de parlar amb aquest pare o he d’organitzar-me demà per preparar les sessions de la pròxima setmana i me n’anava... i a partir de no sé quina formació va ser, doncs vaig començar a estar més conscient i dir “ei, avui aquesta SSP sencera i observo aquest comportaments d’aquests nanos” i a més a més, que emergeix d’aquests nanos... i sortia amb molta més riquesa... i en la següent formació amb els entrenadors me’n recordo... després ja ens van tancar, doncs vaig extreure moltes coses d’uns certs comportaments que ens van ajudar i ens van enriquir una formació, per tant em va ajudar molt... a la SSP, a estar (PE.3). I també al parlar amb els entrenadors, la comunicació últimament era bastant com en pilot automàtic, almenys per part meva... era, bueno, feia formació, més o menys, òbviament me l’havia preparat, la deixava anar, girava el cap, mirava als ulls... però simplement la deixava anar, no era conscient del que sortia de la meva boca, a vegades em quedava “eh....” i ara vaig ser més conscient, escoltar millor... a vegades anava predeterminat... (RI.2)

**E. Ja...**

P. I bueno...

**E. I a nivell personal? Digues, digues, perdona...**

P. No, no, dons això, que he extret a nivell professional això i a nivell personal... bueno, primer ser conscient en moments quotidians com rentar-se les dents, com dutxar-se... (PE.2) agafar un hàbit de meditar... he provat una mica, vaig iniciar sempre... abans sempre ho feia ara a les 12, m’aixecava, treballava de 9 a 11, amb energia i a les 12 feia una meditació. Tornava a agafar energia i me n’anava als camps. Això em va ajudar... i ara he iniciat pels matins i també m’està ajudant bastant, o sigui he agafat un hàbit de meditar... i també he agafat l’hàbit de llegir cada nit, que abans ho feia més esporàdicament o agafava el meu dia lliure i me n’anava a un *coffee shop* i aprofitava 2-3 hores per llegir... i ara estic invertint cada nit fins que pugui... fins que senti que estic concentrat. O sigui, estic agafant hàbits de vida que em fan sentir més viu, més conscient podríem dir que abans (AG.4)... bueno, com quasi tothom crec, no?

**E. Aha...**

P. Amb pilot automàtic, o sigui tiràvem i veiem la *Champions* i arribàvem a la d’això, em quedava adormit amb la radio escoltant RAC1 o el que fos i el dia següent cansat... i ara almenys he agafat... fer esport, que ho havia abandonat una miqueta...petits hàbits que em fan sentir més viu, amb més vitalitat (AG.4).

1. **E. Perfecte. Si t’haguessis de quedar amb lo que ha sigut més beneficiós per tu?**

P. Osti, bona pregunta... eh... bueno, experimentar, no? Diferents maneres de descobrir-te a tu mateix (PE.4) que fins ara doncs, més o menys... *mindfulness*, però només era una manera...i ara he provat el ioga, però no m’hi he trobat bé... però almenys ho he experimentat, he experimentat les diferents maneres que ens vas ajudar a provar... i jo dins meu m’he sentit més còmodes amb algunes i altres no, cosa que ara m’ha facilitat, no? A partir d’agafar un hàbit m’ha facilitat a que ara que m’ajuda més i faig la meditació i no és com abans que hi havia dies que no... ara és, em fico i no se perquè em ve... però ja estic...

**E. Estàs connectat no?**

P. Exacte... és difícil dir-te una sola cosa específica, perquè no t’ho sabria dir, Aleix, però sí que un general... Doncs que he agafat uns hàbits de vida molt més saludables per mi, més vitals de... intento també alimentar-me millor, és que és com un tot... no? (AG.4)

**E. La roda, si...**

P. És com algo holístic, no és molt cartesià “mira, ha sigut això”, és tot una miqueta...

1. **E. Quins han sigut alguns dels reptes o dificultats, si és que n’hi ha hagut, per completar el programa?**

P. Eh... reptes, he tingut reptes i hi havia dies que “ostres!”, amb entrenadors, per la situació actual...

**E. No, però en el programa en concret, és a dir, l’entrenament o si se’t feia llarga la sessió, no si tenies algun repte o ha anat fluït... si ha anat fluït, ha anat fluït.**

P. Sí, és que ha anat fluït... alguna setmana no he dut a terme alguna part de l’entrenament, però en general ha fluït i m’ha ajudat... (O.1)

**E. Perfecte...**

P. Per exemple, jo vaig iniciar-lo i vaig dir “no, no puc” i vaig preferir seguir amb la meditació, així que bueno en general molt fluït.

1. **E. Super... Expectatives, en quina mesura ha coincidit la formació amb les teves expectatives inicials?**

P. Ostres, les ha superat (O.1). Jo pensava que seria...bueno la veritat és que no em pensava... no tenia una gran expectativa... ha coincidit amb el meu moment de vida, que fa un mes o dos que estic iniciant la meditació, que estic sent una mica irregular amb la meditació i m’està costant... “doncs som-hi, em pot servir”. I com en la meva feina amb el futbol, compartir amb els entrenador com em pot servir. Però no li vaig ficar una gran expectativa, simplement anem de forma escèptica, anem a veure que, no? I les ha superat molt, però de molt... (O.1)

**E. Aha...**

P. És mes crec, en quant a la nostra pedagogia es basa en una autonomia de l’ésser humà, que com més autònom ets, més comparteixes, és fonamental tot procés pedagògic quelcom així... perquè a mi mai m’han ensenyat així... (CT.4) ho vaig descobrir per Borja Vilaseca perquè em va arribar i vaig dir “ostres, això m’agrada, m’ha donat una carícia, m’ha pessigat al cor!” I així poc a poc pues...

1. **E. Bua, pos m’alegro molt d’escoltar tot això... i què, què destacaries del curs... dius que les ha superat... què destacaries del curs?**

P. Et dona més eines per ser més eficient (AG.5) en algo tant complex, perquè al final és... no sé què opines, però és un metafísic, no és algo certer... i clar, a vegades t’ho deixen anar i et ressona, et connecta, t’acaricia i et quedes, vale, què abstracte...

**E. Aquí has pogut aterrar una mica.... perdona eh... no sé si has pogut aterrar una mica totes aquestes coses, no?**

P. Exacte, tot això que et ressona tant, que ho has escoltat i llegit també, però ara ets més conscient (AG.1) i sobretot les eines...moltíssimes eines (AG.5), algunes no t’hi sents tant còmode però amb altre si.. però que sense donar-me compte durant aquestes sis setmanes tinc un hàbit (AG.4)... òbviament un hàbit amb moltes pertorbacions, però tot i així segueixes estant i ets més conscient... abans venia pertorbació i el sentiment et desbordava a vegades, jo sóc molt de córrer a vegades i em desbordava... ara, anem a relativitzar tot una mica... doncs potser això, aquestes eines... (AG.3)

**E. Perfecte...**

P. ...algo tant abstracte, almenys el pots encotillar una miqueta, no?

1. **E. Sí, no, no, està bé... llavors, com valores aquest tipus de programa pel teu rol** **professional?**

P. Fonamental... o sigui, fonamental. Repeteixo, crec que tot procés pedagògic, al final nosaltres som pedagogs, el que volem és que tot ser humà es vegi amb un potencial seu... cadascú tindrà el seu potencial, però que es vegi, no? I clar, si el líder o algú que té un rol important en aquest... no ha tocat aquestes eines, crec que et falta algo... sí que més o menys pots tenir eines, però és que ho has d’experimentar, de viure... per després poder expressar-ho també, millorar comunicació (RI.2) també, millorar en molts aspectes... m’he explicat, Aleix? És que a vegades és tan abstracte parlar d’aquest tema que...

**E. No, està perfecte, està perfecte... Jo el que faig simplement és tiro unes preguntes, vaig recollint els conceptes que surten per després fer una mica el resum. El que passa amb el programa és que a nivell de qüestionaris, per exemple, amb els jugadors no m’han sortit millores significatives, llavors a nivell científic jo no puc demostrar que el curs hagi valgut, però jo entenc que a nivell qualitatiu això ha impactat a la gent, no? Llavors és dir, quina informació podem treure d’això que potser no es veu reflectida als qüestionaris... llavors aquí la importància no és explicar molt bé res, sinó que simplement treure pensaments i jo els ordenaré després...**

1. **... i ja et ve la última i després ja et deixaré que comentis el que vulguis, però la idea és que quines millores, si és que implementaries algo, proposaries o faries?**

P. Mmm... clar és que a vegades, jo faria que tothom... és que potser és massa, no ho he pensat massa bé, eh, és el que m’ha vingut, que tothom expressés una miqueta la seva opinió, però també pot ser que sigui massa fort, que potser la gent no ve massa receptiva, no? Però m’agradaria haver escoltat més, a moltíssima més gent... també molt constrenyits per un temps, per 1 hora... que tots haguéssim passat a expressar més coses... i a vegades a l’escoltar als meus companys és quan deia ostres, pos jo també! I estar amb aquesta por i dir jo no vull dir això, i de sobte el Johnny deia això i doncs mira... jo també estic una mica lluny i estic amb un procés, suposo com tothom, no? Però a vegades em fa cosa dir coses que semblen debilitats, no? O que jo em crec que són debilitats i no vull expressar-les per... no són ni pors eh, però pel que diran o per... simplement està allà, equilibrat (O.2) i... i jo abans d’arribar a Barça, per exemple, jo m’expressava sempre, no tenia cap dubte, sempre preguntava, no? Sempre estava al peu del canó... a les universitats, amb moltíssima gent anava i preguntava... i des de que estic a Barça m’he constrenyit molt, o sigui no sé si és el poder del Barça o... que no acabo de ser jo mateix i a vegades ho penso i, jo que sé, potser em foten fora en 2 setmanes i és algo que no em puc perdonar, saps? Però em segueix passant, que em costa compartir amb companys, per una por de què diran, lo que sigui, no?

P. Realment el que emplena es veure els nens feliços, veure com la gent és cada cop més conscient...

**E. Aha...**

P. ...es coneix més a si mateixa jugant a futbol, que s’expressa, que es veu cooperativitat... i a mi és això el que em fa feliç i a vegades ens confonem...

**E. Ja...**

P. Ens confonem pels càrrecs, pel club en el que estem, que és tant bestial... que és l’excel·lència crec jo...

**E. Ara m’ha vingut una de les preguntes que t’he fet, que era si has incorporat alguna estratègia, etc. Penses incorporar alguna estratègia per exemple a la teva acadèmia? Has pensat com pots fer el mateix amb els entrenadors o algo així? O amb els nens com pots...**

P. Jo amb els entrenadors... és la meva opinió, que potser em puc confondre... tinc 3 només, però 2 d’ells no els veig preparats encara...

**E. Ja...**

P. Per situació vital, tenen 25 anys... per ficar-se amb el *mindfulness*, més que res perquè jo he experimentat, no? Quan tenia 20 i pocs anys i....

**E. No et va ressonar...**

P. ...no em va ressonar, no? I de sobte, hi ha un moment, pues sobretot a partir de crisis emocionals o existencials, perdó i dius “eh, o canviem...”, saps? I ara és quan et comença a ressonar... i encara no els veig. El que sí que vull començar, gràcies a tu que t’ho anava a dir, compartir alguns vídeos (EO.3), no?

**E. Aha...**

P. ... pos un cop per setmana o incorporar-los a les meves formacions, no? Certs vídeos, certs talls, algunes lectures... de lectures he intentat ficar-los-hi no...

**E. Ja...**

P. Encara no els veig en el moment existencial... però no com ho has fet tu, que m’ha semblat una passada i molt valent (O.3), però sí de poc a poc anant-los deixant petjades. Gràcies a tu, incorporar elements que ens has facilitat. És el que tinc pensat.

**E. Clar...per això la idea de fer el manual, no? Per què al final al manual són moltes coses que pots anar estirant del final, no? Molts exemples i autors, hi ha història... allà és el que he fet després de 5 anys que porto en aquest procés, no? Per mi és molt valuós, probablement hi ha gent que no s’ho mirarà, però quan la gent comenci a entrar o la gent, com tu, li pot anar trobant el valor... per què una cosa és el que he enviat per *mail*, però també en el manual... perquè tampoc volia saturar, no? I la meva idea sempre és fer *pildora* i, si tu et ressona, a través de cada un dels autors en pots trobar 20 més.**

P. Totalment... em sembla que Fernández per exemple m’he vist 3 o 4.... i així he anat pescant del fil. I bueno, ara mirant-me el manual doncs, per exemple, he vist o m’he iniciat a *Netflix* amb el de *Doc Rivers*...

**E. Sí, està bé...**

P. ... m’he iniciat, em sembla que pot ser algo bo, pot ser interessant pels entrenadors... compartir la pròxima setmana això no? I ara que estem parats, a veure si els pot anar ressonant...

1. **E. Super... I res, la última, si vols afegir algun comentari final?**

P. Bueno, simplement gratitud, gratitud (O.1) Aleix per fer-me... bueno per facilitar-me, donar eines (AG.5) que em ressonin tant, no? Seguint pel camí, m’ha donat la importància de camí... i sobretot per, sense voler, amb el temps adquirir uns hàbits...que òbviament encara els he de fer mes forts, però he adquirit uns hàbits (AG.4) , per lo tant simplement gratitud...que tant de bo, no depèn de mi però ant de bo sigues a les escoles, tant debò tothom als que ens dediquem a la pedagogia, no? Al compartir... pos poguéssim experimentar quelcom així, que segur que tothom... bueno, no sé, estic dient aquí...

**E. Ja...**

P. Bueno, jo crec que a tothom li ha ressonat, almenys pel que he parlat amb companys. No sé si tant com jo, perquè a mi m’ha ressonat molt (PE.4), però tothom el feedback és molt positiu (O.1)... i més després de la crisi que estem vivint, de valors, d’una pandèmia... pos aquí hem arribant a moments tant baixos que quelcom així ens ajuda, no? Ens ajuda a relacionar-nos (RI.3) i sobretot a agafar eines perquè ens allunyem el mínim possible de l’ego, no? Que ens emporta...

**E. Super P... Vale, paro aquí la gravació.**

**ENTREVISTA 3. JOAN (Entrenador, 24 años) – Código P.3**

**Realizada *online* el día 31 de marzo de 2021**

**E: Simplemente dices tu nombre y tu cargo en la “Escola” Joan entrenador, y ya está. Tú mismo.**

P: Buenos días, soy Joan entrenador de la Barça Escola, del equipo Oshoala concretamente. He participado en el programa de *mindfulness* para entrenadores del club.

**E: Vale Joan, te haré una serie de preguntas, ¿vale? No hay respuesta correcta ni incorrecta, simplemente es para valorar tu experiencia y que eso nos sirva para seguir evaluando el programa. La primera es… y puedes mejor contestar en castellano así yo ya voy transcribiendo. Eh…**

1. **¿Cómo dirías que el programa te ha impactado, si es que lo ha hecho, de alguna manera, tanto a nivel profesional como a nivel personal?**

P: Bueno, yo creo que el mayor impacto que ha tenido el programa es el autoconocimiento, ¿no? y el permitirme darme cuenta de cosas que entes quizás no te dabas cuenta. Cosas que quizás antes daba por hecho que eran normales o que era así y ya está, pues con el programa me he dado cuenta de que no son tan normales como me pensaba y que, bueno pues seguramente hay una manera de cambiar este tipo de pensamientos, este tipo de creencias para poder dar mi mejor versión, tanto personalmente como profesionalmente (PE.4).

**E: Podrías dar algún ejemplo de cosas, de darte cuenta de cosas.**

P: Sí, la gestión de los nervios, la gestión de pensamientos tanto positivos como negativos (AG). Por ejemplo, en este caso, ¿no? decía que, en los momentos de los éxitos, porqué en el programa de *mindfulness* hablamos mucho de los fracasos, pero en los momentos de éxito, cuando te relajas, pues un poco esta gestión emocional y que ni lo malo es tan malo ni lo bueno tan bueno. Mantener ese control emocional (AG.3) para poder mantener la mente focalizada en la siguiente acción, en la siguiente tarea o en el momento presente (AG1.1), vaya. Y a partir de ahí pues gestionar mejor como reaccionar (AG.3) a situación del día a día (AG.4).

**E: Como sí …, bueno así para cómo responder, ¿vale? En lugar de reaccionar lo que hablamos es de responder, ¿no?, que es esta capacidad de mi respuesta, no es una reacción, no, no, es una respuesta.**

P: Analizar toda la situación, analizar todo el problema, analizar pues todas las variables que pueden influir ¿no? ¿Cómo es la otra persona? ¿Con quien me estoy encontrando? (RI.1) ¿Cómo estoy yo emocionalmente? (AG.2) Y como a esa persona o que esa persona me ayude a mi a gestionar todo eso para…, para este tipo de cosas.

**E: Super, vale. Englobo aquí nivel profesional y personal, ¿no? Por que...**

P: Sí y tanto, sí, sí.

1. **E: ¿Has modificado, incorporado algún comportamiento o alguna estrategia, o algún aprendizaje a nivel laboral, por ejemplo?**

P: A nivel laboral… bueno, trato de mantenerme un poquito más al margen, por decirlo así, para poder observar todavía más (PE.3). Eh, trato de, aunque ya lo hacía, trato de realizar todo de manera previa, de la preparación ¿no? del entrenamiento, de la preparación del partido todavía más incluso, manteniendo la mente abierta en el hecho de que, pues en nuestro caso es un deporte muy abierto y que por mucho que tu lo hayas preparado luego puede darse o no o que el jugador un día puede venir más contento o más triste, enfadado y que esa gestión es un poco ondulando, que no es, bueno que no es lineal y que no van a venir pues si ayer vinieron bien hoy no van a venir mejor que ayer porque quizá uno se ha enfadado y no… Y aunque, intentar controlarlo todo, pero entender que dentro del control vivimos en un contexto muy descontrolado. Entonces, que eso no me lleve a frustración (PE.3).

**E: (sonido de asentimiento)**

P: Y bueno, en el día a día como práctica más formal, sobre todo lo que más estoy incorporando es la práctica del yoga, porque creo que es un tipo de meditación con movimiento que me ayuda a relajarme, me ayuda a relajar tanto el cuerpo como la mente y me siento bastante cómodo con esta práctica (PE.1).

**E: ¿Y a nivel personal?**

P: A nivel personal, eh, bueno, trato sobre todo de ir más calmado (AG.3), o sea de… de analizar más todavía las cosas (AG.1), de gestionar mis emociones (AG.2). Bueno, durante el programa tuve una situación complicada y bueno, pues, mi gestión, digamos que no fue óptima, pero sí que, sí que fui consciente en todo momento de qué estaba pensando, de cómo lo estaba pensando, de… de que quizá mi manera de actuar no era la mejor, pero era consciente de ello y… regulaba un poco esa gestión, ¿sabes? (AG.5)

**E: (sonido de asentimiento) Más consciencia plena, ¿no?**

P: Sí, eso es, eso es. Más consciencia plena (AG.1) y más análisis de todo lo que engloba la situación. El permitirme un poco, no mirar el problema sino dar un paso a tras y ver todo lo que encierra en problema. (AG.3)

**E. Ver la situación en más perspectiva, ¿no?**

P: Eso es, eso es.

1. **E: Eh… ¿qué ha sido lo más beneficioso para ti, del curso?**

P: Para mí lo más beneficioso del curso es el autoconocimiento (PE.4), o sea la parte teórica ¿no? te hace ver una serie de conceptos que lo has de ir aplicando en el día a día (AG / AP). Pero toda esa parte de reflexión (CT.2) de esos conceptos, pues la aceptación, los valores, eh, los tipos de personalidad, los tipos de emociones te hacen reconocer situaciones (PE.4) que… o te hacen conocer conceptos que luego cuándo se dan esas situaciones tú te reconoces. Entonces ese autoconocimiento te ayuda más a la gestión de esas situaciones (AG), o al menos, en mi caso.

1. **E: Super. Eh.... ¿Has encontrado algún reto o dificultad para completar el curso?**

P: Bueno, eh… al final el reto o dificultad fue un poco que no tienes el hábito de hacer (AP). Entonces, sí que es cierto que a mí por ejemplo los lunes, salvo uno que me pilló de viaje y lo hice un poco de aquella manera y el otro que me coincidió con el master y tal, bueno, eh… A mí los lunes me ayudaba a decir bueno pues es este horario en este momento nos conectamos todos (EO.1) y tal. Y luego pues esto, pues, al no tener el hábito, como tu comentabas, pues yo me levanto media hora antes y medito, pues buscar esa estabilidad, esa misma hora, ese momento un poco para mí, me cuesta bastante. Soy más bien de, bueno pues, hago esto hago lo otro y más o menos me… cuando tengo el hueco lo hago.

**E: Super. Eh…**

P: La rutina, es lo que digamos que más me cuesta (AP).

1. **E: Sí, eh… crear la rutina. ¿En qué medida ha coincidido la formación con tus expectativas iniciales, si es que las tenías?**

P: Eh… Bueno, sí que es cierto que no venía con muchas expectativas, por el hecho de que no conocía muy bien lo que era el *mindfulness* porque al final, sí, pues todo el mundo hemos oído hablar del *mindfulness*, pero no sabía bien, bien a que se refería. Pero en cuanto a lo que sí que me he llevado es que veo que es algo muy muy útil para tu mundo (PE.5), que creo que ese autocontrol (AG.3), ese autoconocimiento (PE.4) es muy aplicable y nos puede ayudar mucho a cada uno, por lo menos a m (PE.5)í. Y, por tanto, el nivel de satisfacción es muy alto (O.1). Porque sí que le veo una aplicabilidad tanto a nivel profesional como a nivel personal, como dentro de lo personal en la gestión de uno mismo (AG) como la gestión de las relaciones con los demás (RI.1).

1. **E: … la gestión. Eh… ¿Qué destacarías del curso? No sé si es muy repetitivo pero bueno si destacarías alguna cosa que no hayas comentado, que quieras…**

P: Bueno, a ver. Sobre todo, eso pues para mí es muy importante y luego también es cierto que el compartir las vivencias de los compañeros (RI.4), no, lo puse un poco en el *feedback*, si que es cierto que quita tiempo del contenido teórico, que al final yo sí que creo que se queda un poquito cojo en el hecho de los lunes porque sí que es cierto que luego hay un montón de material complementario que a partir de ahí cada uno puede abrir la (ininteligible). Pero sí que es cierto que las vivencias que han tenido los compañeros y como este *mindfulness* les ha ayudado a gestionar el… a gestionar sus situaciones, hace que te sientas identificado en según qué momentos y te ayude a comprender cosas que a ti te han pasado, yo creo (O.2). Por ejemplo, en el tema de Cisco, que el comentó su tema de salud, yo me vi identificado en algo que a mí también me pasa, ¿sabes?

1. **E: (sonido de asentimiento) Sí, total. Y… ¿cómo valoras este tipo de programa para tu rol profesional?**

P: Yo lo valoro muy positivamente (O.1). Lo valoro muy positivamente porque eso al final, es que creo que tiene mucha aplicabilidad (AP / AG). Creo que, al final, como entrenadores (PE.3) estamos gestionando personas (RI.1), ya sea los jugadores, gestionamos momentos de mucha emoción (AG.2), de mucha tensión porque al final eh parece que todos los partidos, sean la categoría que sean te estas jugando la final de la Champions.

**E: Ya.**

P: Porque al final cuando tu vas en el minuto ochenta ganando 1-0 y el otro, el rival te aprieta, pues es una gestión de emociones brutal. Cuando el árbitro te pita una falta que no es, es una gestión de emociones brutal. Cuando un jugador que tres partidos no juega es una gestión de emociones. Cuando el fisio o el preparador físico piensan una cosa y el entrenador piensa otra pues igual, gestión de personas (RI.1) y emociones (AG.2). Entonces creo que ese autoconocimiento, que ese aprender a escuchar (RI.2), que ese valorar la opinión de todo el mundo y que ese intentar que todo el mundo esté a gusto o entender a todo el mundo (RI.1), siempre y cuando en el futbol pues tenemos el problema ¿no? de que el que juega va a estar más contento que el que no juega, pues intentar, como comentaron en el *Barça Coach Program* (aclaración: programa de formación interna del club), Sergi, Óscar y demás, que estén contentos en base a tus acciones y no solo al minutaje, ¿sabes?, que se sientan importantes por como tus les tratas y poder darle a todo el mundo un poco lo que necesita. Esa gestión de…, tanto personal, de las emociones, como para que esa gestión luego se traslade al colectivo (RI.1).

1. **E: Super. Eh ¿Qué mejoras implementarías en el programa, si, si lo harías?**

P: No, bueno, como mucho implementaría un poquito más de marco teórico, pero a mí también me gusta entender las cosas y comprender bien, bien todo lo que se hace y demás. Pero, al final, lo que te digo, si luego indagas por tu cuenta con el material complementario, pues allí lo tienes.

1. **E: Vale. Y quizás o del tiempo que comentabas ¿no? ¿O qué? O sea, más… bueno no, lo dejamos ahí. Vale, bueno, algún comentario final Joan, ¿algo qué quieras añadir?**

P: No, bueno, agradecerte tu trabajo, agradecerte el programa, la oportunidad de haber participado en él (O.1) y bueno, que te va a salir una tesis muy chula y que creo que al final lo más importante tanto en nuestros entrenamientos como en este programa que al final no deja de ser un tipo de entrenamiento, es que nos sirva y que mejoremos los que, los que lo hacemos ¿no? Me imagino que tu (ininteligible) un montón haciendo el programa y te darás cuenta de cosas y nosotros que somos los que lo recibimos pues, a mí por lo menos me ha servido para darme cuenta de muchas cosas (PE.4) y de como gestionar mis emociones (AG.2) e identificar en gente lo que quiero o no quiero o lo que a mí me gustaría que me escucharan para yo después poder (ininteligible) a la gente (RI) ¿no?

**E: (sonido de asentimiento). En esas relaciones interpersonales, ¿no?**

P: Claro. Sí, sí. Por ejemplo, todos tenemos un amigo, un familiar que lo sabe todo, que él tiene siempre razón, que… y al final, bueno, pues como tu mismo has dicho al principio de esta charla, ¿no?, no hay respuestas buenas o no, sino que son diferentes puntos de vista y al final, hacer de cuñado y echando el café pues todos podemos tener razón.

**E: Está claro. Joan. Pues muchas gracias a ti.**

P: Vale Aleix.

**E: Nos vemos esta tarde.**

P: Vale, muy bien.

**E: Un abrazo.**

P: Un abrazo Aleix.

**ENTREVISTA 4. SERGI (Director Academia Internacional, 38 años) – Código P.4**

**Realizada *online* el día 6 de abril de 2021**

**E: Ets Sergi, director del projecte local de Turquia, no?**

P: Istanbul.

**E: Perfecte, Sergi.**

1. **E: Com diries que el programa de *mindfulness* per entrenadors t’ha impactat, si és que ho ha fet d’alguna manera?**

P: Doncs m’ha impactat molt positivament (O.1), perquè emm... m’ha fet veure les coses des d’una altra perspectiva... (CT.2) perspectiva que no sempre comparteixo, però que si més no m’ha fet reflexionar moltes coses(CT.2), i m’ha fet també adonar-me d’alguns perquès, sobretot d’alguns perquès dels meus comportaments (PE.4) i... i realment *pues* això... l’he gaudit molt (O.1), i entenc que m’ha aportat molt coneixement (CT.1), molt coneixement sobretot sobre mi mateix (PE.4).

**E: Autoconeixement, no?**

P: Si.

**E: Molt bé. I a nivell personal o... entenc que això ho has dit bastant a nivell personal no?...a nivell professional destacaries alguna cosa?**

P: Si...

**E: O ho englobaries tot... Digues, digues.**

P: No, eh... també, també... jo sóc una persona molt pràctica... i molts cops quan t’estic escoltant a tu explicant qualsevol cosa a la classe, jo me n’estic anant a “això quan ho puc dur a terme? Això per què em pot servir?”. I hi ha moltes reflexions que tinc la sensació que realment em poden ajudar a ajudar als altres...i estic convençut que tant en formacions als meus entrenadors, eh... parlant d’això... tant emm...eh... amb intentar pues això, que els meus jugadors sàpiguen tenir un focus en allò del que estan fent, en centrar l’atenció en el que toca... eh... a més estic en un país que és super emocional, vale? I jo crec que moltes reflexions d’aquestes m’aniran molt bé per això, per poder-los centrar (R1.1).

**E: Okay...**

P: Al final jo em veig una miqueta reflexat amb la... com t’ho diria... amb la forma de ser que tenen aquí a vegades els turcs i les mateixes reflexions que m’he fet jo entenc que els hi podem traslladar a ells (R1.1).

1. **E: Super. Un segon...Vale, emm... la segona... és si has modificat o has incorporat algun comportament, estratègia, aprenentatge en la teva vida?**

P: Emm, sí... et diria que sí, et diria que sí... emm... sobretot, com t’ho diria... sobretot, en adonar-me on tinc el cap. És a dir, em dona la sensació que sóc capaç de veure molt més que m’he desconnectat... (PE.4)

**E (So d’assentiment)**

P: Vale? Que ja no estic... que no estic això... i també m’adono molt més de, vale, ara igual m’estic desconnectant perquè potser necessito descansar, necessito parar una miqueta... (PE.4) perquè es una cosa que mai feia, jo anava tirant (AG.3) i després feia que molts cops em passava una tarda que realment potser amb una hora l’hagués pogut fer, vale?

**E: Ja...**

P: I ara realment entenc, bueno, potser no és el moment... (AG.3)

**E: Okay...**

P: ...vale, potser necessito d’això i... i me n’adono molt més PE.4) també quan desconnecto en converses (RI.2) o en qualsevol situació me n’adono. Fet que jo crec que fa que em pugui reconnectar més ràpid.

**E: Exacte. A nivell laboral, explicaries alguna estratègia, comportament...?**

P: És que a nivell laboral, realment fins ahir que vam poder tornar a entrenar he estat eh... pràcticament... l’únic que et puc dir és això, és en reunions que he tingut que realment que sí que me n’adono quan em desconnecto, vale? I...

**E: Vale...**

P: ...i però que a partir d’aquí no he tingut massa...perquè a més a més, ahir vam tenir una reunió i vaig deixar als coordinadors que la fessin, perquè era més de logística, i realment no ho he pogut posar en pràctica encara.

1. **E: Super. Eh...mm...a nivell de valoració del curs, què diries que es el que ha sigut el més positiu per tu?**

P: Jo crec que la quantitat d’informació d’interès (CT), de fonts que ens has passat, de vídeos, de llibres... (EO.3) bueno, de llibres no me n’he llegit cap, però igualment és... saps allò que dius quan tingui temps i espai...i vídeos si que me n’he mirats molts...molts vídeos, moltes referències... jo crec que això... totes les reflexions que hi han darrere... al final no és això, és la reflexió que hi ha darrere, jo crec que això és impegable (CT.2).

1. **E: Super... quins creus que han sigut... has tingut... quins han sigut els reptes o dificultats, si és que n’hi han hagut, al completar el programa?**

P: Emm... doncs al final, una mica la sensació de ser... el com t’ho diria... no et diré l’anticrist.,. Però una mica el... el...

**E: L’advocat del diable...**

P: ... si... no... o a vegades el neandertal del grup, saps? Amb pensaments així molt allunyats de la tendència amb gent que portava més temps emm... doncs això, practicant el *mindfulness* i que tenia més coneixement i jo *pues*... realment, en moltes opinions em sentia una mica sol, vale? I... això a vegades em tirava endarrere. Dic, “no sé si saps al final eh... és bo que sempre vagi portant la contraria”, es pensaran, saps? Que sóc un desagraït o qualsevol cosa...

**E (rient): Ja em passa...**

P: Em feia sentir una miqueta eh... saps, eh... malament, però, però a part d’això res...

**E: Perfecte...**

P: M’ha agradat molt i he estat molt content i molt feliç (O.1).

1. **E: Molt bé, emm... en quina mesura ha coincidit aquesta formació amb les expectatives inicials, si és que en tenies?**

P: Eh... clar, és que no sé... et diria que les he superat, però es que tampoc recordo exactament quines... saps, o sigui... el que et puc dir és que estic molt content... (O.1) més enllà de dir-te si ha superat o això...perquè això em costaria ser sincer...

**E: Ja...**

P: ...perquè no sé quin pensament tenia en aquell moment...d’això el que et dic és que estic molt content i que el tornaria a fer segur, segur... (O.1)

1. **E: Vale... Què destacaries del curs? No sé si es una mica repetitiva la pregunta, però si ha quedat algo del que destacaries del curs?**

P: Et diria lo d’abans i després també una altra cosa, emm... també, emm... les reflexions... més que les reflexions, sí, les reflexions personals que alguns companys heu compartit doncs això, m’han colpit molt... i fins i tot, en alguna situació també no he trobat el moment per compartir alguna, jo que sé... vivència meva més profunda que potser jo hagués pogut aportar...aportar també jo el meu granet de sorra aquí... però, però m’ha colpit molt això... algunes vivències personals, experiències personals que les sentia molt profundes que realment hi veia molt sentiment i... i m’han colpit molt i m’han agradat molt (O.2).

1. **E: Molt bé, brutal... ja anem acabant. Com valores aquest programa en el teu rol professional?**

P: Molt important. Em sembla que és importantíssim... també faig aquesta reflexió moltes vegades que si jo com a jugador, algun cop hi hagués hagut molta feina...

**E. (So d’assentiment)**

P: ...però si hagués tingut la oportunitat d’arribar a aquest tipus de reflexions, el meu rendiment hagués sigut, vamos, 800 vegades més alt! Perquè era un jugador que no sabia controlar les emocions, que realment tenia una mentalitat molt negativa, sempre un pensament negatiu, em...que qualsevol situació em superava emocionalment... i jo no ho entenia...clar, no... (AG.5)

**E: No tenies eines, no?**

P: No, i a part un partit de 3a regional, jo no podia dormir dels nervis...de 3a regional, que dius home...

**E: Clar...**

P: ...emm, doncs això i jo que sé... jugar un partit i jugava absolutament, saps o sigui... fora de mi... doncs tot això jo crec que m’hagués anat super bé.... i em sembla que ara mateix, també, és a dir...crec que he madurat una miqueta i he crescut però igualment me n’adono que totes aquestes reflexions de com controlar (PE.3) i de conèixer-me a mi mateix i de quan genero una resposta, perquè ve aquella resposta, perquè tinc ira, perquè tinc ràbia,... doncs em va molt bé per conèixer-me i tenir millors reaccions... (PE.4) i també per ser més empàtic amb la gent... (RI.1). Em sembla una cosa importantíssima i que el futur va per aquí (CT.4).

**E: Vale... ja la última...**

P (rient): No me n’has fet 10 eh...

1. **E: Si, aquesta és la 10, el que passa es que he anat entre professional i personal i les separo en dos... Quines millores implementaries al programa, si és que ho faries?**

P: Jo, em...potser eh... el faria una miqueta més llarg i... podent...com t’ho diria, aprofundint en algunes situacions... (EO.4) per exemple, també tenia algun cop la sensació... al principi vaig començar parlant molt, vale?

**E: (So d’assentiment)**

P: Potser massa! (Riu)

**E: No, per això ho valores tu...**

P: Ja, ja...eh.., i després també em dona la sensació que si parles o si ens fiquem aquí d’això realment hi ha molta informació que vale, per escrit està molt, bé però que m’agradaria... pos això, entendre-la o d’allò i que havíem d’anar ràpid perquè no arribàvem...

**E: Ja...**

P: I en algunes situacions et veia a tu dient “Buf! Tinc 25 diapos, queden 15 minuts, pam, pam, pam, pam...” i a mi m’hagués agradat una mica més doncs això... ja sé que és difícil i que probablement no tenim l’espai tots com per tenir unes sessions més llargues, però m’hagués agradat poder anar més tranquil i assaborint-t’ho més.

**E: Vale. Més llarg diries...fer la sessió més llarga i estendre-ho en setmanes, no?**

P: Si, si...

**E: En realitat això es podria mantenir...**

P: Jo el que et volia dir, perdona, més que més llarga, més setmanes... (EO.4) perquè més llarga puc entendre que la gent igual no hi pot dedicar... parlant amb la... posant-me el (inintel·ligible) al cap, l’horari del (inintel·ligible) al cap... que a vegades és complicat treure moltes hores seguides perquè sempre tens coses, vale? Però estendre-ho amb setmanes, potser 10, no ho sé eh, això ho sabràs tu molt millor...

**E: Si, si, si...**

P: Però això crec que si que m’hagués... almenys a mi m’hagués agradat molt.

1. **E: Super...Doncs si vols afegir algun comentari...**

P: Doncs res... que una de les coses, una de les motivacions també que vaig tenir a l’hora de fer el curs és que tu el feies (EO.1), vale? Perquè, doncs perquè tinc molt bon record de quan vam coincidir a Barcelona... recordo que, que això, que vas ser una persona que emocionalment allà també em vas ajudar molt, perquè ets una persona que recordo que reia molt, que ets molt alegre, que intentaves sempre ajudar a tothom, que generaves un clima super sa, super positiu... i que per mi això va ser un fet tant important com el contingut del curs...

**E: (So d’assentiment)**

P: És a dir, el contingut del curs em va agradar, perquè és un tema que m’agrada, però si m’arribes a fer el curs de la petanca finlandesa

**E (Riu**

P: ...jo m’hagués apuntat al curs de la petanca finlandesa... i jo crec que això també és molt important a l’hora d’aprendre qualsevol cosa, és a dir que la persona que tens al davant, t’agradi o no t’agradi (EO.1).

**E: Hm....moltes gràcies Sergi...**

P: De res...

**E: ...el mateix, estic molt content de que hagis vingut, de que hagis assistit, perquè també tinc molt bon record de tu, i endavant... a veure com tira això... Jo ara paro gravació...**

**ENTREVISTA 5. JOSEP (28 años, Director Academia Internacional) – Código P.5**

**Realizada *online* el día 6 de abril de 2021**

1. **E: Vale, llavors Josep, tu ets DPL d’un dels projectes internacionals de l’escola del Barça i has participat al programa de *Mindfulness* per a entrenadors/coordinadors’, entre els qual estaries tu. I la primera pregunta és com diries que el programa t’ha impactat, si és que ho ha fet d’alguna manera?**

P: Eh, sí. El que m’ha impactat bàsicament ha sigut el obrir-me a un món nou que no coneixia (CT.1), no? Jo sí que fa temps que estic treballant amb una psicòloga per certs aspectes que jo creia que havia de millorar, etc. Més a nivell mental, però en cap moment, sí que vaig veure o he vist algunes coses en comú, però la perspectiva de fer-ho a través d’una estratègia com la meditació (PE.1) o amb l’idea de ficar una atenció plena en el que estàs fent i d’intentar, no sé si controlar els pensaments o almenys observar-los i donar-te compte del que estàs pensant o de com estàs interactuant en aquell moment (PE.4), doncs això sí que m’ha ajudat molt a obrir, no, en aquest sentit a veure-ho en aquesta perspectiva (CT.2). Llavors, quan ens ho vas presentar, li vaig veure com un sentit o com un complement que em podria ajudar a mi a nivell personal primer...

**E: (So d’assentiment)**

P: ... i en tot aquest procés que jo estic passant i, òbviament, si ho podia afegir en el meu nivell personal i em podia anar bé, llavors doncs òbviament jo podria ajudar a altres persones en algun tipus d’impacte en altres persones (RI.1) i pensant ara en el projecte de Índia o almenys veure les altres persones també des d’un punt de vista, no? De l’error, de coses que, de comportaments i valors per exemple...

**E: (So d’assentiment)**

P: Sé que m’ha ajudat en aquest sentit, a obrir, no? Llavors, també a nivell de pràctica és una cosa que no havia fet mai i al principi sí que em costava més que ara, suposo que ara estic una mica més acostumat sense... portem ara, bueno des de que hem acabat jo segueixo meditant, 7 setmanes ara farà... bueno, suposo que és un hàbit que encara s’ha de seguir però no sé... em relaxa i m’ajuda en aquest sentit a donar-li un moment de pausa al dia (AG), no? De dir, anem amb 50.000 coses i és un “para, pensa amb tu, pensa amb el que estàs fent i segueix...” (PE.2) llavors, diria que en aquest sentit m’ha ajudat d’aquesta manera.

1. **E: Molt bé. A veure... has modificat, incorporat algun comportament, alguna estratègia o algun aprenentatge, tant en la teva vida com en l’àmbit laboral?**

P: Eh... a nivell laboral, ho feia de forma diferent, però sí que els blocs de 45/15 o 50/10, 60/20... ai, 90/20 (PE.3), etc. els intento aplicar, encara estic “en ello”...hi ha alguns dies que m’oblido i estic hora i mitja o dos hores connectat i després em dono compte i dic ostia! El punt aquest d’anclatge (PE.3), no? Anem a fer 10 minuts de pausa i seguim...

**E: Però aquí, voldria remarcar que si tu estàs connectat, segueix. Les pauses són més per aguantar el bloc de treball seguit, no tant perquè sigui necessària la pausa, m’explico? És a dir, que si quan anem cap a 90 la pausa ens va bé, però si tu pots estar 90 o 120 i estàs bé, no cal la pausa. El que busquem és mantenir-nos un bon rato amb atenció plena.**

P: Vale. Doncs en aquets sentit ho intento aplicar. Sí que hi ha moltes coses, com per exemple comportaments (AG.3), el donar-te compte a una conversa emm.... com l’altre es sent o com l’altre interactua... és algo que intento (RI.2), però si que és veritat que hi ha alguns moments que jo pues tinc unes emocions més negatives o més en aquest sentit que intento controlar, però com que jo m’estic intentant gestionar a mi mateix (AG) potser no li presto atenció a l’altre de com es sent. Llavors en aquestes coses estic “en ello”, intentant-ho aplicar en si, ho estic intentant aplicar o almenys ser més conscient (PE.4) d’això en certs moments “ostia, això no ho estaves fent, intenta-ho fer”, però si que hi ha moltes dinàmiques que he de seguir aprofundint. Per exemple, hi va haver una que vas proposar dels valors, no?

**E: Sí.**

P: De tu mateix pensar com, pues el respecte com es fa en diferents situacions, això com ho faràs aquí o allà... aquestes dinàmiques si que és veritat que haig de... no he anat molt a fons en elles, no ho he aplicat ni ho he exercitat o res en aquest sentit i sí que hauria de seguir profunditzant en aquest sentit, no? Llavors hi ha algunes coses que sí i moltes que no encara. Suposo que es un procés i anar poc a poc, seré sincer; més que no que sí.

**E: Si, no, perfecte. I a nivell de vida, has incorporat alguna estratègia, algun comportament... més concretant a nivell de vida?**

P: A nivell de... més personal?

**E: Personal, sí.**

P: Sí, el... jo he passat una època de molta ansietat i molt estrès i encara suposo que estic “en ello”, no en si, però en saber-lo gestionar i en diferents tècniques de relaxació en aquest sentit i de també dominar els pensaments, no? Per moltes coses que tenia que eren més tipus somàtic, que a través dels pensaments m’ho podia controlar i fins que no m’he donat compte ha passat bastant temps. I ara estic en el procés d’encara que la meva ment ho pensa i dic “ostia, no pensis això que no és així” i estic en aquest procés de diàleg intern no? No t’estressis o mira-ho des d’aquesta perspectiva...

**E: Sí...**

P: Relativitzar una mica més les coses en aquest sentit... llavors et diria que a nivell personal estic així, i això, òbviament, m’afecta les relacions, no? Perquè jo si estic estressat o nerviós, com actuo amb els demes és més de defensa, saps? Com un acte més de defensa, com menys empatia, com menys...

**E: (So d’assentiment)**

P: ... parlar més malament amb els que tens més a prop, no? A vegades és el que passa no, que ho pagues amb els que ho tens més a prop. I a través d’aquest procés m’he donat compte que allà no he actuat bé i tal i potser l’endemà he anat un moment i dir ”ei, em sap greu per lo d’ahir” i ja està, no? (AG.4 / RI.4)

**E: Molt bé.**

P: Estic en aquest procés, la veritat, ara mateix. De primer saber-me dominar a mi mateix o gestionar les emocions, tenint en compte que “ei, això és el que estàs fent”, perquè potser abans no volia veure-ho, o no ho veia.

**E: (So d’assentiment)**

P: I ara és com “està passant això”, com ho anem a direccionar.

1. **E: Molt bé. Què diries que ha sigut lo més beneficiós per tu?**

P: Ostres, et diria que el procés en si. Potser es un tòpic eh, però del programa jo crec que el més beneficiós, jo crec que una cosa i... vaig... l’altre dia vam quedar amb el Sisco, vam anar a fer un cafè i vam estar parlant de varies coses. I una de les coses que em va impactar més va ser l’escoltar els companys que també tenien, no problemes, no sé com dir-ho, però tenien les seves situacions, com tots tenim. Llavors com que em va tocar amb un grup que si ho fiquem amb un rang de posició, com a mínim igual que jo o la majoria per sobre meu, de CC’s o no sé al DPL a Nova York o “whatever”. Era com primer de respecte, de “ostia, vigila què dius aquí, potser lo que dius aquí, saps? Pot tenir...” primera tenia aquest pensament, no sé... però després quan vaig començar a veure com tothom s’obria, començava a compartir coses i tal... és com que això em va donar un impacte de dir “al final tots som persones i tothom tenim lo nostre”, i per molt que tu pensis que ell és a la posició que sigui, ell també els seus problemes o els seus d’això igual que tu tens lo teu i un altre té lo seu. Llavors, doncs aquest compartir, en aquest sentit i veure des d’aquesta perspectiva és una cosa que em va impactar com a positiu, no? I llavors també em va donar com a mi calmar-me més de vaig compartir poc, però compartir algo (O.2). Llavors, una de les coses que em va agradar més, perquè en general a mi és el que més m’agrada, no? Et donen o t’obren a coneixement nou, o almenys a veure-ho des de perspectives diferents, o més científiques, etc. Llavors les presentacions, els principis, els valors, les diferents coses que vam anar parlant... sí que hi ha molta cosa que, no sé si ho tens interioritzat o no, però ho coneixes, però ho coneixes des d’una perspectiva diferent. O que t’ho transmetin des d’un punt de vista diferent o *linkar-ho* des d’aquesta manera, crec que es lo que més emm... (CT) junt amb lo altre, el que més em va agradar. Em, si, et diria això... bàsicament les dinàmiques dels dilluns.

**E: Perfecte. Que vols dir amb les dinàmiques dels dilluns? El dia en concret de sessió, no?**

P: Sí, la sessió que teníem. El fet de que tothom compartia i al final i les creences limitants que potser jo tenia al principi em van desinhibir i després va donar peu a sentir-me diferent i a compartir...i el tenir accés de coneixement des d’una altra perspectiva i linkar-ho amb això del *mindfulness*, què és això del *mindfulness* i obrir una mica aquest camp que no coneixia.

1. **E: Has tingut o quins han sigut alguns dels reptes o dificultats per completar el programa?**

P: Sí, primer agafar l’hàbit de meditar, de fer-ho cada dia (PE.1). Hi havia com dies que em feia mandra, però bueno, pensava “s’ha de fer”, llavors ho intentava fer, però llavors no és el que s’ha de fer perquè s’ha de fer, sinó busca-l’hi un sentit en això, no?

**E: (So d’assentiment)**

P: Llavors, com tenir aquest diàleg intern jo i agafar l’hàbit de comprometre’m per obtenir un benefici. Llavors, et diria que això seria un. El segon va ser fer-ho al mateix temps, o sigui a les mateixes hores... quan et lleves al matí , a les tardes, als migdies o a l’hora que fos. Lo ideal potser seria més al matí, per afrontar el dia, quan et lleves...

**E: (So d’assentiment)**

P: Llavors, jo et diria que aquest seria el segon repte.

**E: Com li anomenaries, el trobar el moment?**

P: Sí, sí, potser el trobar-li el moment idoni (PE.1) per la meves circumstàncies específiques. Potser a mi, per dir-te algo, em va bé al migdia o a mig matí, perquè començo a tope amb els reunions i no sé què i després necessito un *break* per seguir-me focalitzant amb lo que ja vindrà i jo crec que potser a un altre li va millor al matí o a la tarda, no? M’ho invento... el descobrir el que em va millor i agafar aquest hàbit. Aquesta sigut un dels reptes més importants. Llavors, un altre repte és seguir les dinàmiques, els “deures” aquests setmanals... (AP)

**E: La proposta d’entrenament...**

P: Exacte, no només la meditació, sinó les diferents dinàmiques que teníem com “ara has de tenir atenció plena quan mengis, a la dutxa...” (PE.2)

**E: (So d’assentiment)**

P: O “fica’t en aquesta dinàmica a veure com et sents, com ho veus, etc.”. Llavors aquesta part és la que m’ha costat més i també t’he de ser sincer que és la part que he fet menys. La meditació sí que l’he intentat fer algun dia si no l’he fet i allí al registre d’entrenament està posat, està en blanc però ho he anat fent majoritàriament, però l’altra part sí que majoritàriament no l’he fet, algunes vegades sí que m’hi vaig posar. Llavors els tres reptes més grans et diria...

**E: Serien aquests... això és per aprofundir una mica. Et sembla excessiva la part d’entrenament o et sembla que està bé?**

P: Jo al principi quan ho vaig llegir i després ho vaig compartir amb alguns companys que també estaven fent el programa i ja els hi vaig preguntar... perquè al principi em pensava que eren com moltes meditacions al dia, o ho vaig entendre jo malament...

**E: Ja...**

P: ... i dinàmiques. I com que em vaig agobiar i vaig dir “uf, no tindré temps a fer-ho” o “ho veig molt”. Llavors vaig dir “vosaltres que heu entès aquí?” i em van dir “no, no un per dia, no sé què no se quantos...”. Jo potser com a volum de dinàmiques (AP), potser sí que ho vaig trobar a vegades... però també et dic això i després jo vaig ser el primer que moltes no les vaig fer, o sigui que no seria just això...

**E: Ja, ja, ja...**

P: Però potser sí que vaig tenir la sensació que, així com la dinàmica de la meditació va anar com gradualment no? 10 minuts només després 15, 25 i així... i que a mi em va ajudar en aquest sentit, potser lo altre de dinàmiques complementaries potser a mi se’m va fer una mica més feixuc en aquest sentit, personalment.

**E: Aha...**

P: Però ja et dic, tampoc no les vaig seguir i per això et vaig ficar que m’agradaria seguir-hi aprofundint... (EO.4) més que res, primer fer les dinàmiques no? Completar algunes dinàmiques que no vaig fer i aprofundir en aquest autoconeixement (PE.4), que en certs aspectes és com que em fa por, no? I l’altre dia ho parlava amb el Sisco, jo necessito molt com una aprovació externa i ara si jo em poso a pensar en mi mateix, a avaluar-me, a els valors com ho fas... vale, jo sóc això, però dades objectives que siguin exemple del respecte, per exemple, no? Pos he fet això, això o lo altre... és, com quan acabi, pensaré “i què? És veritat o no és veritat? És com que sempre necessito una aprovació externa “no, no jo crec això” o “jo estic amb tu” o “no, no, t’estàs flipant, no és així” o lo que sigui.

**E: (So d’assentiment)**

P: Llavors és com que tinc la por o incertesa aquesta de que, com que sé que necessito una aprovació externa i no li dono molt valor a la meva aprovació interna, potser és una cosa que he d’anar canviant. Però bueno, per això ho deia.

1. **E: Okay. Expectatives, en quina mesura ha coincidit aquesta formació amb les teves expectatives inicials, si es que en tenies?**

P: Expectatives inicial tenia, estava curiós de que seria i com això em podria influenciar i a millorar... i ajudar a millorar. I la veritat que tampoc tenia unes expectatives molt altes, tampoc esperava, no sé, no esperava gran cosa, estava més amb la curiositat aquesta de dir “ostres, això que serà, em convenceràs o no?” M’explico?

**E: (So d’assentiment)**

P: I la veritat que sí, ho he vist bé, m’ha ajudat i crec que m’ajudarà si ho segueixo fent i segueixo aprofundint, perquè és una cosa que jo veig que haig de fer més personal jo...

**E: Ja...**

P: ... jo tinc interès i vull fer-ho i jo m’haig de comprometre amb mi mateix a fer-ho i a canviar certes conductes i certes creences limitants i coses d’aquestes a través de l’autoconeixement. I...però sí , en aquest sentit jo crec que molt bé (O.1).

1. **E: Súper. Què destacaries del curs, valorant com lo millor?**

P: Què destacaria de lo millor?

**E: Sí. Què es el que més t’ha agradat o destacaries positivament del curs?**

P: Ostres, potser el...o sigui, amb lo que he comentat abans dels dilluns no? Potser el compartir, el compartir experiències i el veure com, no sé, per exemple el Sisco em va explicar lo de la (intel·ligible) i bueno, allò va ser algo que per ell li va influenciar molt i com ell ho va gestionar. Llavors el compartir aquestes experiències i el veure com s’ha anat gestionant, jo crec que és lo que més m’ha... o m’ha agradat, o m’ha influenciat, o m’ha impactat i... en el sentit d’això, no? De que tothom tenim reptes i que és de com s’afronten per donar-li la volta, no? (O.2)

1. **E: (So d’assentiment) Com valores aquest programa pel teu rol professional?**

P: Jo crec que és imprescindible. És imprescindible perquè si no et saps gestionar a un mateix (AG), és molt complicat gestionar o intentar... potser gestionar no és la paraula, però intentar impactar o influenciar en els altres, no? (RI.1)

**E: (So d’assentiment)**

P. Llavors, per mi, i ho he pensat sempre i cada cop em reafirmo més amb lo que penso, que un bon lideratge és la base de tot. És a dir, tu pots saber més o menys d’un tema i això, però si et convenço, si jo et convenço a tu de que això és lo millor, tu t’ho creuràs, no? (RI.1)

**E. (So d’assentiment)**

P: I a lo millor no és lo millor, potser és una altra manera de fer o una altra historia. Llavors, aquest lideratge (RI.1) si ho veiem a través de l’autoconeixement de saber les teves limitacions o saber els teus punts forts i a partir d’aquí les teves limitacions (PE.4) potser les podràs delegar a altra gent que és més competent que tu en aquestes coses i al revés, no? Llavors crec que això farà que (intel·ligible) amb el teu equip i que tothom es senti important (RI.1) i etc. Llavors, jo ho veig imprescindible, si, si... (O.1)

**E: Molt bé.**

P: I també el...

**E: Digues, digues.**

P: No, també et dic el... que això m’arriba 5 o 6 anys abans, per exemple la exposició aquesta que ens vas fer per explicar-nos així de forma general el projecte i qui es vol apuntar i tal i qual... si a mi m’ho expliques fa 5 o 6 anys, jo segurament no li hagués donat ni importància. O sigui, hagués escoltat i així, però no m’hagués apuntat, no? Crec que el moment en que t’arriba, per una cosa o altra, quan t’arriba... doncs t’arriba, no?

**E: (So d’assentiment)**

P: I s’ha d’aprofitar, no? Però si que ho veig realment imprescindible d’autoconeixer-te tu mateix i saber tu com ets i tu com actues (PE.4), per saber liderar d’una forma més òptima el teu equip (RI.1).

1. **E: Súper. I... vale. La última és quines millores implementaries en el programa, si és que ho faries?**

P: Eh.... bé, no sé, no em veig amb la capacitat de...

**E: Pot ser a nivell general eh? Dels *timings*, donar... no sé...**

P: La veritat és que no hi he pensat eh... jo crec, et diré de les coses que a mi m’han agradat: 6 setmanes i 1 cop per setmana ho veig bé, perquè és com que agafes responsabilitat d’estar-hi però no et treu moltes hores i estan ben aprofitades (EO.1). I jo prefereixo un cop a la setmana i 1 hora i mitja o 1hora i 45 si s’ha de fer, que 2 dies de 1 hora, realment... jo això ho veig bé. 6 setmanes és una durada que se’t fa relativament curt, perquè, bueno hi ha moltes coses a fer i tal i qual i 6 dilluns passen molt ràpid... i no sé, les dinàmiques el que t’he dit, a mi m’agrada el compartir experiències, tal i qual... és que no sé el que milloraria...

**E: No, perfecte...**

P: La veritat és que no ho sé.

1. **E: Vale. I la última, si vols afegir algun comentari final? Algo que no t’hagi preguntat potser i tens ganes de dir o el que sigui?**

P: Bueno, no, seria més relacionat amb aquesta aprovació externa o interna. Parlant-t’ho amb el Sisco, comentàvem que si tu tens una aprovació interna teva, perquè necessites ningú que t’ho aprovi, no? Però bé, no sé, estic en aquest moment de dir de com puc arribar a canviar no, de donar-li més importància a lo que jo pensi i si jo m’ho aprovo o no a nivell intern... a que m’ho aprovi un segon o un tercer, o una persona externa bàsicament. Estic en aquest punt de dir, no sé cap a on, suposo que primer és conèixer-me, o conèixer-me més i després no sé, un cop em conec més, després què, no? Aquesta és la pregunta que tinc...

**E: Bé, no, jo crec que és, primer, adonar-te que això funciona així, i després quan tu observis els teus pensaments que estan buscant una aprovació externa, simplement adonar-te’n i deixar-ho anar i prendre les decisions ne base en què tu creus. Jo crec que és adonar-se’n i que el teu pilot automàtic funciona així, però que tu saps que no la necessites, no? Tot i que al final, em ressona molt a mi, jo també sóc una persona que necessita del reconeixement extern... i poc a poc, és anar-se’n adonant de que potser no fa falta no? Perquè des del reconeixement extern moltes vegades et fa modular la teva actitud en base a la aprovació externa i no en el que creus que hauries de fer i aquí és quan comences a jugar un rol que no és el teu i quan comences a entrar en contradiccions... i acabes sense acontentar a un ni als altres, saps?**

P: Cent per cent, cent per cent...

**E: Josep, pues moltes gràcies.**

P: A tu, pel programa i per obrir-me un món nou.

**E: Una abraçada, Josep.**

P: Vagi bé Aleix, merci, adéu.

**ENTREVISTA 6. ANDREU (Director Academia Internacional, 31 años) – Código P.6**

**Realizada *online* el día 7 de abril de 2021**

**E: I per confirmar, tu ets l’Andreu i estàs de director de l’acadèmia del Barça a Rússia, estàs a Moscou.**

P: No, no ara no estic a Rússia, estic a Barcelona Aleix.

**E: Ah, sí?**

P: Sí, ja no estic més al Caire ni a Moscou.

**E: I que estàs fent a Barcelona, Andreu?**

P: Ara començaré un nou projecte, però estem a la espera a veure si puc començar o no.

**E: Vale, però amb el Barça eh?**

P: Sí, sí, amb el Barça. Nou destí i veurem.

1. **E: Sí, és un any complicat. Vale Andreu, la primera pregunta és a veure com el programa t’ha impactat, si és que ho ha fet d’alguna manera?**

P: El programa m’ha impactat de la manera que m’ha fet conscient de coses que abans no ho era, emocions, el tema de ser assertiu amb les persones. Hi ha moltes coses que (inintel·ligible) *mindfulness*, estaven relacionades amb aquest tema, m’han fet prendre consciència diguem-ne (PE.4). Per exemple, l’altre dia estava llegint un llibre i, no sé, moltes vegades per mi era normal llegir un llibre (PE.2) i tornar a començar 10 pàgines enrere perquè no me’n recordava de què havia llegit. I ara és com, “no, no, estic llegint” i en el moment dic la paraula “presente”, centrat en el moment (AG.1). *Es como, lo digo en voz alta que si alguien está al lado mío y dice “está loco”*. Fins i tot al gimnàs, l’altre dia també estava al gimnàs (PE.2) i en un moment, sense el mòbil ni res, dic en veu alta “presente” i començo a... (gest d’aixecar peses)

**E: (Riu) Vale.**

P: Hi ha moltes coses que ja feia, però ara és com que me n’adono... (PE.4) no sé, hi havia una diapositiva que era incompetentment incompetent, incompetentment competent i competentment competent. Llavors, ja et puc dir que estic més cap a la dreta... i abans (inintel·ligible). Per exemple, Mario Alonso he vist un munt de vídeos... (inintel·ligible) però no he tingut un, anem a dir, un mentor com tu que m’ajudés a realment cada setmana recordar-m’ho. Algú que cada setmana et recorda que ho has de fer (EO.1).

**E: Destacaries alguna cosa a nivell professional, per exemple?**

P: Mmm...

**E: Com diries que el programa t’ha impactat a nivell professional?**

P: Jo crec, m’ha impactat més, el que deia a nivell personal i això ha fet que a nivell professional m’adonés del meu estil de lideratge (RI.1) potser. Que tinc aquesta percepció d’intentar sempre leer el lenguaje no corporal (RI.2) y es algo que ya hacía, pero ahora lo hago más todavía. Es como que antes lo hacía porque sabía que era importante, pero a través de este curso como no, no... ya sabía que era importante pues ahora con lo que puedes aprender de Aleix sí que me tengo que fijar más aun todavía.

1. **E: Molt bé... Següent pregunta. Has modificat o has incorporat algun comportament o alguna estratègia o algun aprenentatge, tant a nivell personal com a nivell professional?**

P: Em... sí, hi havia moltes coses que comentava abans que ja les feia. Per exemple, el tema del estoïcisme, és que abans d’anar a dormir sempre apunto el que tinc al cap per anar a dormir sense, sense tindre res al cap. Però ara és com, també ho faig, però ara ho faig perquè sóc conscient de que estic vivint al moment present (AG.1) i me’n vaig a dormir sense pensar en res més. I abans ho feia, però no com amb el *mindfulness*, i ara pel mati m’aixeco i tinc la llista de coses del dia anterior i puc començar el dia millor.

**E: Sí, no... diria que potser el que t’ha donat és un anclatge general, no? De dir: ostres, et fa el recordatori aquest de tot el que anaves sabent i practicant, però potser amb una mica més... més...**

P: I més conscient! Jo la sensació que tinc ara és que faig moltes coses, com et deia abans el *mindfulness*, perquè fins quan estava a Rússia fa 2 anys enrere, també volia fer-ho, però no sabia com fer-ho, no tenia un.... I ara és com que tinc consciència del que faig o per què ho faig, que crec que és el més important per mi... el per a què faig les coses (AG.1). No només ho poso en pràctica, sinó en base, a una base no sé si científica o a... a un concepte diguem-ne.

1. **E: Perfecte. Què diries que ha sigut el més beneficiós per tu?**

P: Eh....per mi el tema de la meditació (PE.1), el tema tant senzill de la meditació de assentar-me 10 o 20 minuts a fer meditació, perquè a mi és el que més... només el tema de la meditació, tant senzill com fer les respiracions (PE.1) m’ha aportat molt (O.1).

**E: Molt bé.**

P: Perquè és una cosa que crec que no he aconseguit hacerlo solo, cuando lo he hecho solo no he podido, porque me frustraba, porque no sabia si los pensamientos se me fueran estaba bien, si no centrarme en el momento presente estaba bien... y contigo repetías una y otra vez que no pasaba nada si en las primeres sesiones os vais, si no respiráis, si perdéis el anclaje... y eso me quitó mucha presión de encima de decir “tu ves haciéndolo, que cuando lleves varios días te irá mejor”, ¿sabes? Pero antes era como, “lo estoy haciendo mal” ... O incluso era “no sé si lo estoy haciendo bien o lo estoy haciendo mal” i amb el tema d’aquest de recordatori de “no passa res si te’n vas…” Ah, vale perfecte... (EO.1)

1. **E: Perfecte. Quins han sigut alguns dels reptes o dificultats que t’han sorgit per completar el programa, si és que els ha hagut?**

P: Sí, bueno aquí he tingut 2 *challenge*, que vaig a dir de forma positiva, 2 *challenge* (riu)

**E (riu)**

P: El primer desafiament ha sigut que per tema horaris. Sóc conscient de que sinó ho faig només aixecar-me, ja no ho faig en tot el dia. No sóc capaç de trobar un moment, i no vaig a dir que no tinc temps, sinó que es una gestió de prioritats. (Riu)

**E (Riu): Molt bé, molt bé!**

P: I el segon, és que casualment he tingut al mateix temps *un desafío personal a nivel de pareja y esto ha hecho que la cabeza este un poco más con este ruido mental*...

**E: Si...**

P: *Y que muchas veces ha sido “es que para que me centro, necesito ir a correr, ¿no..?” pero bueno...*

1. **E: Vale. Expectatives, en quina mesura ha coincidit aquesta formació amb les teves expectatives inicials, si és que tenies expectatives?**

P: Eh, no, realment no tenia cap expectativa, però no pel curs en si... no sí si se m’escolta...

**E: T’escolto, t’escolto.**

P: Aleix?

**E: Sí, sí, t’escolto.**

P: Vale, que realment no tenia cap expectativa, però no pel curs en si, perquè la meva forma de ser fa que mai tingui expectativa de les coses. Llavors no sé què em trobaré, no em faig expectatives, però si que he de dir que sí en algun moment he pensat en el curs, realment m’ha aportat molt més del que podia esperar (O.1).

**E: Super.**

P: No sé si era de Borja Vilaseca, “mientras más expectativas te hagas, mayor diferencia entre la realidad de la expectativa y te la pegas...”

1. **E: Sí...molt bé, molt bé, molt bé. Em... què destacaries positivament del curs? Lo que més, li diríem, lo que més...**

P: Eh... la freqüència setmanal, amb el mateix horari (EO.1) y a esto añadiría lo que ya te puse en la memoria, a mí en mi caso, personalmente me habría ido muy bien una segunda vez de forma vez como voluntaria (EO.4) de “mira, hacemos un segundo día porque entendemos que la gente tiene trabajo o lo que sea, pero los que quieran de forma voluntaria podemos meditar todos juntos tal día”. Porque, por ejemplo, el lunes, el martes e incluso el miércoles yo iba muy motivado... (riu), pero el jueves ya me costaba más...

**E: Ja...**

P: Entonces, quizás una segunda sesión durante la semana, ya no digo dirigida por ti, porque ya sería demasiado trabajo, una segunda sesión para mi me hubiera ido muy bien...

**E: Brutal...**

P: ... para acabar de tener el hábito.

1. **E: Ja la penúltima. Com valores aquest tipus de programa pel teu rol professional?**

P: Ah, perfecte. Em sembla perfecte (O.1) perquè és una cosa que també agafo una idea, que comentava abans, ara sóc més conscient de segons quines coses (AG.1) i ara, per exemple, si he de donar alguna xerrada potser començaré a introduir una miqueta el tema aquest del *mindfulness*, no com un curs, però idees, no? Potser per si faig una SSP, la típica SSP que dius “ara l’àrbitre es confon”, no?”, la típica esta de “l’àrbitre sempre pita en contra d’un equip...” per a gestionar...

**E: Si...**

P: Pero a mí ya me sirve para primero, enseñar a los jugadores y sacar el tema de las emociones y (inintel·ligible). Entonces digamos que me viene muy bien por eso, para tener, otra vez, una base científica o no sé cómo decirlo, una base sobre la cual poder justificar porque es importante trabajar las emociones (CT.3).

1. **E: Top, eh... vale, i la última. Quines millores implementaries en el programa, si és que ho faries?**

P. La que t’he dit abans, potser un segon dia... (EO.4).

**E: Sí, perfecte..**

P: És aquesta potser...

1. **E: Sí, sí, és la que anava a posar, si no la deies, ja la pensava copiar. Vale Andreu, i si vols fer un comentari final, algo que no t’hagi preguntat i vulguis dir...**

P: No, també m’ha semblat molt positiu (O.1) el fet de que comparteixis el pdf., perquè hi ha moltes vegades que fas un curs i “Bueno, si has apuntado bien i si no, no...” y el hecho de tener el pdf. yo lo agradezco, el tema de enviar todos los pdf. complementarios, toda la información complementaria... (EO.3) Que sí que es verdad que a veces es mucha información de golpe, pero siempre uno tiene en mente “Bueno, cuando tenga libre voy a acabar de mirarlo o sé dónde mirarlo si quiero leer sobre este tema o si quiero recomendarlo a alguien sé dónde hacerlo”. Ahora por ejemplo, con mi prima que le gusta mucho el tema este y ella está haciendo un poco el mindfulness por libre y le he dicho “mira, he empezado a hacer un curso y si quieres lo hacemos juntos” y ahora como que tengo la intención de hacer con ella lo que has hecho con nosotros durante 6 semanas, pero también es una forma egoísta de obligarme a mi mismo a serlo... y de un punto de motivación de hacerlo con ella y tengo como “Bueno, le voy a enviar un pantallazo de lo que se podría leer..” o cosas así.

**E: Andreu, o sea, mi material es para compartir, tú puedes hacer el uso que tu quieras, me parece una excelente forma de adquirir los conceptos, porque ¿no? cuando uno lo tiene que explicar es cuando realmente se lo prepara, utiliza el material complementario para repartir... porque al final es impactar a cuanta más gente mejor y por eso precisamente os comparto el pdf.**

P: Sí, pero ese pequeño detalle que tienes tu no lo tiene todo el mundo, ¿sabes? Es un pequeño detalle lo que tu estás compartiendo, el conocimiento no solo es una formación de 1h online, sino realmente “mira, te lo doy todo”. Yo al menos lo valoro mucho en el curso, el tener el material, porque muchas veces haces la formación y si te acuerdas... todo el mundo sabe que al cabo de 24 horas te acuerdas solo del 20% (riu).

**E: (riu)**

P: Y dices, bueno, se agradece, es lo que dices tu...”no me acuerdo de nada y quiero mirarlo otra vez, quiero volverlo a leer”

**E: Claro, exacto, por eso estamos Andreu. Oye Andreu, pues muchísimas gracias por confiar en el programa, sabes que te aprecio mucho…**

P: Y yo a ti Aleix...

**E: ...y estoy a disposición siempre que quieras hablar, dudas, más información...porque claro, yo al final también... o sea, de cada vídeo hay 30.000 más que van ligados... también es un trabajo que os dejo a vosotros para que decir “ostia, ese comentario me suena que lo ha dicho ese autor” por eso siempre os digo que tiréis del hilo, porque aquí hay muchos hilos que estirar y un autor te lleva a otro, a otro a otro... y vas expandiendo a medida tus intereses.**

P: Exacto Aleix, totalmente de acuerdo. Yo lo insisto una vez más Aleix, lo valoro que has puesto la piedra, luego ya cada uno va a llegar hasta donde quiera llegar, pero creo que es lo importante... el sembrar una piedra y un empujón y ya...

**E: Una semilla...**

P: Una semilla, exactamente. Yo lo agradezco, es como tienes un grupo de 12 jugadores, lo importante es que aprenda 1 o 2, o sea, el que realmente quiera aprender, el que no quiera aprender es el 50%, no va aprender nada. O sea, no os preocupéis por ese jugador que no quiere aprender... O sea, tu nos has dado un conocimiento y por donde empezar, una base, un camino... nos has enseñado a pescar (EO.1) (riu).

**E: Está claro...**

P: Te lo agradezco Aleix, y sabes que te aprecio mucho y te conozco de no sé, 2015 o por ahí...

**E: Sí, sí, hace tiempo. Un placer Andreu.**

**ENTREVISTA 7. CARLES (Entrenador, 29 años) – Código P.7**

**Realizada *online* el día 7 de abril de 2021**

1. **E: Ja està gravant... vale, Carles, ets entrenador de la Barça Escola a Barcelona i has participat al programa de ‘*Mindfulness* per Entrenadors’ i la primera pregunta és: com diries que el programa t’ha impactat, si és que ho ha fet d’alguna manera?**

P: Vale, doncs, em... breument, jo no havia meditat mai i quasi no sabia el que era el *mindfulness* i res, la veritat que des del... bueno, des del primer moment, la primera sessió que va, fer em va interessar perquè són temes que m’interessen i la veritat és que la cosa que em va impactar, em va agradar moltíssim és que des del primer moment, em... bueno, ens vau dir que era un espai segur, que era un espai per compartir i jo crec que ja, en el meu cas per lo menys, va ser molt guai perquè potser a lo millor potser notava més seguretat o jo que sé... (O.2), però a nivell de curs la veritat és que tocant varis temes, vale? I provant, sobretot provant aquestes meditacions (PE.1) o aquests exercicis també informals en el camp (PE.3) o en qualsevol lloc (PE.2) doncs bueno, el simple “parar” per un moment (PE.2), que a lo millor durant aquesta vida tant europea, no? Tant plena de coses i d’activitats a lo millor no parem o no ens focalitzem en el que fem... (AG.4) doncs, bueno, més o menys això, que em va impactar molt (O.1) i provant-lo sobretot, perquè al final és com aprenem, no? (PE)

**E: En destacaries alguna cosa a nivell personal o professional que t’hagi impactat?**

P: Jo diria que, o sigui, en aquest dies també com que no és que sóc conscient cada moment, perquè això no, però sí que noto que en alguns moments especials del dia o particulars que em paro i dic “què estic fent?” i tal... i abans no ho feia, no apareixia això o a lo millor no em focalitzava i estava aquí, però ja estava pensant que he de fer després o que he de fer demà o com serà la vida en 3 mesos i tal... i ara és com, no sempre eh, perquè seria una mentida, però sí que hi ha alguns moments on em dic, “ostres, m’estic adonant que estic provant a viure això”, sense anar-me cap allà o anar-me cap allà... això es la cosa més guai que m’està passant (AG.1/PE.4)

**E: I a nivell professional, com diries que t’ha impactat?**

P: A nivell professional... jo crec que, primer de tot, m’ha impactat més a nivell personal que a nivell professional. Però sí que a nivell professional... no sé si estic tenint els mateixos resultats que a nivell personal, vale, ho estic intentant, però és com que em costa una mica més... sí que al camp, quan vas dir provar a fer aquest exercici sí que ho vaig provar alguna vegada i sí que va ser guai perquè bueno, quan vaig comentar això del soroll de la pilota cada vegada i tal...

**E: Si...**

P: I en aquest moment en que tenia aquest “anclatge” va ser més fàcil estar allà i estar pendent del que esta passant allà, com en un mon paral·lel... és com estava allà i no hi ha res més que aquest soroll... (PE.3), però sí que sent sincer, m’ha impactat més a nivell personal que professional, en professional encara estic en això i hauré de millorar, però de moment més o menys.

1. **E: Perfecte, eh... has modificat o has incorporat algun comportament o alguna estratègia o algun aprenentatge en el teu... bueno, a nivell personal i a nivell laboral també?**

P: Sí, jo diria el que he incorporat més són les respiracions, aquestes respiracions que encara que no siguin per 15 minuts seguits, són coses que per alguns moments del dia o quan estic corrent d’un lloc a l’altre, com que faig 2 o 3 respiracions d’aquestes diafragmàtiques. I això sí que ho he incorporat perquè no ho feia, i si ho feia no n’era conscient d’això... i això és una cosa que m’estic adonant que estic fent més i més, la respiració (PE.2). A nivell d’altres coses a vegades... bueno, en moments puntuals, vale? No sé, és que a mi el sol a la cara és un altre anclatge que, no sé, quan estic sentat amb un cafè o algo arriba el sol i tanco els ulls i pum...

**E: Et connectes, molt bé...**

P: ... ja no necessito res més i estic content (PE.2). La respiració, si he de dir un, la respiració...

**E: Molt bé...**

P: Res, bueno, respirem sempre, però....

**E: No, pots dir els que vulguis eh... però sí, està bé...**

P: Aquests dos crec que són els més recurrents.

1. **E: Vale. Què diries que ha sigut el més beneficiós per tu del curs?**

P: Més beneficiós... bueno, dos coses jo crec. Una, poder compartir i poder escoltar altres experiències (O.2).

**E: Aha...**

P: Que això crec que sempre és una cosa beneficiosa per tothom si estàs obert a escoltar i aprendre de tothom està molt guai, perquè estem tots... bueno, o la majoria, en el mateix barco, no? RI.3) En aquest curs que compartim, i això esta molt guai, i crec que és molt beneficiós per tothom. És a dir, jo comparteixo, comparteixo i anem sumant tots. (O.2) Bueno, i la segona, que és algo nou, almenys per mi, i que bueno, que he provat i que estic provant poc a poc a adonar-me’n una mica més (PE.1) dels moments o del que estic fent, una mica més de consciència (AG.1)... i això crec que ara al dia sí que estic com agafant aquest benefici, vale? És en moment puntuals, encara no ho faig tot el dia, però sí que en alguns moments me n’adono i sí que dic “ostres, estic millor, no?” O estic més relaxat... (PE.4) llavors això, crec que sí que ha sigut un benefici a nivell psicològic i molt guai (O.1).

1. **E: Molt bé, em... quins han sigut alguns dels reptes o dificultats que t’has trobat, si te’ls has trobat, per completar el programa?**

P: Bueno, dificultats, bueno crec que t’ho vaig compartir al principi, sobretot el meditar (PE.1) que bueno era, i crec que segueixo una mica escèptic en això, no sé perquè... crec que una mica perquè em costa i això. Al principi va ser “ostres, es difícil, no puc o no sé com fer-ho”, saps? Ho provo a fer com em guia l’àudio i tal, però em costa... Sí que amb *anclatges* puntuals doncs sí, va ser mes fàcil, però jo crec que això, vaig tenir una dificultat, però més que res perquè no ho tenia amb els hàbits, no ho coneixia, no ho conec i sí que ho he provat i ara sé més o menys què és, no sé com es fa perquè al final cadascú té la seva, no?

**E (So d’assentiment)**

P: Com tu vas comentar, Aleix... però sí, això va ser una dificultat al principi i jo crec que la resta bastant bé, perquè les sessions molt guai (O.1), un espai per compartir segur, on escoltes gent que ja coneix la practica i la teoria molt més, porta anys, doncs això sempre suma... (O.2), però crec que la dificultat, si n’he de destacar una seria el meditar, el meditar en si que era com algo molt nou i com totes les coses noves, bueno, la majoria coten una mica.

1. **E: Perfecte. En quina mesura el programa ha coincidit amb les teves expectatives inicials, si és que les tenies.**

P: Bueno jo, la veritat, expectatives tenia, bueno, normal en el sentit que la meva parella ella també fa cursos de *mindfulness*, vale? Està en la psicologia i doncs ella m’havia explicat tal i una mica... i doncs m’esperava bastant perquè ella sempre em diu “ostres, has de viure aquí, ahora”, però què significa això? (Rient)

**E (Riu)**

P: Saps?! “¡*Aquí ahora va, va, va, aquí ahora*!” Semblava un camp de futbol i el míster “*Aquí Ahora, aquí ahora*” (Rient)

**E (Riu)**

P: Bueno i, doncs, no almenys ara després del curs sóc una mica més conscient de què és o de com es pot incorporar en la teva vida i en el teu dia a dia i dir-te que jo surto molt content (O.1), perquè a part de tot el coneixement científic, teòric, articles que hi ha darrere... que clarament és algo estudiat, provat i tot, al final si no ho proves no ho saps i el curs m’ha permès provar-ho i sentir-hi (PE) i doncs, bueno jo crec que ha satisfet bastant les meves expectatives (O.1) o idees que tenia del curs.

1. **E: Vale. Perfecte, què destacaries positivament del curs, si haguessis de destacar algo?**

P: Em...a veure...bueno, això que ja t’he comentat de que des del primer moment espai per compartir, espai segur... això crec que ha sigut la clau per mi, per bueno, per... mira, em poso a jugar i jugo, saps? O.2)

**E (So d’assentiment)**

P: Sense por d’exposar-te, perquè al final no hi ha cosa que està bé o malament i això és com “bua tio, això a la realitat no passa...” estem en ambients on ostres, que això no sé si dir-ho perquè a lo millor es pensen que sóc un tonto o és una tonteria... i crec que això va ser una diferència i algo molt, molt guai (O.1)... i dius ostres, això hauria de ser així amb la feina, amb la parella, o jo que sé... (O.2)

**E (So d’assentiment)**

P: Totes aquestes coses...i bueno després, positiu... no sé que dir-te més, això segurament...

1. **E: Està perfecte, està perfecte... Com valores aquest tipus de programa pel teu rol professional?**

P: Bueno, eh... sóc, o sigui estic totalment d’acord que si un tècnic o una persona encarregada d’un grup (RI.1) que es coneix millor a si mateix (PE.4) o, per lo menys, sap gestionar millor aquestes emocions (AG.2), aquestes coses que tenim dins, doncs jo crec que podrà dur a terme una millor gestió de grup humà, no? (RI.1) Que al final com entrenadors som, no? Crec que això sí que pot ajudar a bueno, no a controlar però si a gestionar millor el que fas, com et portes, com actues a nivell corporal en certes ocasions que ens costa per qualsevol tipus de constrenyiment... del resultat, del temps, no, de qualsevol d’aquestes coses... (AG.3) i jo crec que sí, que ens pot ajudar de veritat (O.1). Potser 2 respiracions abans d’anar a entreno que vens d’una dia caòtic i, respires i buf, vale entro en el camp... saps? (PE.2) com, no sé, un intermediari podria ser, algo intermediari per portar una pràctica millor... o una pràctica més gestionada (PE.3).

**E: Què vols dir intermediari, algo intermediari...**

P: No sé, ara amb la imatge d’aquestes 2 o 3 respiracions abans de començar... no sé, vens d’un dia de la oficina i arribes al camp tot com “buf”, és que tinc 5 minuts per preparar el camp, no sé què... i dius, “3 respiracions” i dius, “vale...” com que et fa aquest mediante, a través...

**E: El pas, si...**

P: ... aquest guioncito entre una i l’altra, trobo la part conscient, estic al camp i m’he de focalitzar en això, això i això (AG / PE.4)

1. **E: Vale, si, perfecte, perfecte. Ja la ultima, implementaries alguna millora en el programa?**

P: Em... bueno, això jo crec que t’ho vaig escriure en el *feedback* i tal... algo que potser implementaria, però que potser seria una cosa... no sé si es podria fer o tal... però seria un espai compartit (O.2), que sigui d’una mitja hora o 20 minuts o jo que sé... o un grup, o un jo que sé, vale? Per seguir en aquesta dinàmica (EO.4) de, vale? Jo comparteixo el que estic fent, tu comparteixes el que estàs fent, no per comparar-te perquè comparar-te no és bo i no s’hauria de fer...

**E (So d’assentiment)**

P: Més que res per veure com la gent arriba a aquest punt de consciència, d’atenció plena... i això crec que estaria guai. Sí que hi han mil vídeos, mil persones que expliquen coses però no sé, com que seria una cosa més propera, més de casa, no sé... més espai o zona de confort (O.2), que bueno, seria una mica contraproduent, hem de sortir d’aquesta zona...

**E: No, no sempre...**

P: No, no sempre...bueno, en aquest cas jo crec que estaria bastant còmode com a zona de compartir, compartir sense jutjar... (O.2)

**E: Perfecte, perfecte...**

P: Algo que implementaria o bueno, una cosa extra que si...

1. **E: Si, bueno en realitat estava previst això, o sigui tots els curs comencen amb 5 minuts on per parelles s’explica com ha anat la setmana, que ha fet, quines dificultats s’ha trobat... el que al final és una limitació del *Teams*, perquè amb el Zoom us puc dividir ràpid per parelles si us torno a les ales sense problemes i en canvi amb el *Teams* hem de preparar totes les sales i es una mica més dificultós... però això tots els cursos de *mindfulness* ho tenen. I és algo que volia implementar, però al final veient els problemes tecnològics... però és un molt bon punt. I res Carles, si vols afegir alguna cosa més, algun comentari al final?**

P: No, bueno que, encantat i merci una altra vegada de bueno, tu i a la gent que ha participat en el curs i la creació d’aquesta activitat i res, felicitats i a seguir contagiant gent amb això, no? Que crec que ens fa falta com a població, almenys aquí a Itàlia, Espanya o Europa en general... la vida caòtica amb mil *tareas*, tasques, activitats... no?, no? A seguir i a *tope* perquè és molt útil (PE.5) Si almenys arribés a aquest punt de consciència del que és o el que pot arribar a ser, guai. (O.1)

**E: Super, Carles. Moltes gràcies a tu...**

P: De res...

**E: Ens veiem pel camp, que vagi bé!**

P: Perfecte, adéu, adéu!

**ENTREVISTA 8. POL (Director Academia Internacional, 35 años) – Código P.8**

**Realizada *online* el día 7 de abril de 2021**

1. **E: Tenemos a Pol, director de la academia del Barça en Varsovia y que formaste parte del programa de ‘*Mindfulness* para entrenadores’. La primera pregunta es Pol ¿cómo dirías que el programa te ha impactado, si es que lo ha hecho de alguna manera?**

P: Sí, totalmente (O.1), primero el sentimiento de pertenencia por haberlo compartido con los compañeros y contigo (RI.3), el poder ver otras experiencias, verlas desde primera mano durante les formaciones pues fue para mí un impacto bastante grande… (O.2) porque al final cada uno tiene un poco su práctica, medita..., a nivel formal y informal, pero el poder ver todos los comentarios, además de la información complementaria (EO.3) de cada formación que realizaste tú en este caso, supuso un impacto enorme por ese sentimiento de pertenencia, esta nueva tendencia en que cada día es más potente (CT.5), del *mindfulness*.

**E: Cien por cien… ¿a nivel personal o profesional, destacarías algo?**

P: Sí, a nivel personal, sobre todo por la meditación, la práctica formal (PE.1) he cambiado un poco la rutina, sobre todo los *timings*… he estado probando diferentes *timings* de práctica formal… creo que lo comenté en algunas de las formaciones y me dijiste algo muy interesante… yo lo focalicé más a las tardes, después de lo que es la jornada laboral demás, y tú me contaste que no lo asociamos a estrés, bueno, que no era realmente estrés la palabra pero era como acabar el día y más que para relajarte, para aprender también de vivir del presente, no? Y bueno, he cambiado sobre todo las rutinas (AG.4), focalizado un poquito también en la respiración (PE.2?), me ayudó algunos consejos que diste en las formaciones y algunos comentarios de los compañeros… incluso al conducir (PE.2), que ruta cogemos para avenir a la oficina o al campo… muchas cosas que fueran pequeños detalles que he implementado y me ayudan en ese *Flow State* (PE.3) que comenté en alguna de las formaciones con las muchas entrenadoras y entrenadores que tengo aquí, de estar presente en cada conversación, en cada *feedback* (RI.2), incluso focalizar (AG.1), escuchar el sonido de la pelota, focalizarnos en observar desde un poquito más de afuera al técnico, de su expresión corporal, facial… muchas cosas que antes sí miraba, pero no de forma tan profunda (PE.3)… muy positivo (O.1).

1. **E: Muy bien, eh… vale, perfecto. Esta información en realidad la pondré en la siguiente pregunta… sí, porqué la siguiente pregunta era si has modificado o incorporado alguna estrategia tanto en tu vida personal como profesional, ¿no? Y entiendo que aquí podemos incluir todo lo que estás comentando de lo que has llevado a los entrenadores, de todos esos pequeños detalles, del sonido de la pelota, la expresión corporal de la gente… como lo has ido incluyendo primero en ti, entiendo, y después también intentar transmitir a los entrenadores.**

P: Claro, exacto, eh… un campo que me fascina es la inteligencia emocional y a nivel de comunicación sí que he profundizado durante los últimos años, entonces estas formaciones me sirvieron mucho más que un lenguaje verbal, algo no verbalizado. El observar muchas conductas, comportamientos (AG.1), ya sea de técnico o jugador (PE.3). Este aspecto fue donde más me generó un aspecto muy positivo todo el programa…

1. **E: Em… vale, vamos… ¿qué dirías que ha sido lo más beneficioso del curso para ti?**

P: Mmm… bueno, primero, como te comenté el sentimiento de pertenencia, de que es una realidad, de que esto probablemente es de lo más importante que tenemos en nuestro día a día (RI.3). Y lo segundo, que lo vivencié de forma muy natural, ¿no? Las formaciones eran muy… cada uno se expresaba, cada uno hablaba de su día a día, de sus experiencias… que en Egipto es diferente que en Varsovia, o en Barcelona o en Singapur… entonces esa naturalidad con unos constreñimientos socioculturales brutales, pues para mí fue increíble (O.2). Las disfruté cada lunes (O.1) por eso, porqué me impactó el poder ver como gente medita o realiza la práctica, ya sea formal o informal, en diferentes países, se hablaba de aspectos culturales también y no sé… fue como naturalizar algo que, tú lo sabes bien… años atrás el que meditaba era un poco… no sé si decir *hippy*, pero que estaba un poco “pallá”. Pero ahora todo está mucho más naturalizado y este es el ejemplo…todos los técnicos de Barça Escola lo tienen en su día a día, cada uno en su manera…y para i fue eso, la naturalidad y el nivel cultural también, me parece interesante (O.3).

**E: Si la verdad es que es muy bonito ver tanta gente interesada… que parecía que seriamos 4 locos y al final resuena a la gente y la gente tiene ganas… también nos engancha a todos en un momento de edad, vital… que también lo entendemos más, ¿no? ¿Quizás nos lo dicen hace 5 años y “este loco dónde va, ¿no?”. Pero creo que se ha juntado un grupo muy bonito.**

P: Exacto.

1. **E: ¿Cuáles han sido algunos de los retos y dificultados, si es que has tenido, para completar el programa?**

P: Te lo puse en el registro. Lo que yo hice fue cada semana intenté implementar algo en mi rutina y al final he acabo el programa con muchos aspectos super positivos porque ya los tengo super insaturados en mi día a día y los hago de manera natural digamos (AG.4). Por ejemplo, las cosas que comentamos con el Enric, en la naturaleza, porque aquí en Varsovia tenemos mucha naturaleza, bosques, parques… entonces yo con el perro, o simplemente con mis *Airpods* me pongo un *podcast* o incluso una vez fue sin nada, sin *Airpods*, me iba a caminar y simplemente a ver que me venía la mente, a fluir… En el bosque, que lo tengo al lado de casa que solo era correr e incluso en la última semana lo implementé con la actividad física. Suelo ir a correr o hacer algo de actividad física en el bosque (PE.2), pues como me sentía después, como conocerme (PE.4) mucho en esta faceta de actividad física también, no solo de caminar. Espera que me está llamando algún personaje… vale.

**E: Tranqui, tranqui…**

P: Perdón. Y me pareció super interesante, ¿no? El conocernos también (PE.4), en mi caso anteriormente por ejemplo la actividad física era *Airpods*, música o un podcast super interesante motivacional…y como que no terminaba de respirar…

**E: De ver para adentro, ¿no?**

P: Y ara es como que me fijo más en los colores e los árboles, que ahora estamos en primavera aquí y es muy bonito…cosas muy, muy positivas, en los olores de diferente flora… (PE.2)

**E: Los sentidos…**

P: Realmente te lo agradezco a ti, porque es una pasada de programa el que hemos hecho y esto no lo llegaba antes a profundizar tanto (O.1).

**E: Vale…no, pues me alegro muchísimo escuchar eso eh…porque es ahí no y además todos podemos hacerlo, no sé si es verdad que vives donde vivida P, cerca del bosque… es bonito.**

P: No es la misma casa, pero es que al final aquí en Varsovia en este aspecto es increíble. Es muy difícil que no te cuadre un bosque o un lago al lado… se disfruta también.

1. **E: A nivel de expectativas, ¿a qué medida ha coincidido con tus expectativas iniciales este curso, si es que las tenías?**

P: Me he llevado una grata sorpresa porque yo durante los últimos años he meditado, tengo el *membership* de *HeadSpace*, que es un detalle que me parece destacable porque me permite en el día a día meditar. Soy una persona que ha leído mucho de esta temática, sobre todo de la inteligencia emocional, que está muy relacionada… pero vi que realmente es solo un 1% de todo lo que me queda por aprender y profundizar… y este programa como que me ha abierto los ojos, en buena manera, de una manera positiva para ver que esto solo es un principio, de darme cuenta (PE.4) que hay muchísimas cosas para aprender y por optimizar, sobre todo para optimizar en nuestras rutinas (AG.4) y en nuestras sinergias (RI.3) en la oficina y en el campo (PE.3), sin duda.

1. **E: Super. ¿Qué destacarías positivamente del curso?**

P: Del curso, positivamente, una cosa más bien por encima de las demás o...

**E: SÍ…**

P: Mmm…Sobre todo que, aunque hubo teoría todo estaba bastante relacionado con la práctica (AP). Por ejemplo, podemos hablar de los diferentes tipos de personalidad, emociones… pero siempre había una aplicación práctica, o un comentario de un compañero o tuyo… se ve que dominas mucho el programa, entonces tienes experiencia y nos das muy bueno *tips* (EO.1). Entonces a mí me gustaría resaltar eso, estaba orientado a la práctica (AP) y se agradece, ¿no? Porque la final el cerebro es muy complejo, el ser humano, y está claro que podríamos profundizar, yo en mi caso diría que era bastante práctico el programa.

1. **E: Perfecto. Creo que es la última o la penúltima… si, la penúltima. ¿Como valoras este tipo de programa para tu rol profesional?**

P: Fundamental. Sinceramente, lo hable aquí, incluso en la oficina por eso salió tu nombre i demás, que… a mí me parece que es algo que yo si puedo y es que tengo casi 40 entrenadores y entrenadoras… si puedo, el *mindfulness* tiene que estar en nuestras formaciones, en mi opinión personal. Porque muchas veces como gestionamos una confrontación con un jugador o jugadora (RI.1), cualquier detalle yo creo que este tipo de gestión de emociones (AG.2), de comunicación asertiva … (RI.2) incluso podríamos decir que es más fundamental que el juego en sí, ¿no? El cómo los contenidos del juego que como técnico puede dominar, si no tiene esta base emocional (AG.2) digamos, o de fluir, ese *Flow State* (AG.1), de conocerte (PE.4), puede saber mucho del juego, pero después con el jugador no se transmite de forma óptima (RI.2). Yo como DPL (aclaración – Director de Proyecto Local = Director de Academia Internacional) creo que sería super interesante, creo que lo comenté con Miquel… me parece un tema que sería fundamental e incluso bonito el compartir (O.1). A mí, una anécdota del programa, el meditar todos juntos, con la cámara… me parece increíble. Porque fue como que ahora te veo a ti, te conozco más (RI.4), a Miquel, ver a todos los DPL así… como todos estamos en nuestras islas, en nuestros países... el programa nos ayudó a conectar más con todos (RI.3) y ¿porqué no con nuestros técnicos aquí también? (PE.3)

1. **E: Me encanta, me encanta. Vale, y la última, ¿qué mejora implementarías en el programa, si es que lo harías?**

P: Mejora en si como tal no, pero al final el programa al ser *online* pues digamos que es diferente, pero a mi me sorprendió para bien… tampoco es algo que diga que al ser *online* pierda calidad, pero en persona seria diferente… sería interesante el ver cómo nos sentimos los unos a otros en esa interacción presencial, por poner una… creo que te lo puse en el cuestionario, pero… Mejora en sí, tampoco…

1. **E: Perfecto. Nada Pol, si quieres añadir algo que no te haya preguntado…**

P: No, no…felicitarte (O.1) y que no nos conocimos personalmente…sí que nos conocimos personalmente, puede que no te acuerdes…

**E: ¿Por email?**

P: En Vancouver, yo estaba en Vancouver…

**E: ¡Ah, okay!**

P: Yo estaba de entrenador con Toni A…

**E: ¿En esa formación? Okay, sí, sí, me suena…**

P: Un día solo, pero tenemos una foto con los técnicos y ese día nos conocimos, pero no habíamos interaccionado tanto y…felicitarte, me parece un pedazo de programa y que, no hace falta que te lo diga, pero, que felicidades (O.1).

**E: Sí, sí que hace falta, siempre se agradece… eh Pol, muchísimas gracias por la confianza y por las intervenciones, creo que has aportado muchísimo. Gracias también por el tiempo para la entrevista, los cuestionarios y… y el objetivo que podamos seguir con esto y se creen cosas chulas que creo que ya hay potencial.**

P: Genial, genial, aquí estamos para lo que necesites y gracias de nuevo.

**E: Un abrazo Pol, gracias a ti.**

P: Adéu, adéu.

E**NTREVISTA 9. MANEL (Project Manager, 31 años) – Código P.9**

**Realizada *online* el día 8 de abril de 2021**

**E: Estaria bé que comencessis dient el teu nom i el càrrec que ocupes.**

P: Vale. Doncs, el meu nom és Manel, sóc el *Project Manager* de la Barça Escola, Barça Academy i protector del menor en Barça Academy.

1. **E: Perfecte. Manel, després d’haver participat en el programa, com diries que aquest t’ha impactat, si és que ho ha fet d’alguna manera?**

P: Sí, eh... doncs a mi personalment en el que m’ha impactat és el poder que... o l’empoderament a decidir i saber en quin moment (inintel·ligible) estàs en un moment àlgid de feina o d’estrès o de pulsacions que dius, “ostres, m’estic sobrepassant ara mateix i estic en unes revolucions que no controlo”, i saber parar (AG). El desaprendre per aprendre (CT.2) en aquest sentit ha sigut eh..., doncs jo crec el millor impacte. Detectar quan estàs en situacions de risc o trobes que estàs en situació de risc i necessites respirar i agafar aire i agafar una visió més global (AG).

1. **E: Perfecte. Eh, si entrem en estratègies, Bueno si has modificat o has incorporat algun comportament o alguna estratègia o algun aprenentatge?**

P: Discurs i paraula, tant per escrit com per conversa (RI.2).

**E: Sí.**

P: Encara em passa, però sóc conscient de molts eh..., de moltes vegades, quan parlo (RI.2) en situacions de eh, el típic crec, per exemple o situacions així, eh cada vegada sóc més conscient de no crec, eh, ho vull dir així, ho vull tal i aquest comportament que a vegades tens com per eh, no generar pressió, no generar estrès en una altra persona (RI.1), del rollo que no senti que li estic dient alguna cosa que ha de fer, doncs això eh, la veritat és que és una de les coses que també he canviat molt, he modificat en el llenguatge (RI.2). Hem de fer això, al final és el que hem de fer, és la meva feina.

**E: Ja...**

P: Era com deixar molt marge, bueno, anem a veure, a coses d’opinar, a coses de... i al final és, no, s’ha de fer aquesta tasca anem a fer aquesta tasca, mira, la fem així, així, així. Creieu que hem de fer alguna cosa diferent per fer la tasca? Sí? No? Doncs, vinga, tirem.

**E: Perfecte.**

P: He millorat molt en aquests 2 mesos, no hi ha tant temps d’inversió.

**E: I, a nivell personal?**

P: A nivell personal, a casa m’ha ajudat moltíssim, en el sentit de relació amb la dona, de relació amb la família també (RI.4). De potser no, eh, jo sóc una persona que al final eh... sempre he anat molt directe i és com m’han ensenyat a casa, no? Que s’havia d’anar molt directe i aleshores, eh... a vegades, per com se m’ha ensenyat, ho feia així i ara he entès (PE.4), per veure comportaments familiars, eh? he vist que no, que no totes les persones per molt que et diguin s’ha de ser així o creus que ho has de fer així, ho has de fer així. Sinó que, pues, amb els meus pares sobretot, o amb la meva família, detectar que, ostres, sempre ens hem tractat d’una manera molt taxativa i ara quan t’has adonat que, des d’una banda diferent eh argumentes i ho fas i, com un altre moment, parles més de sentiments, parles més d’experiències viscudes amb la pròpia família i expliques situacions per contextualitzar tot, dones una mica més de volta però la veritat és que el missatge s’entén molt millor (RI.2) i la família està molt més receptiva, per exemple, i aleshores jo pensava que érem una família que, en aquest sentit, érem molt directes i el que m’he trobat és que jo m’he convertit en una persona molt directa en aquest sentit i que, a lo millor entén els missatges, quan l’entren, els entén i sap extreure la informació i a casa, en canvi, doncs ho he de fer amb més calma i amb més carinyo, i ho he de fer amb més tranquil·litat (AG.3). I això m’ha ajudat molt (O.1), també. De igual manera, a nivell professional m’ha ajudat molt a entendre companys. Per exemple, on detectar la seva situació d’estrès també. Quan veig algun moment que estem fent alguna cosa conjunta i veig algun moment d’estrès, avisar el company, de dir, “espera, espera, t’estàs bloquejant, anem a pensar un moment. Què hem de fer, hem de fer això, com ho podem millorar, anem a millorar-ho així” (RI.1). Poso una anècdota, ràpida, que és amb l’Adri fent les llistes de les proves d’accés. “Ostres, Manel, que m’he deixat no sé quins grups o m’he deixat tal, merda tio. Hem de començar de zero, hòstia , puta, que l’he liat, que no sé *quantos*”. “Adri, Adri, espera que jo li vaig posant ordre. On et falta, anem a repassar la llista un per un. Vale, està aquí. Vale Adri, ves, tal. El posem aquí, el posem allà, no hem de començar de nou.” “Hòstia, tio, perquè tens paciència eh que si no... No , no és que tingui paciència és que anem a estudiar primer el que ha passat, no?” I, en aquest sentit, doncs te n’adones que el curs també m’ha anat bé, en comparació amb altres companys que no l’han fet (O.1 / AG.2), per dir, ostres, estic detectant aquestes situacions i estic veient com està el meu company i la sensació que està o l’emoció que té ara mateix, i la tinc de canviar no? en aquest sentit.

1. **E: Molt bé, perfecte. Mmm... què creus que ha sigut lo més beneficiós per tu?**

P: A mi, la veritat és que, avui ho estàvem parlant, ha sigut eh... molt beneficiós per a mi com a persona (O.1). Sobretot per això, perquè jo anava molt directe, anava molt dinàmic, sempre estava fent coses, sempre estava que si al telèfon, que si trucant, que si... i... I a mi m’ha anat molt bé per descobrir que necessitava moments (PE.4), i que no havia de perquè estar... o no, perquè... no has d’estar sempre, diguéssim eh... has d’estar però no... ho sigui, has de trobar el teu moment. I jo he aconseguit trobar els meus moments per dir, “ostres, ara necessito...” el que us deia un dia, no? al migdia, ara necessito entrenar, he de desconnectar, he d’intentar parar perquè vaig a dinar amb la meva dona ara, i després ja seguiré amb la feina (AG). No et preocupis que el que no hagi passat ja... eh... no anirà de que ho solucionis d’aquí a una hora. A no ser que sigui alguna cosa urgent de, ostres, que no arribi a entreno i tal i agafes la trucada i la passes, eh... Un cop estiguis planificant, ja no anirà de que acabis a la una, que acabis a les tres.

**E: Ja. Aquests moments S.T.O.P., que d’alguna manera, No? encara que siguin més llargs... el tallafocs que deies.**

P: Exacte. Sí, sí jo això la veritat és que m’ha anat molt bé i, a més, també per separar tasques (PE.3). De vegades el matí és molt de trucades, molt d’organització, molt de fer el dia a dia o el que vindrà i per les tardes són molt més actives, d’estar pendent del P, de si t’han dit alguna cosa pels entrenaments o tal, que ell només ve per la tarda. I aleshores és com que faig la desconnexió de dir, vale, ja he passat de la meva part de programació, ja he passat de la meva part d’organització, ara per la tarda estem reactius a entrenos, estem reactius al que puguin necessitar els meus companys i... i no et posis a fer unes altres coses perquè sinó no estaràs atent al que has de fer realment.

**E: Correcte. Bueno, gestió del temps, organització de les tasques, no?**

P: Exacte.

1. **E: Quins han sigut, si n’has tingut, eh, alguns dels reptes o dificultats per completar el programa?**

P: Jo, la meva dificultat més eh, o el meu problema més bèstia ha sigut el de poder concentrar-me eh, poder separar, per exemple quan feia les meditacions, em costava moltíssim (PE.1). Si les feia en algun moment de dia que fos o post-feina o durant la feina, en alguna aturada per intentar meditar i ara passo a una següent fase o me’n vaig cap a Ciutat Esportiva abans de marxar de casa i em dedico quinze, vint minuts a mi, en aquell moment era impossible o era molt difícil mantenir la mateixa, el mateix nivell de concentració, com per exemple el que vaig arribar a tenir quan vaig estar a l’Estartit aquesta Setmana Santa que vaig anar a meditar a la platja un matí, tranquil·líssim allà al costat de les roques i vaig poder escoltar fins i tot el xoc de l’aigua a les roques i sentir-lo, tot i estar a quinze o vint metres. Va ser com una sensació de dir, bua! Ara estàs aquí, ara...

**E: Ara estàs, ja... I... Jo vaig estar també per allà, bueno, vaig estar a Ultramort i vaig estar pel Baix Empordà també, prop de l’Estartit. Què t’anava a dir, és important, fora d’entrevista, no? que entenguis que no és necessària la concentració, és a dir, vale?, ara l’objectiu de la meditació no és tenir la ment en blanc, sinó simplement anar regulant aquest pensaments. Saps? I de vegades com més estressat estàs, més necessites meditar, encara que és més difícil baixar els pensaments, no? però per això és aquell moment de poc a poc... i tenir la paciència que pues els primers cinc minuts això va a tope però després això va, va a poc a poc baixant. Això no vol dir que ho estiguis fent malament ni que... saps? Simplement és observar com està funcionant la teva ment en aquell moment.**

P: M’he adonat que això fins i tot eh, en el cotxe (PE.2), a vegades, aprofito i ho faig quan estic de camí cap a ciutat esportiva, cap al Camp Nou, si jo sóc conscient en algun moment que no paren de passar pensaments de la feina i tal, estic en el cotxe que no puc fer res més, que com a molt una trucada i... i a vegades passen aquests pensaments, penso durant cinc, deu minuts coses, m’adono que (PE.4) no estic escoltant la cançó que m’agrada, que no estic escoltant el programa que m’agrada i, ostres, espera, que estàs de camí a la feina, no pots fer res, o sigui, no pots agafar l’ordinador ara.

E: Ja...

P: Pots fer alguna d’aquestes coses que t’estan passant per aquí? No. Torna’t a posar la cançó, escolta-la bé i gaudeix-la.

**E: Molt bé.**

P: Sí, sí.

1. **E: I, en quina mesura ha coincidit la formació amb les teves expectatives inicials si és que les tenies?**

P: La veritat és que ha sigut més gran la..., al menys en la sensació, és que ha sigut molt més gran el que heu ofert a la formació que no el que jo m’esperava (O.1). O sigui, les expectatives eren molt més baixes, en aquest sentit.

**E: Okay, o sigui les ha superat, oi?**

P: Sí, sí, jo per mi les expectatives eren, doncs, que, sincerament eh, pels cursos que hem anat rebent i que hem anat fent, pensava que seria algo molt introductori, que estaríem parlant de a lo millor com treballar a la feina o, fins i tot, com anar al propi *mindfulness* i a la meditació, no? No teníem, jo crec que no teníem gaire clar exactament com aniria la formació en aquest cas. I la veritat és que el curs ha sigut, en aquest cas, bueno, jo, la meva sensació eh, ha anat per sobre de les expectatives, però perquè s’ha donat molta informació també perquè nosaltres puguem anar recabant aquesta, aquestes petites píndoles que potser ens falten o que potser ens cridin més l’atenció i cada sessió anava amb material complementari (EO.3) que és, ostres, aquesta sessió m’interessa una mica més, tinc el material aquí per fer-ho.

**E: Valores, valores el material complementari, eh?**

P: Molt, i el *mail* del dijous el valoro molt també (EO.1).

**E: També, no?**

P: Sí. sí, sí. Donava mitja vida eh això com un “no t’oblidis”...

**E: Pam, pam, no?**

P: Sí, sí.

1. **E: Vale, perfecte. Eh, eh, què destacaries positivament del curs? Què valoraries com lo millor potser diríem?**

P: Jo crec eh , aquí tinc dos coses i tindria dubtes en el que més. Per una banda la...

**E: Pots dir els dos, sí, digues.**

P: Doncs per una banda l’aconseguiment de que els companys, com per exemple l’experiència que vaig tenir amb un dels companys amb el que ens havíem de compartir sense mirar (RI.2 – aclariment: pràctica en parelles a la sessió de comunicació conscient) EO.2), l’experiència que els companys diguin, ostres, gràcies a aquestes coses se n’adonin que... que tot és molt més fàcil, que al final hem d’expressar el que sentim i no ens ha de donar por (O.2), dons, jo aquí no sé si... no sé si ho vaig fer a posta o no, però vaig jugar-la quan vas dir “hem de posar una situació crítica”. Jo crec que quan el meu company em va explicar, tu ja et vas adonar cap a on vaig anar, que li vaig explicar i va dir algo així com que no sentia que el que havia donat ell d’informació tenia ni la meitat del valor que jo havia donat en aquest (inintel·ligible).

**E: Ja**

P: I... i va se perquè, evidentment, vas dir una situació crítica i jo vaig anar a explicar una situació crítica i va ser un dels motius que a mi em va fer començar el curs, que va ser lo de *l’enfermetat* de la meva tieta, no? I... i li vaig explicar directe sense *tapujos* tal i com ho vam fer una mica amb la prova aquella amb el Sisco, quan vam parlar de tot.

**E: Sí.**

P: I aleshores vaig entrar allà i el company es va quedar, “ostres tio, jo t’anava a explicar una situació de feina que no té ni la meitat de valor”, no? I jo crec que aquí és quan... quan se n’adonen que aquest curs és per això mateix, no? per obrir-nos i per dir... o un dels motius que almenys jo entenc és obre’t, digues el que sents perquè així podràs aconseguir allò que vols o podràs aconseguir allò que et proposes, no? (O.2)

**E: (so d’assentiment). Fantàstic. I la segona?**

P: I la segona és el... la cohesió de grup que crec que hem fet. O sigui, jo crec que aquí el mateix grup, a mi per exemple, treballar amb el Dani (inintel·ligible), Xavi, amb el Miquel, no? que estem a oficines però a vegades doncs compartim tant, veure aquesta part més personal, aquesta part més emergent de... de les pròpies emocions crec que a tots ens ha anat bé per saber quin grup som i aquí sí que crec que ho heu fet molt bé quan per exemple vau dir, “ostres, potser us aniria millor en aquest grup que esteu en oficines o tal i a mi m’ha ajudat crec que a conèixer i a treballar una mica més amb els companys, de millor manera (RI.3).

**E: Buaa, Bueno, aquí m’has donat una idea que és interesant, no? de crear els grups de treball, no? No només interrelacionar gent de tantes àrees sinó dir escolta per exemple el grup de recursos humans, no? que és algo que els hi proposaré eh, dir no, pues l’equip de... recursos humans, o l’equip de... l’escola, que en realitat potser es podria plantejar de començar amb recursos humans amb vosaltres, ho estava pensat. Potser també ho podries comentar amb el Carles o amb recursos humans de fer la prova pilot amb vosaltres. És a dir Rosa, Iñaki, i de dir escolta tenim una hora i quart els dilluns com a grup que entrem en aquest nivell.**

P: Jo crec que això seria una de les coses brutals perquè... perquè ho comparteixo i crec que encara estem molt distants. Jo una de les coses que faig en la formació de persones que ens toca cada any i crec que, tu has treballat amb nosaltres en equip i crec que la part financera, en aquest sentit, amb la part esportiva, amb la part de gestió, per exemple, estem alineats, ens portem bé, perquè al final som un equip i portem molts anys treballant junts i... i, al final, les coses surten, però hi ha molta distància, després. Arriben punts on jo crec que aquests cursos anirien molt bé perquè, doncs aquesta Rosa per exemple entengui les situacions del Pau, el Pau entengui les situacions de Rosa i que de vegades no es generi estrès com, “ostres, és que no vol fer això per mi, o no vol fer això per ajudar-me”...

**E: Clar.**

P: Que vegin que com a persona no hi ha cap problema, sinó que a vegades simplement és que el treball et fa poder fer coses i ens fa no poder fer segons quines coses en certs moments, no?

**E: (so d’assentiment) Brutal.**

P: I sí que és veritat que a vegades veig distància entre companys perquè tenim com gustos o idees molt diferenciades i podria... i crec que estaria molt bé. Jo, al menys, eh he notat aquesta, aquest apropament amb certs companys (RI).

1. **E: Molt bé. Vale, i l’última, Bueno, la penúltima, com valores aquest tipus de programa per al teu rol professional?**

P: Molt bé. Jo el valoro molt positivament (O.1), però perquè crec que vosaltres teniu moltes situacions d’estrès o moltes situacions on... Et torno a posar una anècdota eh, aquestes dues o tres últimes setmanes van ser, hem de preparar proves per 300 i pico xavals, 400 i pico xavals que s’ha de trobar cada llista d’espera l’any passat. A la vegada estava preparant un anunci de Veri, eh la competició, la respectiva roba, parlar amb àrbitres i aquestes coses és, gestió de Joanma pura i dura. No? Eh... a més estava cobrint baixa del Pere, que estava de paternitat i vaig agafar la seva roba, l’Anna i la Rosa, la Rosa havia agafat vacances...

**E: Fent vint mil coses, eh?**

P: Vint mil coses. I... i amb aquest sentit, a mi m’ha ajudat a dir, “ostres, tens massa coses. Organitza’t, eh, posa’t bé les coses, organitza’t una agenda eh. Dedica cert temps a cobrir les parts de la (inintel·ligible), dedica cert temps, avisa i amb, amb els meus jefes, amb l’Iñaki, agafo això, però pensa que tinc totes aquestes coses. Potser no arribo en algun moment, eh?”. Puc deixar-ho estar si és algo no essencial i puc detectar i puc determinar quina cosa faig després, faig i eh... sí, sí, sense problema. Aquesta comunicació assertiva (RI.2), no? ha sigut com veure que tu no podies, no et donava temps, no? I, perquè m’he de preocupar, perquè he de començar a fer més hores, si és que són coses que no controlo i que han passat en el departament i que estem reorganitzats, doncs, escolta aixeca la mà i digues-ho, no? I això en el meu àmbit professional m’ha ajudat moltíssim (O.1) perquè tens mil trucades, mil coses i és com vale, ara toca proves, anem a proves, deixem la resta. Els hi dius i fins i tot a la gent, escolta estic de proves, tio, demà et contesto, demà pilotes, demà et contesto tal.

1. **E: Brutal. Eh, perfecte, i... Quines millores implementaries al programa si és que ho faries?**

P Buaaa... Aquí la veritat és que parteixo del desconeixement de com heu organitzat el programa o com està estipulat. Jo crec que, com a molt, eh les millores que... que oferiria és, com ens va passar un dia, deixar més espais oberts (EO.2). Potser organitzar cada quinze dies alguna sessió que no tingués eh contingut com a tal, formatiu, sinó que fos més de compartir perquè a vegades ens passava, no? que quan arribàvem a detectar segons quines coses, com el dia de les emocions o tal, que aquest espai d’haver fet la informació i que s’acabés l’hora era com ohh, se m’ha fet curt! (EO.4)

**E: Ja...**

P: ... moment de compartir i de veure què han sentit la resta i què han tingut (O.2).

1. **E: Vale, més temps, no? Espais oberts, més temps. I vale, Manel, ja està. Si vols afegir algun comentari final.**

P: Jo, la veritat és que com a comentari final ja us ho he dit i us ho tornaré a dir, estic encantat. Aleshores, eh agrair-vos la feina perquè a mi, personalment, eh m’ha ajudat molt. Sí. Jo estic molt content d’haver fet aquesta, aquest curs i ja et dic que no pensava que fos en aquest nivell (O.1). I, potser, sembla com, “ostres, m’està alabant o m’està dient...” No, és que han sigut 6 setmanes en aquest sentit, o 7 setmanes que molta de la informació segurament ja la teníem apresa i ja la teníem dins i sabíem que existia i sabíem com, no? pues això que hem parlat, el Sergio Fernández, el Esteban (inintel·ligible) o tal. Ho tenia clar. Però són coses que no fas a lo millor fins que no et paren els peus o no et posen la consciència a nivell de terra i et diuen, tio, pensa-hi, en això. Eh, situa’t i mira a veure què t’està produint, no? Escolta’t, i aquesta part d’escoltar-te (PE.4) és brutal (O.1). Jo, per exemple, en aquest sentit a l’estiu de director va haver un moment d’estrès molt *heavy* de 32 o 33 dies sense parar cap dia perquè, encara que fos dos hores, havia d’anar a Barcelona per penyes o per alguna cosa i no vaig tenir descans en 32 o 33 dies. Va ser una de les vegades que van haver d’operar el meu avi, que jo tinc una relació brutal amb el meu avi, i va ser allà on vaig dir, ei, això ho has de parar i va ser quan un dels motius i li vaig dir al Pep, “tio, sóc el primer director que ha estat sol a Barcelona. No pot ser i no pot ser” i vaig posar el fre, per exemple. Ho vaig fer involuntàriament o inconscientment, en aquell moment. Però ara quan, quan prenc consciència d’aquell moment o recordo aquell moment, és el teu cos t’estava demanat aturada perquè et trobaves malament i al final, la teva part conscient va dir, tio, digues-ho perquè és que no pots seguir així. No aguantaràs.

**E: Ja, Ja. Manel, moltes gràcies.**

**ENTREVISTA 10. TONI (Departamento de Metodología, 34 años) – Código P.10**

**Realizada *online* el día 12 de abril de 2021**

**E: Serà res, 20 minuts, mitja horeta, també depenent de lo que tu et vulguis esplaiar. La idea és contribuir en l’anàlisi del programa, de forma més a nivell qualitatiu, no hi ha respostes correctes o incorrectes, és simplement conèixer la teva experiència. Vale?**

P: Perfecte.

1. **E: Per confirmar, Toni, coordinador a nivell metodològic d’Àsia Pacífic i has format part del programa de ‘*Mindfulness* per entrenadors’. La primera pregunta és com diries que el programa t’ha impactat, si és que ho ha fet d’alguna manera?**

P: Hm... doncs em... el més destacat, el que he sentit un gran impacte ha sigut en aquesta capacitat d’atenció (AG.1), no? Sobre com gestionem el dia a dia (AG.4), les emocions (AG.2), sobre les entrades en pilot automàtic (PE.4)... ja no només a nivell mental, sinó a nivell fisiològic... no sé si fisiològic, però de tot el cos com ens afecta, i m’ha donat estratègies per gestionar-ho (AG.5). Llavors que sí, molt positiu (O.1).

**E: Destacaries algo a nivell professional o a nivell personal?**

P. Destacaria algo a nivell professional o personal?

1. **E: Bueno tranquil, fem una cosa, anem a la segona pregunta que em sembla que anirà una mica per aquí, perquè anava una mica per les estratègies. Llavors la pregunta és si has modificat o has incorporat alguna estratègia, algun comportament o algun aprenentatge, tant en la vida personal com en la laboral?**

P: Doncs he ampliat els moments de connexió (AG.1) a nivell personal i a nivell professional també... crec que vaig deixar constància al llibret, inclòs treballant ha sigut curiós veure com moments de contacte o ancoratges esdevenien el tacte del teclat o el tacte del ratolí (PE.3). Aquell moment suau, que estava fent el *scroll up* o el *scroll down*... la sensació aquella que mai l’havia percebut com a tal. Després, a nivell de estratègies sí que és veritat que he incorporat lo dels blocs i és una d’aquestes coses que crec que tindrà un gran impacte d’aquí en endavant, perquè sí que ho he introduït i he notat el tast per la bona sensació, però encara no he sigut constant en l’hàbit. I crec que ha sigut una gran incorporació (PE.3) sobretot perquè, quan feia el bloc, al aixecar-me, no? Això sí que ho tenia, quan acaba el bloc, m’aixecava canviava de sala, d’ubicació i... i era molt gratificant notar el tacte taló punta del peu quan caminava (PE.2)...

**E: Okay...**

P: I sí, crec que seria el més significatiu del que incorporaria a pràctica, al marge de la meditació que ja la feia, sinó que incorporaria el que vam comentar un dia a primera hora.... perquè els dies que ja començava de bon matí amb la pràctica, els moments d’identificació i de connexió s’ampliaven al llarg del dia (PE.1).

1. **E: Molt bé. A veure, que és el que diries més beneficiós ha sigut per tu?**

P: Aquesta pregunta crec que la vam respondre, no sé si era una de les preguntes dels qüestionaris. I sincerament Aleix, se’m feia molt complicat seleccionar només una cosa, perquè ja et dic des de com m’ha ajudat al fet de mantenir aquesta rutina (AG.4) del programa, ja no només la pràctica del dia a dia, aquestes interaccions del dia a dia (PE.2) accentuava molt la meva atenció (AG.1) sobre això. Llavors, per una banda i havia això, després les estratègies que s’han compartit (AG.5)... com és una cosa que m’agrada, la quantitat d’informació que ens has proporcionat i ens has donat accés es un ventall molt ampli i que he invertit bastant de temps mirant vídeos i saltant articles (EO.3) que per tant m’ha obert tot un ventall que desconeixia (CT.1), no? Començant pel Jon Kabat Zinn, que ara dic “mare meva”, com pot ser que no hi hagués arribat abans?

**E: Jo igual, sí, sí...**

P: Llavors, em costa molt seleccionar una... però trobo que sí que em puc mullar. Per mi la més determinant és generar aquells entorns on la gent s’exposava sense por; al contrari, exposaven les seves pors. I el fet de generar aquests entorns on la gent això em sembla brutal, em sembla el *game changer*... (O.2) recordo que vaig sentor el *TEDTalk* d’una noia que era música al carrer, a Santa Bàrbara, i arrel de tot al carrer es va fer un renom i la invitaven a casa dels famosos de *Hollywood*, Santa Bàrbara... a tocar. I dius, de si d’algo m’ha servit, de que a la que t’assentes davant de qui sigui i el reconeixement que sigui, obres la motxilla i treus les pors i treus tot el que tenim allò, som iguals...tots anem carregats (O.2). I crec que en aquest programa de *mindfulness* s’ha aconseguit i. per això. per mi sí que és molt determinant en un grup de persones (RI.4).

**E: És molt *heavy* perquè jo no me n’havia adonat tant i he fet 8 entrevistes, i les 8 persones m’han dit el mateix pràcticament, eh... molt *heavy,* eh, que el que més valoraven eren les interaccions, el deixar el càrrec fora, l’espai segur... potser han trobat paraules diferents, però és això.**

P: Sí, sí...

**E: Sí, sí... perfecte, ens marca una mica la línia a seguir, aquest tracte personal...**

1. **E: Eh, quins han sigut alguns dels reptes o dificultats, si és que n’hi ha hagut, per completar el programa?**

P: Per completar el programa, et refereixes al que ha sigut la teva implementació o jo personalment duent-lo a terme?

**E: Tot, bueno... la meva gestió podria haver sigut una dificultat per tu, l’entrenament setmanal... qualsevol cosa que a tu t’hagi suposat un repte o dificultat per completar el programa, si és que les hi ha hagut, eh?**

P: No crec que hi hagi hagut grans dificultats, sinó part del procés com et deia. He dut a terme el programa, però tal com et vaig dir l’he tornat a iniciar ara per fer un 2.0, no? I en tot això que s’ha compartit, aprofundint en els documents, els enllaços i tota l’experiència, donar-li una volta més... aquest tipus d’espiral. No et diria que és el que m’ha costat, sinó el que m’agradaria... clar, m’agradaria que en lloc d’aquesta interacció setmanal fos algo més habitual, que hi pogués haver un acompanyament amb més periodicitat, en petit grup (EO.2), en individual, no? Demanar, demanar clar.... considero que això accentuaria o acceleraria aquest procés (EO.4).

1. **E: Sí, sí, està clar. A nivell d’expectatives, si és que en tenies, en quina mesura ha coincidit el programa amb les teves expectatives?**

P: Mm... és que estava pensant entre o ha arribat o les ha superat (O.1), no? Segurament. Per les dos vessants que t’he comentat abans, una per coneixement (CT), simplement no només compartir des de la experiència personal, es veu que darrere hi ha una recerca. Has anat donant, s’han anat compartint tastets perquè cadascú si pugui indagar, de bones referències (CT) i paral·lelament s’ha pogut generar aquesta dinàmica que et deia que per a mi ha estat el que ha marcat la diferència, no? De que la gent es pugui obrir, generar aquest clima de confiança (O.2), o sigui que sí... no és que em sorprengui perquè pensés que no éreu capaços d’aconseguir-ho, però molta satisfacció (O.1).

1. **E: Molt bé, me n’alegro. Bueno, és que aquesta és molt semblant, no? La pregunta és: què destacaries positivament del curs? Potser anem a lo mateix, no? Si vols reafirmar...**

P: Si, reafirmaria el que ja t’he dit...

**E: Aquest espai, sí...?**

P: Aquest espai (O.2), i aquest vincle (RI.3) eh, és que en el grup dinàmic és molt bo... la de l’escolta activa em va semblar brutal (RI.2), és a dir... a veure si trobo les paraules eh, Aleix... si tenim pressa i hem d’acabar m’ho dius, eh?!

**E: Què va, què va, tranquil.**

P: Era, és a dir, la gent era honesta sense demanar que ho fos, no? (O.2)

**E (So d’assentiment)**

P: I està molt vinculat a lo d’abans, com generar des del “jo m’obro”, de que això és natural, són reptes del dia a dia... ni que jo ho senti com un repte té perquè ser negatiu, aquesta associació entre com ho sentim i el que pensem de com hauria de ser. I des de naturalitzar això penso que s’ha generat un clima de seguretat i honestedat que la gent s’hi ha llançat (O.2). Així que si, reforçar el mateix.

**E: Bueno, però has dit, no, aquest clima de seguretat i honestedat. I entre nosaltres, a veure, honestament... hi havia previstes més activitats d’aquestes, en realitat cada dia hauria d’incorporar una activitat però hi hagut limitacions de temps i del *Teams*, perquè amb el *Zoom* jo faig sales de 2-3 persones i retorno la gent a la sala a classe amb un minut. Aquí per generar parelles, s’havien de generar no sé quantes sales, la gent havia de marxar i tornar, com que el programa *Teams* no tenia aquesta flexibilitat, no? Però la idea, si això continua, és aprofundir molt més en aquestes dinàmiques perquè allà l’experiència és diferent, no?**

1. **I ja per acabar, com valores aquest programa pel teu rol professional?**

P: Molt positiu, molt positiu (O.1). Bueno, és que al final és un recurs, una dinàmica de creixement personal (PE.4), i al final fent la feina que fem, que no deixa de ser interacció entre persones (RI.1), aporta moltíssim (O.1). Des d’estar... afavorir aquest discurs, aquesta conversa interna que tots tenim i el fet de... (almenys en el meu cas, no vull generalitzar perquè no estic segur de que sigui així en tots els casos), però el fet de que tu puguis tenir una conversa molt més sana, compassiva, d’acceptació amb tu mateix (AG), a la vegada afecta tots els que tens al voltant (RI), no? El context del voltant segurament és el mateix, però quan ‘canvies tu, canvia tot el voltant’. Per tant em sembla determinant pel rol que tinc (CT.4).

**E: Bueno aquesta és la que he compartit avui a la sessió 1, res canvia, jo canvio, tot canvia... i...**

P: És que just ara m’ha vingut aquesta frase que ahir estava escoltant el de la mirada...

**E: Ah, el de l’Àlex Rovira...**

P: Sí...

**E: Es passa l’Àlex eh, és molt top.**

P: Aha...

**E: A partir de l’P hi ha un fil per estirar quasi inacabable. Referències, autors, històries... em sembla una obra mestra.**

P: Uf...sí, sí.

1. **E: Ja la última. Quines millores implementaries en el programa, si és que ho faries.**

P: Doncs mira, és just la mateixa que has dit tu abans i crec que ho vaig posar al *feedback*. És el tema d’aquestes dinàmiques (EO.2) que deies, que tenia molt la limitació de la eina i la limitació social... l’altre dia que vam fer la pràctica al camp, no, dijous?

**E: Si...**

P: Hi havia gent que compartia amb el grup gran, n’hi havia que compartien amb el company del costat... i es contagiava, i a la vegada es detectava i cadascú tenia la llibertat per interaccionar (EO.2). Llavors, estic convençut de que si no hi haguessin aquestes limitacions l’experiència hagués sigut encara molt més rica.

1. **E: Sí, està clar, però el format *online* ens permet tenir tots els DPLs connectats; format presencial ens permet una interacció diferent. Pros i contres. Vale Toni, si vols afegir algun comentari final, algo que no...**

P: Crec que... no, crec que ho he posat tot sobre la taula. Ha sigut una bona remoguda i necessària, jo diria (O.1), o el repte al final no ha sigut tant el programa sinó... jo, durant l’escala temporal que he estat fent el programa, així en confiança t’ho dic, just al setmana abans del programa, una situació molt complicada de volum, de gestió, de com jo estava en aquesta retroalimentació de canvi automàtic i tenia unes sensacions que no havia tingut fins a dia d’avui. I ha sigut fer el programa... i de cara al final del programa a també vaig tenir un problema per un tema de gestió del calendari i també hi ha eines que s’han compartit (AG.5) per això... o sigui, tal i com et vaig compartir, molt bona remoguda, amb moltes ganes d’haver iniciat aquest procés i segurament activaré algunes cosetes més.

**E: Molt bé Toni, doncs moltes gràcies personalment i res, ja veurem com aconseguim entre tots que això tiri endavant.**

P: Molt bé Aleix, moltes gràcies.

**E: Una abraçada**

P: Vinga, una abraçada.

**E: Adéu guapo.**

**ENTREVISTA 11. QUIM (Project Manager, 29 años) – Código P.11**

**Realitzada el dia 12 d’abril del 2021**

**E: Quim, vas participar en el programa de ‘*Mindfulness* per a entrenadors’. Concretament quina és la teva posició al club?**

P: La meva posició és *Project Executive* de la Barça Academy a Amèrica. Estic treballant als Estats Units, a l’oficina de Nova York i jo superviso les acadèmies de Nova York, Miami i Brasil.

1. **E: Molt bé. Quim, com diries que el programa t’ha impactat, si és que ho ha fet d’alguna manera?**

P: Sí, eh. Precisament era una de les qüestions que comentava amb el Bruno aquest matí. Jo no he vist un resultat, així com d’altres companys segurament i així amb els que he anat comentant. Jo no he vist un resultat en la qüestió de la meditació en sí, perquè és una pràctica que jo ja portava un any i poc realitzant. I en aquest sentit, doncs, bueno, l’he mantingut i això, no he vist canvis significatius pel que fa al meu dia a dia. Eh, sí que, doncs, m’ha reforçat encara més (AG.4), eh, la... aquesta pràctica (PE.1), no? És a dir, doncs, el fet de no abandonar-la i fins i tot potenciar-la encara més, eh, m’ha ajudat sobretot a partir dels debats que es generaven en les sessions (EO.2) a..., a també a obrir debats interns (CT.2), no? i a coneixer-me una mica més (PE.4). I també doncs buscar reconèixer-me una mica més, és a dir, jo crec que m’ha servit com un incentiu per continuar aquest desenvolupament aportant-me diferents punts de vista. Eh i també m’ha fet veure, doncs, la normalitat de tot plegat. I una cosa que m’ha agradat molt, no? del projecte en sí és el fet de poder parlar amb gent amb qui no tinc confiança eh, sobre doncs, això la... el *self-awareness*, la meditació en general i qüestions així que, de vegades a dins de la societat en la que estem, són una mica tabú i que no acostumem a parlar-les a no ser que sigui amb gent que siguin d’un cercle proper, no? El fet de poder-ho fer amb altra gent, doncs, m’ha fet sentir molt bé (O.2) i m’ha incentivat doncs a..., això, a continuar coneixent-me (PE.4) i a poder continuar coneixent el propi *mindfulness*, a més a més de doncs tota la part teòrica, no? Els fonament teòrics i científics (CT.3) que has anat aportant tu, Aleix, al llarg d’aquestes pràctiques doncs no només m’han ajudat a mi a ser encara més convençut, que ja ho estava, no em feia falta, però sobretot de... ja no por, però de vegades respecte a l’hora de poder parlar amb altra gent, no? (O.2) I doncs poder estendre aquesta pràctica perquè crec que al final, quan més estesa més ens en beneficiarem els que ja la seguim, no? I sóc, bueno penso... d’aquest parer, no? El fet de tenir aquests arguments també, ja més pragmàtics, més, més pràctics ajuden, doncs, això, a gent que potser té més resistències a incloure-les i que així tots creixem més, no? al final.

1. **E: (so d’assentiment) Cent per cent. Eh, has modificat o has incorporat algun comportament o alguna estratègia tant a nivell personal com professional?**

P: Sí, sí que he incorporat el, el, el fet de, de tant en tant proposar-me fer una activitat o acció del meu dia a dia eh... fer-la conscientment. O sigui, a vegades em poso a la dutxa i dic “vaig a fer una dutxa conscient”; sentir les gotes, la temperatura de l’aigua, el fet de si tinc la finestra oberta o no, si entra aire o no, no? I amb el menjar no és algo que hagi incorporat que faci cada dia, però si que intento buscar cada dia o pràcticament cada dia, a banda de la meditació que com deia ja l’acostumava a fer, sí que he intentat buscar aquest, aquesta altra via, no? De fer alguna cosa conscientment i això fer, com ja havies anat proposat tu fer alguna de les coses que són molt, molt automàtiques i que sempre les fem amb el pilot, doncs això, poder fer-la de forma més conscient i això sí que es algo que m’ha servit i he incorporat (PE.2).

**E. I a nivell laboral i professional?**

P. A nivell laboral , eh... sí que, malgrat que no és una cosa que comentéssim directament, sí que hi havia una cosa que potser havia deixat de banda que era que... o sigui, jo abans intentava bloquejar-me una mica les hores en el calendari i una mica havia perdut; sí que en bloquejava algunes, les més importants i tal i les altres les deixava molt obertes. I ara, sense tampoc no bloquejar-m’ho tot, si que he deixat algunes hores que estan bloquejades però que estan obertes dins de la feina, la resta si que ho he intentat doncs tenir-ho tot més o menys tancat. Això em permet saber... i normalment ja intento donar-me un temps de marge, és a dir, si jo sé que fent una tasca trigaré 40 minuts, me la poso de 1 hora, perquè així sé que no tinc l’estrès de fer-ho en 40 minuts, també tinc la calma de que tinc de sobres i doncs arrel de les converses que havíem tingut m’ajuda en la meva part creativa (PE.3), perquè per una banda sé que m’he de dedicar a un tema i, per tant, em permet ser creatiu en aquest tema perquè sé que em sobra el temps, per tant no tinc cap pressa. A més a més, com en aquell dia m’he establert el que he de fer, jo a l’hora de fer aquest establiment, jo ja sé que si compleixo amb aquests objectius del dia, al final del dia me’n podré anar tranquil a casa i oblidar-me perquè jo ja hauré complert amb les coses més urgents i a vegades fins i tot dius “ostia, això doncs això no era ni urgent i ho he aconseguit fer”. De manera que clar, sempre em passava el meu *To Do List*, sempre era immens, mai me l’acabava i sé que mai me l’acabaré, no? Perquè clar, si no em marcava les meves prioritats acabava marxant al final del dia pensant “ostia, encara em queden no sé quantes coses del *To Do List*”. I en canvi, el fet d’això, de bloquejar-m’ho, d’aquesta manera fent-les prioritàries i fen-t’ho d’una manera que no em provoco un auto estrès, em fa anar molt més tranquil en el meu dia a dia (AG.4), em permet dins d’aquests bloquejos... a més a més, sóc conscient del que faig (AG.1) perquè em trec el *e-mail*, em trec les notificacions de les altres coses... Llavors, en aquell temps que tinc bloquejat només faig allò pel que m’havia marcat. Per tant, si m’entra un *e-mail* jo ja tinc bloquejat a primera hora un pels e-mails i els missatges i a ultima hora una altra pels *e-mails* i els missatges. De manera que ja se que, bueno, m’ha entrat algo, ho veure el final del dia... o sinó, si ha entrat abans ho hauré vist a l’inici i ja m’hauré quedat tranquil. De manera que això, em centraré només en allò, estaré 100 per cent centrat i concentrat en allò i conscient del que estic fent no? (PE.3) No aniré fent-ho mentre vaig mirant els missatges o *e-mail*, perquè al final no paren de entrar. I si, acabes fent la feina però no la fas de la mateixa manera, no intervé la mateixa creativitat, el mateix focus. El fet de poder-me concentrar en aquest període sense generar-me aquest estrès i marxar a casa saber que jo he fet el que havia de fer en aquell dia em fa marxar molt més tranquil i per tant, quan estic amb el meu fill després no haver d’estar pensant “ostia, es que demà haig de fer això, demà he de fer lo altre, demà tal”. Perquè normalment ja m’he marcat el que he de fer el dia següent, per tant ara no m’haig de preocupar (AG.4 / PE.3)

**E. Molt bé, molt bé. Brutal, blocs de temps, no?**

P. Sí.

1. **E. Què és lo que ha sigut més beneficiós per tu?**

P. Et diria sobretot el incentiu de... de tornar-me a plantejar coses referents a la meva consciència, no? Del dia a dia... perquè això, com deia abans, eh... doncs per diferents circumstàncies vaig començar el procés de meditació fa un any i pico, no? Llavors en aquell moment era una novetat, llavors llegeixes molt, t’informes molt, xerres amb un, xerres amb l’altre, vas provant diferents coses... fins que trobes més o menys el que et va bé i que et va encaixant en el teu dia a dia i passa a ser una rutina per després acabar sent un hàbit i una mica estava estancat en això i bueno, una mica, vas fent i el fet d’agafar aquest programa, doncs, eh malgrat que hi hagués coses que no fossin noves però que sí que és un tornem a plantejar-nos això i com poder-ho portar una mica més enllà i coses que, això, no?, el que deia abans doncs encara hi ha moltes coses que en el dia a dia faig amb pilot automàtic, no? Anem a intentar aquestes coses que sé que les faig així, a fer-les d’una altra manera. O el mateix que comentava, no? de ser conscient, ja no de vegades de coses que fem amb pilot automàtic, perquè per exemple amb el Dominic, amb el meu fill, és impossible fer pilot automàtic perquè al final canvia cada dia, però sí que hi havia vegades que feia coses amb ell sense estar allà amb ell, no? (PE.4) O, per exemple, perquè, hòstia, la feina i tal i just estic amb ell quan arribo de la feina perquè sé que tinc una hora i poc per estar amb ell perquè després se’n va a dormir i clar hi havia un moment que m’enganxava, no? I el fet aquest, doncs, com puc fer que això, que aquesta tasca que jo faig amb ell la faci conscient, no? I a partir d’aquí jo vaig anar tirant enrere i vale, doncs m’he de marcar un horari de sortida de la feina. Vale, però per marcar-me un horari de sortida he de saber segur que hagi pogut fer tot el que jo havia de fer aquell dia. I vale, com puc fer això? El dia anterior o a primera hora mirar-me les meves prioritats, establir-les i això que hagi de fer en aquell dia ja marcar-m’ho i ja sé que si a lo millor hi ha algun dia que tinc tantes coses que he de fer en aquell dia sí o sí que ja el que faig és marcar-me pot sipar, també, no? un horari, de manera que jo ja sé doncs, mira el que no he pogut fer abans d’acabar la feina jo ja m’he marcat després pot sopar, per tant el temps que jo tinc amb el meu fill el puc dedicar...

**E: Està bloquejat, cent per cent. Molt bé.**

P: ... i ja està, no? I crec que això ha sigut un replantejament arrel del programa que m’he fet i que m’he establert. I també m’ha ajudat a nivell físic, no? Perquè abans el gimnàs era una mica subjecte a l’hora que jo aconseguia sortir de la feina i el fet de poder-m’ho marcar molt més, doncs ara estic anant al gimnàs pràcticament cada dia, no? Per exemple. Eh i clar això al final m’ajuda a nivell físic i també m’ajuda al meu benestar general, no? Per tant, es retroalimenten (AG.4)

1. **E: Molt bé. Me n’alegro molt. Eh... alguna dificultat o algun repte que hagis tingut per completar el programa, si és que els ha hagut?**

P: Sí, sí eh de fet, bueno, el tema de les actes, no? que de fet t’ho vaig comentar, eh ostres no, de fer una acta després de cada meditació (EO.1), no? Per una banda eh em costava ja el fet, Bueno, i a l’inici ja una mica el fet en sí de tenir... o sigui sentir-me entre cometes obligat a fer la meditació no era un sentiment que m’agradés eh però era, com que al final estava incorporat en el meu dia a dia ja estava. Però el fet de... recordo els primers dies no meditar bé i també jo crec que va ser un dels motius pels quals jo vaig deixar de fer l’acta perquè em provocava pensar a vegades què escriuria després.

**E: (so d’assentiment)**

P: I com que això era una pràctica que ja tenia incorporada i el primer dia que vaig anar a escriure... és que dic “hòstia, no sé què escriure i posaré que he meditat deu minuts i que m’he sentit bé”, no? Perquè no sé, és algo bastant natural. Sí que hi ha dies que pots tenir una meditació més diferent. Al matí normalment em sento sempre igual perquè a final m’aixeco en el mateix estat ho faig en el mateix moment del dia, no? I és curta. Sí que algunes vegades quan faig a la nit ja són més llargues i llavors i de vegades és una visualització, de vegades... i llavors aquí sí que hi ha vegades que et podria explicar, no? sensacions més concretes perquè “hòstia perquè aquest cop ha sigut una meditació molt profunda” o no sé, sensacions que hagi pogut tenir a nivell corporal però, no sé, em sentia com obligat a això, a sentir algo diferent o algo característic i no em va... o sigui, això, no... i per això jo crec que també va ser un dels motius de dir, hòstia, deixem-ho. No ho vaig fer conscientment el tema de deixar d’escriure les actes però crec que també, ara pensant-ho així.. post, eh va ser una cosa que no em va acabar d’encaixar dins de la rutina, però bueno segurament doncs pel fet de ser una afegit a algo que ja feia.

1. **E: Vale, eh... en quina mesura ha coincidit aquesta formació amb les teves expectatives inicials, si és que les tenies?**

P: eh, crec que les ha superat (O.1) en el sentit de que les expectatives respecte el contingut que tu has pogut oferir doncs eh, s’han complert. Però sobretot on s’ha superat és sobretot al respecte de les interaccions que han esdevingut, no? amb diferents companys amb més o menys experiència dins del mon del *mindfulness*, eh però crec que s’han generat interaccions molt positives i has aconseguit tu generar uns debats, unes intervencions que a mi personalment, crec que han estat el més positiu del... d’aquest programa (O.2).

1. **E: Super. Bueno llavors això que... la següent pregunta, molt similar, és què destacaries positivament del curs?**

P: Eh, això mateix. El...

**E: Aquestes interaccions.**

P: Exacte. Les interaccions que s’han donat entre els diferents... entre els diferents participants.

1. **E: I, anem acabant, eh. Com valores aquest programa pel teu rol professional?**

P: Crec, no pel meu, crec que per qualsevol rol professional, o no ja professional sinó vital, eh és més enllà d’interessant, jo ara que l’he passat dic “ostres, és necessari”, no? per tothom i tant debò tots, i ja no en un club de futbol o en una empresa, sinó des de petits ens eduquessin amb aquesta visió, no?

1. **E: Sí, sí. Eh i ja l’última. Quines millores implementaries si és que ho faries?**

P: Ostres és, és complicat de dir. Potser sí que una de les... de les millores seria, en el meu cas, no? és buscar una mica eh diferents no estats sinó... no vull dir-li nivells, però... no? per exemple ostres, algú poder dividir entre gent que no ha sentit parlar mai del *mindfulness* eh gent que n’ha sentit a parlar però mai ha aplicat res i gent que una mica ja sap de què va i... jo que sé, doncs té una pràctica de meditació més o menys rutinària, no? Crec que això encara ajudaria sobretot en aquestes interaccions, a poder anar més enllà, a poder ser més específic. Eh i representaria també un repte per tothom. (EO.1) Perquè sí que alguna vegada jo no vaig voler intervenir perquè, ostres, a sigui estaven tractant coses que potser per mi ja eren experiències per les quals havia passat feia bastant de temps i sobre les quals ja havia reflexionat molt i parlat molt i, per tant, no em convidaven a intervenir com segurament a un altre que no havia tingut cap experiència, quan parlava algú que havia tingut una mica més d’experiència tampoc el convidada a intervenir perquè deis, hòstia, jo estic molt lluny, no? d’aquesta persona. Bueno, crec que seria una de les qüestions que crec que podrien ajudar.

1. **E: Perfecte, Quim. I res, si vols afegir algun comentari final.**

P: No, bueno agrair-te a tu i felicitar-te al mateix temps (O.1) per haver, o sigui ja no portar això, però sobretot la valentia, no? de tirar-ho endavant que sé que per tu és normal i hauria de ser-ho per tothom, però moltes vegades, com dèiem abans, són coses que de vegades cal ser una mica valent per poder dur... tirar endavant en determinats contextos. I crec que el Barça és un d’aquests contextos on hi ha determinades coses que, pel fet de ser un club molt gran i tothom doncs veure possibilitats de projecció, ningú es vol mai tallar les ales fent un pas més gran ...

**E: En fals.**

P: Exacte. I llavors és un club on acostuma a ser tothom molt conservador i a no prendre decisions per tal de no cagar-la. I el fet de que tu diguis, no, no, anem a cagar-la, no? Vull dir, no tenir aquesta por (O.2), crec que és molt positiu i et felicito i t’ho agraeixo (O.1).

**E: Moltes gràcies.**

**ENTREVISTA 12. PAU (Entrenador, 31 años) – Código P.12**

**Realizada *online* el día 13 de abril de 2021**

**E: L’entrevista al final el que busca és aconseguir informació, es diu informació qualitativa, però bàsicament és conèixer l’experiència subjectiva. No hi ha respostes correctes o incorrectes, simplement conèixer la teva experiència.**

1. **E: La primera pregunta és: com diries que el programa t’ha impactat, si és que ho ha fet d’alguna manera?**

P: Sí, sí, m’ha impactat (O.1). O sigui, jo al final entrava a la formació des d’un objectiu de curiositat. És a dir, n’havia sentit a parlar, però mai lo suficient o no m’havia tirat prou com per introduir-m’hi de forma autònoma, llavors el curs que proposaves era una opció per algo que em sonava i em podia ser interessant, doncs per vivenciar-ho i viure-ho de primera mà. I la veritat és que la resposta jo la valoro molt positivament (O.1), perquè al final dona l’opció a reconèixer, identificar coses del teu dia a dia que evidentment pots optimitzar o que no li estàs donant la importància que toca (AG.5) i que, evidentment, que a nivell de conscienciació, de fer-te conscient de certes coses, sens dubte (PE.4).

**E: Destacaries algo a nivell personal o professional?**

P: Sí, en general, sobretot aquest punt, que és bastant genèric, però el de l’atenció conscient (AG.1). Si que jo tinc activitats... per exemple, quan estic al camp, per mi, és molt fàcil estar connectat amb l’activitat, és a dir ja m’hi porta *per se*; sempre i quan tingui aquesta participació activa en la sessió. A vegades quan assumeixo un altre rol, el punt aquest d’estar concentrat i sí que m’exigeix un punt més (PE.3). I després al final en el dia a di, aquestes coses que potser li dono menys importància però també hi són presents (PE.2). I ja et dic, gaudeixo molt del disseny, de les tasques, quan estic parant atenció hi gaudeixo molt, però sí que hi ha altres punts del meu dia a dia en que vaig amb pilot automàtic i no sóc conscient de certes coses que a partir del curs... ostres, potser canviar els hàbits és molt gros, però ser conscient d’això ja ho valoro com un punt important. (PE.4)

1. **E: Molt bé. Has incorporat alguna estratègia o algun comportament o algun aprenentatge, tant en la teva vida personal com a nivell professional?**

P: Bueno, la meditació no he estat capaç d’introduir-la dins la meva setmana de forma regular... és a dir, no he estat capaç de trobar els horaris, però sí que trobo els moments de fer una pausa, de potser quan acumulo molta feina seguida o preveig... o em poso fer aquesta feina, de trobar primer un punt abans de fer-ho de parar, de respirar i de entrar com a l’activitat més dura o més plaent amb aquesta meditació prèvia que em permet com buidar una miqueta dels aspectes previs que he viscut (PE.2). Lo del tema dels blocs de treball que comentaves m’ha servit molt... (PE.3)

**E: Vale...**

P: O sigui que... és algo que m’ha ajudat a estructurar potser aquesta activitat que em costa més posar el focus i després sí que en certes activitats de les que deies de S.T.O.P., de parar atenció plena sí que les incorporo molt (PE.2).

1. **E: Eh... què diries que ha sigut lo més beneficiós per tu del curs?**

P: Buf... sí valoro molt positivament (O.1) el fet d’introduir-me a una activitat amb gent que la domina (EO.1) i del final un grup que comparteix les experiències, és a dir que al final estàs vinculat, no és una cosa 1 per 1, sinó que estàs vinculat a una sèrie de gent i és el punt aquest de que et fa estar lligat, et fa estar connectat i et fa estar pendent i actiu i buscar certa continuïtat (RI.3) que potser per... o sigui de forma autònoma no hagués estat capaç de fer-ho.

1. **E: Vale. Fantàstic, eh... algun repte o alguna dificultat per completar el curs? Hi ha hagut algo que se t’hagi fet més difícil? Has comentat el tema de l’àmbit de la meditació...**

P: Sí... valoro molt positivament el conèixer-la i llavors estic en el punt de descobrir dins del meu dia a dia com li dono utilitat o com li dono continuïtat perquè sigui igual algo més prolongat en el temps o més habitual o més constant.

1. **E: Vale. En relació a les expectatives, si les tenies, en quina mesura ha coincidit la formació amb les teves expectatives, si és que en tenies?**

P: Sí, venia amb la ment molt oberta a descobrir una cosa i crec que l’he descobert amb bona manera i molt satisfet de com l’he descobert (O.1). O sigui no tenia massa expectatives prèvies de lo que em podia trobar, sinó que partia amb la ment oberta i de lo que he descobert, de lo que he incorporat molt satisfet (O.1).

1. **E: Fantàstic. Eh... potser es repeteix una mica, però si haguessis de destacar algo positiu del curs, que seria?**

P: Lo que no t’hagi dit abans?

**E: Bueno no, pots repetir, pots... el que tu creguis, pot ser que ho hagis dit abans. Amb què et quedes?**

P: El sentiment de sentir-te acompanyat (RI.3) i de descobrir una cosa amb gent que la domina (EO.1) i aquesta col·lectivitat de que potser experiències que comparteixen els companys o que discuteixes amb companys de forma externa o que et sorgeix via curs, doncs és aquest punt de sumar experiències també dels altres (O.2).

1. **E: Vale... ja acabant, com valores aquest tipus de programa pel teu rol professional?**

P: Doncs la veritat és que molt (O.1). És algo que em permet millorar, que al final venia amb la expectativa de, ostres, pot ser una eina útil per millorar, no només jo, sinó poder millorar... al final treballem en futbol o dediquem gran part del dia amb el futbol que és compartir amb moltes persones i molt diverses (RI.1). I el fet també de conèixer una pràctica que potser jugadors amb els que convius la tenen interioritzada i poder comprendre’ls, sens dubte ajuda. És a dir, ajuda amb la perspectiva pròpia de que ostres, et dona certes eines per ser conscient de coses (PE.4) que no... que pots millorar en el teu dia a dia, que pots optimitzar, que pots millorar aquest hàbit (AG.5)... i després et dona el bagatge aquest d’informació per poder traslladar o si més no poder comprendre en gent involucrada en aquesta pràctica o gent que la veu molt útil i la té molt interioritzada en el seu dia i és aquest punt que a mi em resulta útil.

**E: Super...**

P: Vas posar exemples de diferents futbolistes, i a mi això em va “tirar” (EO). Al final hem d’intentar comprendre una mica el jugador, en context futbol, però hem d’intentar comprendre la gent amb la que convivim (RI) i el punt de tu tenir informació sobre una pràctica que ells la consideren útil ostres, jo ho veig molt rellevant (CT.4).

1. **E: I la última, alguna millora que implementaries en el programa?**

P: Dins de la formació teòrica... no és millorar, al final es com ajustes els temps amb el contingut de la pràctica. Que amb els continguts que vols exposar, és algo que com a professors ens veiem implicats... fins a quin punt profunditzo amb el temps que tinc. Per exemple, el tema de les emocions és algo que em mola molt, i des de la meva visió pròpia m’agradaria molt que es desenvolupés (EO.4), que anés a profunditzar, concretar certes coses... però evidentment hi ha el factor temps, el factor de haver de triar. Suposo que en el teu disseny et succeirà, de fins a quin punt puc profunditzar i tocar tot el que vull tocar.

**E: Clar. Més temps, al final...**

P: Sí.. no, no, i tant. Seleccionar per tu el que és important i allà potser donar-li més... però al final depèn de tu com a professor què valores i depèn de cada alumne o cada participant som un món i, ostres, quines inquietuds realment tenim. Però dins de, ostres, valoro molt com està organitzat i com has estructurat els continguts (EO.1). Crec que hi ha una línia que pots seguir i anar profunditzant, després sí que hi ha el punt de la informació complementaria (EO.3) que evidentment tota no l’he pogut consumir, però ostres, vaig veure la ponència del temps...

**E: Del Sergio...**

P: Sí. I em va tocar, em va tocar perquè hi ha molta informació que ostres...

**E: Bueno...**

P: I també els que tenim adquirits. Doncs això, la possibilitat aquesta de que la gent si li interessa el tema pugui profunditzar que també es una miqueta això de fomentar que els participants tinguem aquest punt d’autonomia.

**E: Correcte. Bueno, en aquest sentit poso una o dues de Sergio, però de Sergio Fernàndez me n’he tragat 10 o 15. Sí que te n’adones que ell va repetint molt el mateix missatge, però et dona moltes claus pràctiques. Hàbitos habitos, hàbitos...i a mi es un tip que m’agrada.**

P: Si, si, si...Clar, es aquest punt de que sempre sapiguem filtrar, de que no ens creguem res. Ostres, que et plantegis certes coses que et facin dubtar i després ja veurem aquest dubtar on et fa tirar... jo sempre ho valor positiu, si et toca és que algo hi ha...

1. **E: Algo hi ha, algo hi ha... Res, si vols afegir algun comentari final, algo que hagi quedat i voldries dir...**

P: Ostres, agrair-te la feina a tu i al grup per la predisposició i tot el contingut (O.1). Entenc que dissenyar tot això porta temps i el dur-ho a terme encara més. O sigui que m’ha agradat molt, m’ha despertat moltes coses que a vegades dins del teu ser vas amagant o coses que vas posant a sobre i altres coses per anar tirant i ostres, que algú et punxi allà et fa remoure coses i et fa hagis de modificar o replantejar-te coses. I aquest punt el valoro molt (O.1).

**E: Molt bé, Pau. Moltes gràcies a tu per la confiança i, si al final a la gent li impacta, aquesta és la idea.**

P: No, és això que al final és algo molt extens, per una banda o per altra algo et toca.

**E: Ja...**

P: O sigui, és tant extens i tants temes que ostres, algun sí tenim...

**E: Mínimament, no?**

P: Sí, que amb tanta gent algun li has tocat per una banda o per una altra i ostres, això ho veig molt interessant. Al final creixem i millorem a partir d’això, de que et toquin certes coses, t’ho remoguin i hagis de fer la construcció de nou.

**E: Correcte. Gràcies Pau, per tot, vale?**

P: D’acord. Molt content de compartir aquesta estona per tu.

**E: Merci guapo. Vagi bé...**

P. Vinga... Ens veiem divendres.

**E: Adéu maco.**

P: Adéu.

E**NTREVISTA 13. MARC (Director Academia Internacional, 34 años) – Código P.13**

**Realizada *online* el día 26 de mayo de 2021**

**E: La idea es donar informació més qualitativa de la teva experiència al programa, amb sinceritat, vull dir que no hi ha respostes correctes ni** **incorrectes, vale?**

P: Vale

**E: Llavors pots començar a presentar-te, nom i el càrrec.**

P: Vale sí, sóc en Marc, director (riu) actualment de la Barça Academy Brasil, però futurament de la Barça Academy Katsushika.

**E: Molt bé.**

P: (Riu)

1. **E: ¿I com diries que el programa t’ha impactat, si és que ho ha fet d'alguna manera?**

P: Pues mira, la realitat és que el rollo de la meditació i de més, *pues* sí que ho coneixia i de més, però no profundament, no m’havia introduït així en els paràmetres de què és la meditació, de què és l’exploració del cos, de què és el moviment conscient, les pràctiques informals no, que sembla mentida, però agafar consciència realment de les coses que fas, com que les sensacions són més... no les fas per fer-les, sinó que collons, rentar-me les dents, o rentar-me les mans o simplement la meditació diària que com et vaig dir l’acostumo a fer a la nit perquè és un dels moments on estic molt més tranquil i tothom està dormint i aprofito, i des de fa molts anys les nits són el meu moment.

**E: Molt bé.**

P: I al haver-me introduït la veritat que collons, *bueno,* vaig dir, ja que ho faig, ho faig bé. Ja has vist el registre, ho vaig fer diàriament.

**E: Brutal.**

P: I anava intercal·lant també exploració del cos, sensacions... que això sempre era molt inconscient no, la sensació que jo tenia al cos, però agafant consciència veus que realment, hòstia, sents tot el que estes sentint en el... en el teu cos, i veus que et va tirant i què està en el seu lloc i què no, saps?

1. **E: i a nivel profesional, destacaries alguna cosa?**

P: *Bueno,* a nivell professional no ho he pogut desenvolupar perquè estic aquí a... a casa, i no tinc grups de treball i de més, però sí que m'he quedat tot el... tot el material que has passat, perquè sí que m’agradaria començar a fer coses una vegada visqui al Japó, quan estigui estabilitzat i vegi com va avançant tot, eh, implantaré aquestes petites coses per... més que res per la part pràctica.

**E: *Bueno*, eh, són la meca, la meditació zen i tot jo crec que et pots impregnar allà de coses xules.**

P: Sí.

1. **E: Vale, has modificat o has incorporat algun comportament o estrategia a la vida personal i vida professional?**

P: *Bueno* la... el tema de fer-ho cada dia jo crec que m’ha ajudat a agafar-me a aquests moments de tranquil·litat i de consciència, i que jo crec que això ja és un canvi perquè molts cops que tu deies del pilot automàtic sempre esta present i hem d’intentar una mica treure aquest pilot automàtic encara que a vegades aparegui, eh, i agafar consciència de les coses. I això m'ajuda també en comportaments, emocions, sentiments... no he tingut cap problemàtica, però jo que sé, en estabilitzar un comportament i no explotar en el moment sinó pensar-hi, saps?

**E: Aha. Respondre no reaccionar, no?**

P: Exactament. Però *bueno*, encara no ho he portat a la pràctica, però sí que és veritat que en el meu cap he agafat consciència d'aquestes coses.

1. **E: Ja, correcte. Vale vale vale... Què ha sigut el més beneficiós per tu?**

P: Jo per mi conèixer què és el *mindfulness* i adaptar-lo una mica a la meva vida, jo crec que és lo més beneficiós.

1. **E: Vale brutal. Algun repte que hagis tingut per completar el programa?**

P: *Bueno*, eh, en els minuts que anava guanyant de meditació, sí que és veritat que sóc una persona molt enèrgica, que em desconcentro moltes vegades, o sigui, les coses com són, a vegades m'estan parlant i tinc el cap a un altre lloc, saps? Li vaig fent Sí sí ja ja ja (riu) i endavant, però sí que és veritat que he anat guanyant temps de concentració, eh, el cap no se m'anava tant els meus pensaments i lo que tenia que fer i demés i la veritat que a lo millor el primer dia sí que feia menys minuts, però acabava, perquè era la meditació de 10 minuts o 15 o 20. Però realment he anat guanyant aquest temps de... com de concentració, no?

**E: ja ja ja**

P: La respiració, en el moviment del cos en la exploració del cos he anat guanyat no m'ha costat, no hi havien interferències.

1. **E: ja ja ja ja ja. Vale, expectatives inicials: en quina mesura ha coincidit** **la formació amb les teves expectatives?**

P: A veure, si et sóc sincer, no tenia expectatives quan vaig veure el programa, però sí que és veritat que a mesura... *bueno*, des del primer dia, em va semblar molt molt interessant i enriquidor (O.1) i això em va fer *pues* que collons m'agafes amb... amb serietat, no? Al fer les activitats diàries, la pràctica diària i sobretot el conèixer una miqueta la història del del *mindfulness* i els comportaments, les emocions que podia donar, que això també et fa una mica, no estar alerta, però saber com reaccionar davant la ràbia, davant de la tristesa, davant de la felicitat... no? Que de vegades no ets conscient de com reacciones.

1. **E: Molt bé. Què destacaries positivament el curs?**

P: *Bueno*, eh, positivament tot el material que ens has entregat i la dedicació que has tingut per.. per fer-lo arribar i de la forma que ens ho has fet arribar, perquè no és lo mateix que tu facin arribar així en un marc teòric pfff... que a mi, si no em fiquen exemples, no em fiquen tal... a vegades és com que desconnecto, però quan m'ho fan així d'una forma que m'enganxa, eh, estic concentrat amb lo que s’està diuen, interessat, saps? O sigui, va anar bé que diguéssim.

1. **E: Ja les últimes dos, com valores aquest programa pel teu rol professional?**

P: Home, jo crec que és algo que no havia pensat, crec que és algo que inconscientment jo li donava molt... li he donat sempre molta importància, o sigui, a com se sent el jugador, a com se sent el meu *staff* tècnic o el club en general, com està aquest ambient, no? com és el context del club i jo crec que això m'ajudarà a prendre consciència i a saber. o sigui, ja ressaltar la importància que té de que tothom estigui en el seu... el seu estat de *flow* tranquil, que pot fallar que no hi han aquestes presses, aquestes bronques de perquè no, o sigui si he fallar lo que tu vulguis però... ajuda, no? O sigui dona la tranquilitat de que podem fallar.

1. **E: Sí, brutal no no, està clar. Vale, eh, implementaries algunes millores?**

P: *Bueno,* jo sobre tot en el temps, que suposo que això sí que, clar, el curs estava com molt reduït en sis setmanes una hora i mitja, eh, si que m'hagués semblat interessant allargar aquest programa, no? I sobretot, *pues*, aquesta hora i mitja pues entre els intercanvis de la gent que parlava, que parlava de les seves experiències i tal, suposo que amb una mica més de temps haguéssim arribat a més profunditat, ja no conèixer-te a tu mateix, sinó també als companys, a la gent que està formant part d’aquest programa.

**E: Ho allargaries, no?**

P : Sí, sí sí.

1. **E: Vale, algun comentari final, algo que no t'hagi preguntat que vulguis dir?**

P: No res, més que res agrair-te també que hagis fet part del teu doctorat i ens ho hagis exterioritzat als que hem volgut, i la veritat que ja et dic, m'ha semblat molt molt interessant, perquè és que no m'havia introduït jo mai al tema *mindfulness* ni això ni les meditacions o a moure conscientment el cos, eh, ja sigui per començar o acabar el dia, que això és molt interessant, són pràctiques quasi inconscients encara que siguin conscients el teu dia a dia a la teva setmana.

**E: Perfecte, vale. Moltes gràcies.**

**ENTREVISTA 14. GUIEM (Entrenador, 27 años) – Código P.14**

**Realizada *online* el día 26 de mayo de 2021**

**E: Vale, Guiem, tu ets entrenador del futbol formatiu del Barça i has participat en el programa de *Mindfulness* per Entrenadors. I, *bueno*, la idea és complementar el tema dels qüestionaris, que és informació quantitativa, amb entrevistes individuals que es consideren qualitatives i on bàsicament pugueu manifestar la vostra opinió sobre el programa.**

P: eh...

**E: Sí, digues.**

P: No, dels questionaris jo volia dir una cosa, que clar jo el vaig fer com, eh, entenc que l’1 o el 2 o el 3 o el 4 o 5 era com com un valor, però clar ara canvia la meva percepció i llavors igual en un inclús he ficat més però perquè soc conscient de que observo més coses d'aquestes, saps que vull dir?

**E: ja**

P: I observo més que abans, jo sé que sóc conscient de lo que faig a lo que podria fer, el meu 5 abans igual era pues jo que tenia ni idea i ara el meu cinc està *pues* més enllà, doncs el meu 2 com diguesim està mes endavant de lo que estava abans el 3.

**E: Aquesta és una reflexió que hauré de fer quan em vegi el qüestionari i per això és molt important la l'entrevista ara, no? Que t'ho puguis dir, hòstia, *pues* sembla que potser en els qüestionaris dona que no em sé regular millor emocionalment, però realment sóc molt més conscient, per tant, ho considero com un progrés important, no?**

P: Sí, clar.

1. **E: *Bueno,* anem pregunta per pregunta. Com diries que el programa t'ha impactat, si és que ho ha fet d'alguna manera?**

P: Eh, m'ha ajudat a ser més conscient de coses que abans, *pues,* era més inconscient, no? Feia o posava el piloto automàtic. I també m'ha ajudat més a disfrutar de o a viure el present, no? Jo què sé... amb la conducció, per exemple, conduir el cotxe ho noto un *montón*, perquè abans i noto que ara em pasa a vegades, però abans era com era com casi perdre el temps agafar el cotxe i tal, saps? I ara ho disfruto, saps, que hi ha moments que dic, hostia va em ve de gust anar-me un rato amb el cotxe em foto la música i vaig mirant, vaig fent, i abans era com... jo que sé, feia mil coses a la vegada mentres conduïa.

**E: Ja, *multitasking,* no?**

P: Sí.

**E: Ja...**

P: També al gestionar com al dia a dia (AG.4), perquè abans era com... notava que estava molt més pensant en lo que vindrà que en lo que estem fent ara, i era com moltes més preocupacions per allò que vindrà, i de dir, doncs mira, doncs ja veurem com acaba lo que vingui, però anem a pensar en l’ara, no?

**E: I a nivel professional, destacaries algo?**

P: Això que et deia de centrar-me en l'ara. *Pues* que lo que vingui ja vindrà i a centrar-me en això, sobre tot aplicat també al món professional.

1. **E: Llavors, has modificat o has incorporat algún comportament, alguna estrategia tant a la teva vida privada com professional?**

P: Sí, meditació (PE.1) intento fer cada dia una, cap al mitg dia és quan, quan més m'agrada, ara estic... Estic fent de 24 minuts cap amunt.

**E: Molt bé.**

P: Algunes setmanes si que és veritat que em costa una mica més perquè ara com estic tinc el *rato* tranquil a casa i em fico i estic tranquil a casa; els caps de setmana entre partits, no sé que, arribo, quan arriba la nòvia o els amics volen aprofitar per fer coses és com que no tinc aquest moment i em costa més, però entre setmana sí que faig una cada dia.

**E: Molt bé.**

P: I també, *bueno*, abans dels partits pues també intento tenir un rato així de de respirar i de relaxarme, també, durant els partits estic bueno m'ho noto que estic com mes centrat en lo que pasa i no tant pensant i anticipant coses que després no tenen perquè pasar (PE.3).

1. **E: Ja... Molt bé. Eh, vale, què diries que ha sigut lo** **més beneficiós del curs per tu?**

P: Eh, no sé, com la la manera d'afrontar-ho tot i de veure-ho, o sigui, que aquest canvi com si diguéssim de perspectiva (CT.2) que ja et condiciona tot el que fas.

**E: Com explicaries aquest canvi de perspectiva?**

P: Eh, *bueno*, també en el reconeixement de mi mateix, d’autoconsciència (PE.4) de moltes coses, aixo és veritat que fins ara lo que deia abans que com que anava fent i ni et paraves a pensar, i ara sí que em reconec molt més o veig, *pues,* jo què sè, ara estàs més nerviós, ara menys, i també amb la autoregulació (AG.2), és una cosa que he notat i si, en això, eh, en no estar tan preocupat per voler controlar i com deixar-me més i anar fent.

1. **E: Molt bé. Eh, algún repte o dificultat per completat el programa, si és que les has tingut?**

P: *Bueno*, lo que ja he dit de fer les meditacions el cap de setmana m'ha costat una mica, les exploracions del cos també em costaven més que la meditació normal, i el tema del material complementari que anaves enviant se m'ha se m'ha acumulat o sigui no he pogut veure tot, ho tinc alla en pestanyes obertes sobre tot els *links* els tinc allà que quan tinc un *rato* m'ho vaig ficant, però sí que és veritat que en el dia a dia no m'ha donat temps de veure-ho tot.

**E: Pensa que és molt eh, o sigui, una cosa és el material complementari que posava als *mails*, però l'altra és que al manual i ha molta més cosa, la idea que tinguéssiu un suplement.**

P: No, jo ho tinc allà i quan vagi tenint mes *ratos* ho seguiré anant fent per anar... Per anar tenint més informació, però si que és veritat que no m'ha donat temps de mirar-lo tot.

1. **E: Molt bé. Eh, en quina mesura el programa ha coincidit amb les teves expectatives, si és que les tenies?**

P: Eh, bastant, ho sigui, no tenia moltíssimes expectatives, però ha estat més del que esperava, ho sigui, crec que m'ha aportat molta més informació, que jo, *bueno*, com que era bastant desconeixedor (CT.1), *pues* no ho sabia que hi havia tanta informació.

**E: Aha...**

P: Sí, molt millor del que esperava (O.1).

1. **E: Molt bé. Eh, vinga, ja anem acabant. Què destacaries positivament del curs?**

P: Eh, no sé, és que tot en general, ha estat, ha estat molt bé, no? El fer grup ha estat bé (RI.3), de veure, *pues,* que s'estava a gust a les reunions i les eines per la meditació (PE.1) i pel moment present també m'han anat molt bé perquè a vegades és com que et ficaves allà i dius i ara que faig, saps?, i era com, *bueno,* m'han anat be els ancoratges (PE.5), el com portar les meditacions sí que m'ha ajudat molt.

1. **E: Molt bé. Eh, vale, ja la penúltima. Com valores aquest tipo de programa pel teu rol professional?**

P: Bé, molt bé, molt satisfet i molt necessari crec (O.1).

1. **E: Vale, i la última ja. Implementaries alguna millora al programa?**

P: Eh, no, *bueno* no no conec tampoc més que tu, o sigui, que si tu vas decidir fer-ho així també està molt bé, a mi, si s’hagués pogut fer coses presencials *pues* segurament hagués sigut mes ric (EO.4) o, *bueno*, hagués sigut també més fluit el, jo què sé, poder-nos trobar en lloc de fer-ho per video trucada *pues* ajuntarnos i fer-ho, *pues*, segurament hagués sigut mes... Hagués fet la experiència encara més rica, però vaja, que és l’únic que se m’acudeix que podria fer diferent, i més temps (EO.4), a mi m'hagués agradat de seguir.

**E: Ràpid, no? Curtet...**

P: Sí, com que encara falta molt per anar profundint i anar coneixent.

1. **E: Vale, Guiem, vols afegir algún comentari final, alguna cosa?**

P: Eh, no, crec que no, m’hagués agradat també saber com ho aplicaveu també al juvenil B i tal que ho vas con anar comentant. Jo ho he parlat alguna vegada amb el tutor de l’equip, sobre tot de quan feiem dinàmiques a casa, sí que feiem alguna meditació, però res, 3 minuts, 4 minuts al final de la sessió. Clar ara amb els entrenos que a més no tenim vestuaris, *pues*, era com més difícil, si haguéssim de tornar i haguéssim aprofitat també perquè estàs com, jo què sé, abans d'algún partit així mig important o a l’hotel que tenim *ratos* morts *pues bueno*, tècniques d'aquestes, pero al aplicar-lo amb els jugadors jo a vegades que lis he dit quan estem alla acabant el escalfament que si estan nerviosos que respirin que tal i si que veus que quan un jugador comença a a fer (soroll de inspiració) saps també que esta nerviós per relaxarse i tal i li fem prefuntes i estrategies que fan servir pero si que m'agradaria saber també com tenir mes eines per aplicar-lo també als jugadors (PE.3).

**ENTREVISTA 15. ENRIC (Entrenador, 29 años) – Código P.15**

**Realizada *online* el día 26 de mayo de 2021**

**E: Moltes gràcies per participar en aquesta entrevista. L'objectiu és una avaluació qualitativa del programa, saber la teva opinió; no hi ha respostes correctes ni incorrectes, vale? Per confirmar-ho: Enric, ets entrenador de la Barça Escola de Barcelona.**

1. **Com diries que el programa t’ha impactat, si és que ho ha fet alguna manera?**

P: Hòstia, doncs ja t'ho vaig comentar ahir amb el *feedback* i la veritat és que a mi m'ha servit de moltíssim (O.1), li hem posat nom o consciència a potser allò del que jo no era conscient, en anys anteriors o com jugador, i al final això m'ha servit de moltíssim i de ser ara conscient de... dels nostres pensaments o de lo que pensava o dels estats que tenia quan jugava a futbol. Hem parlat l'estat del *flow* que jo sabia que em sentia molt bé en alguns partits i en alguns altres no tant i... això, o sigui, m'ha servit de moltíssim, sobre tot per posar-li nom a allò que era inconscient.

**E: Allò que ja havies experimentat alguna vegada, no?**

P: exacte exacte havia experimentat però no sabia ni el perquè, ni ni el com ni el perquè es donava aquella situació i perquè a vegades es dona i a vegades no, com podia o sigui em preguntava perquè no estic sempre així, o sigui perquè no puc em sentir sempre d'aquesta manera i clar això m'ha fet jo no era coneixedor evidentment i això m'ha fet coneixer realment ho que sentia i el perquè de moltes coses

**E: I a nivel professional ¿com diries que t'ha impactat?**

P: Doncs bé, o sigui, perquè al final sí que és veritat que hi han molts moments de l'entrenament què desconectes una mica i... també no saps el perquè, però ara doncs quan desconnectes ets conscient de que estàs desconnectant (PE.4). I que has de tornar, o sigui, has de tornar al moment present que és el moment del l'entrenament, o sigui, al final doncs te n'adones d’aquestes distraccions a nivell professional i també a nivell de vida quotidiana quan estic fent un treball, doncs intento deixar el mòbil apartat, perquè sé que és una distracció; potser abans l'agafava, ara intento no agafar-lo, quan estic divagant perquè moltes vegades te'n vas amb pensaments, doncs, hòstia, ara he marxat he de tornar i me n’adono d’aquestes distraccions (PE.4) que abans, segurament, podia estar més *rato* distret. O sigui, em distrec igual, però puc saber que m'he distret... i tornar rapid en el moment present. O sigui, m'ha servit molt per això. També a nivell de vida quotidiana ja t'ho vaig explicar que bueno vaig amb bici de carretera i això i era moments que clar, estàs tres hores en bici que et menges molt el tarro, igual que quan vas a córrer, a mi no m'agradava anar a córrer perquè et menjaves l'olla i no m'agradava. Al final en bici em passava més o menys lo mateix, hi havia dies que fins i tot acabava amb mal de cap i no sabia si era pel tarro que m'havia menjat o...

**E: (riu)**

P: (riu)... o algo. I vaig pensar: mira aniràs en bici i seràs conscient o estaràs pendent de la natura, dels ocells com canten, del sol que et pica a la cara, o ser conscient en el moment aquell que estàs gaudint de l’experiència final, i ho vaig fer i realment em va servir o sigui vaig arribar i dic: hòstia, doncs avui em sento millor que altres vegades que he anat en bicicleta.

**E: Ja...**

P: O sigui, al final, vaig acabar sense mal de cap i dic: hòstia, i això perquè al final m'ha servit, o sigui, he gaudit de l’experiència d'anar en bici, jo quan anava en bici estava molt pendent també de... Sóc competitiu i tinc l’Strava, i vull anar a fer temps a vegades i em menjo el tarro anant a fer temps i dic hòstia puta tampoc pot ser sempre així, deixa’m disfrutar, gaudir del moment, pensar,... Pensar en l’ara, estic escoltant els ocells, la natura, els olors i tio de veritat que em va anar bé, em sorprendre i tot i dic ostia saps és una alegria de veritat i em va perfecte, em va anar perfecte (PE.2).

1. **E: Molt bé. Què t'anava a dir, molt semblant vale, he apuntat coses que has anat comentant, vale? Si has incorporat o modificat algú comportament o alguna estrategia a nivell personal i professional, m'he apuntat per exemple l’ús més conscient del mòbil, evitar les distraccions, alguna cosa més?**

P: *Bueno*, sobretot això, lo que m'ha impactat més es això el moment que... que et distreus doncs ser conscient que t'estas distraient (PE.4); però amb tot, o sigui, estàs entrenant, des de que estàs fent un treball, des de que estàs anant amb bicicleta, doncs al final lo que m'ha impactat més és la consciència, la consciència de lo que està passant en aquell moment.

1. **E: Molt bé. Eh, què ha sigut lo més beneficiós del curs, per tu?**

P: Lo mes beneficiós, en general, per a mi és que es tot: m'agradaven molt les classes presencials, els articles, mirava la gran majoria de vídeos que passaves perquè també m'agrada Víctor Kuppers, ja el coneixia i m'agraden totes aquestes fonts d’informació que que ens has anat passant i també m'han ajudat, o sigui, a conèixer més aquest tema del *mindfulness.*

1. **E: Molt bé. Algun repte o dificultat per completar el programa?**

P: Sí, dificultat el tema meditacions no he pogut ser del tot... com ho diria ser del tot...

**E: Regular?**

P: Regular, exacte, del tot regular, perquè, *bueno*, he anat seguint, però hi havia dies que no feia perquè... el temps no és excusa, ja ho vas dir, perquè podem, però no vaig trobar els moments per fer-ho.

1. **E: Molt bé. Eh... En quina mesura el programa ha coincidit amb les teves expectatives, si és que les tenies?**

P: Sí si... per mi les ha superat (O.1), o sigui... al final jo no coneixia gaire sobre el tema, sí que m'he llegit llibres de... que anaven relacionats amb psicologia positiva, m'he llegit Víctor Kuppers, m'he llegit el llibre de Xesco fa molts anys, el de el de Phill Jackson de once anillos...

**E: Molt bé.**

P: També tinc un altre llibre de Jorge Luengo de *trucos mentales* i així, m'he anat llegint coses d'aquestes i, de veritat, perquè m'agraden, m'agrada saber sobre psicologies positives, saber treure el màxim rendiment de tu mateix a nivell de... a nivell psicològic.

1. **E: Molt bé. Eh, què destacaries positivament del curs?**

P: Què destacaria positivament? Doncs, pfff... és que l'experiència que hem pogut tenir tots, o sigui al final... tu el que ens has compartit i lo que hem aprés també d’altres companys, o sigui, perquè al final ets que poder... Poder... Que no estas tu sol en aquest programa, saps que no tens tu sol aquestes inquietuds, perquè sembla que, a vegades, no sé, sembla un tabu pensar aquestes coses (O.2)

1. **E: Aha... sí sí, brutal. Eh... vale les dos últimes: com valores aquest tipo de programa pel teu rol professional?**

P: Doncs ja t'ho he dit, per mi és genial (O.1), o sigui, és genial pel rol professional perquè al final t'ajuda, o sigui, al final el teu cap és el que funciona o no et farà funcionar i al final aquest programa t'ajuda... a treure lo millor de tu o intentar treure lo millor de tu.

1. **E: Correcte, aquesta és la idea. *Bueno*, ja la última, alguna millora, si és que implementaries algo?**

P: Alguna millora... pfff, hòstia, ja et dic m'ha agradat moltissim, alguna millora no... És que no sé, no la trovaria... Per mi, a nivell personal, si que ser més regular en tot el tema de meditacions, però a nivell de programa, per mi ha set un gran aprenentatge. Molt agraït.

**E: Perfecte, Enric.**

P: Complicat superar-lo (riuen els 2)

**E: *Bueno*, ho intentarem eh, jo proposaré una segona edició, proposaré que** **fessim això més regular i anirà cap amunt perque al final he volgut compartir moltes** **coses en poc temps i llavors ara falta anar de poc a poc, no? Treballant aquestes coses; per tant, ho intentarem superar.**

P: Si si, perquè aquests temes són.. són molt interessants. Perquè al final el cap, tio, a vegades et fa fer coses, et fa pensar coses que dius hòstia no, que costen de controlar i això hem de ser conscients, tio, de dir: hòstia estàs pensant amb això, val? No divagar tant .

1. **Vale, Enric, doncs res, si vols afegir algún comentari final?**

P: No no, res, doncs, que merci pel programa que m'ha servit de moltíssim i, això, aviam si es pot donar continuitat a al *mindfulness* perquè, per mi, he trobat que és una eina clau per trobar el teu maxim rendiment a nivell personal i, això, si afegeixes que ets un entrenador d'un equip, doncs a treure el maxim rendiment teu i del equip (O.1).

**E: Brutal, Enric, doncs moltes gràcies, un plaer.**

P: A tu, Aleix

**ENTREVISTA 16. BERNAT (Director Academia Interacional, 31 años) – Código P.16**

**Realizada *online* el día 26 de mayo de 2021**

**E: Bàsicament, aquesta és una entrevista per analitzar de forma qualitativa el programa que has assistit. Llavors, em recordes el teu nom i la posició?**

P: Bernat, DPL del Barça.

1. **E: Perfecte, eh, Bernat, com diries que el programa ha impactat, si és que ho ha fet d'alguna manera?**

P: Jo diria que sí, que ha tingut un impacte. El que sí que he trobat és més calma, trobar aquest moment del dia en el qual... *bueno, pues* intentava no pensar en res més enllà de de... d’aquell moment i de les coses que, *bueno*, intentava concentrar-me en la respiració. Intentava concentrar-me en diferents parts del cos, intentar concentrar-me en allò per no pensar en la resta. M'ha ajudat en el fet de, bueno, de prendre el dia una mica més amb actitud contemplativa i una mica més tranquil (AG.4).

**E: Perfecte. A nivell professional o personal destacaries alguna cosa?**

P: Bueno, a nivell personal, ho hauràs vist a la fulla de registre, jo ho acostumava a fer al sortir de la feina i nosaltres estem a molta distància de la ciutat. Gairebé si vull anar fins al nord de la ciutat, pot ser puc estar una hora amb cotxe i, llavors, jo el que feia era posar-me un vídeo de de meditació o de relaxació, inclús al cotxe mentre baixava. I molts dies em quedava adormit. Però els dies que no em quedava dormit, arribàva molt més tranquil. Arribava sense l'angoixa de l'alt dia de feina. *Bueno*, a nivell personal, a nivell de feina, però sí que hi ha dies que me'n vaig un moment a fora i faig 10 minuts de respiració tranquil i també ho he notat, eh? Tot i que hi ha dies que no he pogut trobar aquest aquest moment.

1. **E: Vale perfecte. Has incorporat algun comportament, alguna estratègia a la teva vida personal i professional?**

P: El que sí que he incorporat és fer aquest procés 10 respiracions (PE.2), estigues tranquil i bueno, intentem no pensar en els diversos agents que durant el dia, *bueno*, et porten a pertorbacions i sí que ho he notat molt a l'hora d'anar a dormir. Molts cops ho he fet abans d'anar a dormir. Que no ha fet no sé fins a quin punt està mal fet, perquè al final em relaxava i em dormia. Però m'ajudava a adormir-me que hi ha dies que et poses al llit i el cap va donant voltes i no saps quan pararà.

1. **Mmm venga, què diries que ha sigut el més beneficiós per tu?**

P: No està en la conseqüència, sinó també en part de la causa de poder trobar aquests moments el dia el dia. Òstres, *bueno*, ara... ara m'aniria bé això. I vaig dir, ho faig o surto de la feina i dic, òstres, ara he de fer aquest clic, he de desconnectar del moment de tensió, d'estar moltes hores a arribar a un altre lloc on la feina no està involucrada i que no t'afecti.

1. **E: Perfecte, eh, alguns reptes o dificultats que t'hagin sorgit per completar el programa, si els que els ha hagut?**

P: No, m'ha suposat el repte de poder-ho seguir. Si el fet és que nosaltres fèiem la classe el dilluns, dia de festa i per exemple, les dos classes que no vaig poder assistir era perquè *bueno*, *pues* perquè dia de festa te'n vas a la ciutat. *Pues* és poder l'únic, l'únic problema que m'ha sorgit.

1. **E: Molt bé. En quina mesura la formació ha coincidit amb les teves expectatives inicials?**

P: No coneixia gaire el terme de *mindfulness* ni cap a on podia anar. Sí que esperava trobar moments de calma. I els he trobat. I en el moment que va començar el programa em va anar com anell al dit. De vegades, *pues* no tens temps de fer res del dia. A dintre la bombolla de la feina i a trobar aquests moments de sortir. Ara em separo, estic tranquil i això m'ha anat molt bé. Eren les expectatives que tenia en el programa.

**E: Molt bé. S'han complert, diríem?**

P: Si si si. Ara per part meva, crec que lo que em toca és *pues* endinsar-me una mica més en aquest món, perquè si m'ha anat bé, jo crec que, *bueno*, que només hem tocat la punta de l'iceberg. Jo crec.

1. **E: Sí, totalment d'acord. Què destacaries positivament del curs?**

P: Poder-les les eines (PE.5) per poder fer-ho, perquè a vegades fas moltes informacions i no et donen gairebé cap cop cap cap eina, és a dir, òstres, això ho puc aplicar jo puc aplicar gairebé, ja trobar aquests anclatges. I poder anar evolucionant, jo crec que és el punt fort de poder, *bueno,* de poder trobar veure uns resultats visibles des d'un bon inici.

1. **E: Ja les dos últimes, eh? Com valores aquest tipus de programa pel teu rol professional?**

P: Molt necessari (O.1). Jo crec que bueno, que t'ajuda el dia a dia (AG.4).

1. **E: Quines millores implementaries en el programa, si és que ho faries?**

P: Clar és que no, no sé si sóc molt...

**E: Només la teva opinió. *Bueno...* per la teva experiència, tal com has viscut el programa. Vull dir, poden ser millores en quant a format, millores en quant a temps, millores en quant a continguts, etc.**

P: *Bueno,* jo en els que ens vas enviar, sí que vaig posar una cosa que, *bueno*, que a mi m'interessaria, és tenir més material d'aquest complementari. Perquè *bueno, pues* que fa a la pràctica o baixes molt amb el cotxe i dius ostres em posaré un vídeo a veure què m'explica i després poder puc fer 10 minuts de pràctica poder complementar la pràctica. Com amb diferents vídeos de persones que o que ho hagin viscut o que hagin trobat un moment de canvi a les seves vides o persones enteses en la matèria que ja l'has enviat..

1. **Vale Sergi si vols algun comentari final algú que m'agradaria dir però no.**

P: No, no res, merci. Crec que són iniciatives i també ho sé, que és molt de temps el que el que has dedicat a això i, *bueno, pues* s'agraeix. I si de part meva, *pues,* en qualsevol moment puc ajudar en qualsevol cosa com ara poder-te donar una un *feedback* qualitatiu. Més encara.

**E: Vale Sergi, *pues*, moltes gràcies. Qualsevol cosa que necessitis ja saps.**

P: De res.

**ENTREVISTA 17. PERE (Departamento de Metodología, 30 años) – Código P.17**

**Realizada *online* el día 1 de junio de 2021**

**E: Pere, moltes gràcies per participar en aquesta entrevista. L'objectiu és una avaluació qualitativa del programa, saber la teva opinió; no hi ha respostes correctes ni incorrectes, vale? Per confirmar-ho: Pere, ets integrant del Departament de Metodologia.**

1. **Com diries que el programa t’ha impactat, si és que ho ha fet alguna manera?**

P: Hòstia tio, adonar-me que estic en pilot automàtic, saps? És a dir, no estic sent conscient del estic fent. Per exemple, la conducció em passa molt sovint. Ara, des de que vas dir lo de la conducció conscient, l'altre dia que passo sempre pel mateix lloc i tio, no sé si a tu et passa, crec que passo pel radar sense adonar-me de que hi ha el radar, però que automàticament he passat a 80.

**E: A 80, no?**

P: Si vas a 100 i t'has posat a 80 sense ni adonar-te. Llavors penso. Això és una puta bogeria, no? Com que hi ha uns estímuls a la carretera que ja fa que identifiquis on estàs que aquí t'has de frenar, i a vegades segurament ho facis conscientment en un moment donat, però que els 10 segons després dius tu, però m'he parat o no m'he parat, saps clar, no m'ha arribat cap multa, així que. Suposo que sempre em paro, no? Però tinc aquesta sensació, no de de que m'ajuden, sobretot a identificar moments en què no estic parant. No estic fent aquest S.T.O.P. No estic ordenant, ordenant no és la paraula, sinó dient: escolta que estàs aquí atabalat i, *bueno*, de donar una mica de... de *bueno,* de lo que et deia d’aquest S.T.O.P. del dia a dia. Jo crec que prendre consciència d'això (PE.4), sobretot.

**E: I a nivell professional, destacaries algo?**

P: Jo, a nivell professional que també el... com es diu això? El prendre consciència dels hàbits de treball, no? Amb això dels blocs, perquè justament tu em vas presentar-lo als blocs, però jo vaig parlar amb el amb el Jordi A. en el seu dia, no? I justament li vaig fer la mateixa pregunta que et vaig fer. Que jo per la feina que tinc, per les hores amb l'ordinador i per no estar en el camp jo, per exemple, quan era entrenador, em notava que era una activitat que teníem *flow*, és a dir, que era una cosa on tenia clara la intenció, on estava observant els jugadors, on aquestes interaccions eren molt naturals, no? I que la majoria del temps estava present en el moment, i que amb això de la feina amb més d'oficina, a més de preparar i tal és una cosa que em costa molt desconnectar, no? I lo que em fa connectar-me és el *multitasking*, vale? I no em refereixo *multitasking* de contestar un *WhatsApp* i ara un correu i tal, que a vegades també, no? Però *multitasking* de sentir que vaig tancant coses no i això i a mi el Jordi em va dir no és, però això pot ser que estiguis tancat moltes coses, però no estàs sent productiu o no estàs sent eficient, és a dir, estàs com enganyant-te. Has enganyat a la ment de que has tancat això, però que realment si mires el tot, doncs no estàs sent eficient o no estàs sent efectiu i, *bueno*, lo que em dóna és això, és a dir, el pensar o el ser conscient de que m'estic centrant molt en lo dels blocs (PE.3). Ho intento posar en pràctica. Dic: hòstia, posar el mòbil en silenci, i dic si aquests 40 minuts jo estic fent aquestes dues o 3 tasques a l'ordinador, pues no miro el mòbil a veure què passa, i això sí que sí que ho intento aplicar.

**E: Et sents eficient, clar clar.**

P: Exacte i em fa sentir eficient, però a vegades em pregunto si és real, no? si és productiu real o és simplement el que tu dius és simple dopamina.

**E: Endavant no, no jo crec que és real perquè és el teu context. Ara mateix vull dir, no, no ha d'estar bé ni malament. Eh, vull dir si aquesta és la teva dinàmica actual i tu et sents i, jo crec, que tu ets eficient. En això no hi ha cap problema i no hauries de modificar res. Saps què et vull dir? El problema és quan deixem passar per davant certes coses que potser no serien prioritàries. És a dir, quan tu poses a dissenyar el projecte de l'any que ve, la proposta competencial o fas un projecte així més creatiu sí que és moment de tancar tot això, potser, saps? però això t'ho has d'identificar si funciona o no funciona quan et funciona i quan no et funciona. Però no té perquè estar malament lo que tu fas, eh? Tot lo contrari si tu estàs còmode com estàs jo no canviaria.**

P: Quan jo, sobretot, això sí, algo del programa és el prendre consciència de tu mateix (PE.4), és a dir, el tenir aquest aquesta consciència de tu mateix del que estàs fent, de l'activitat que estàs fent, lo eficient que estàs dient. I crec que aquestes reflexions t'ajuda a, *bueno*, lo que tu dius, no? Ni estava bé ni està malament, però t'ajuda a dir: hòstia perquè estic fent això? O com podria optimitzar això, no? I prendre consciència és important.

1. **E: I vale llavors la segona. La segona pregunta era, has modificat o incorporat algun comportament, alguna estratègia aquest moment?**

P: Sí, sí, sí, jo en la vida personal, el que m'ha quedat més pendent és tenir una rutina de meditació, perquè és una cosa que quan la faig m'agrada i em senta bé. Sempre penso: *joder* tio, és que ho hauria de fer cada dia, però no, no sé per què no tinc el compromís. No sé si és perquè visc amb el marc i fem horaris semblants i està despert i és com que dius: hòstia, que està aquí a ara no ho faré o perquè així un dia em diuen de jugar el pàdel a les 8h... no? És a dir, *pues* ja trenca l'horari. Com que estàs com adaptant el teu horari a les activitats a la feina i és bastant flexible. No sé si és això el que m'està fent, que tinc un compromís. Va haver-hi una setmana que vaig fer a la nit, no? Quan tu vas a comentar això, *pues* que hi ha gent que ho fa la nit i ho vaig fer a la nit. Ho vaig fer 3 o quatre dies seguits i vaig dir: hòstia, aquest horari també em van vindre bé, no vinc de treballar i tal em tanco a l'habitació i tal, però és el mateix, és a dir, que està la Berta a casa o està el Marc, em diuen tu, però què fas a l'habitació? Llavors, m'ha faltat compromís per dir: ei, aquest espai és sagrat o aquest aquests dies són sagrats per fer això, no? Perquè una de les coses que jo em proposo a mig terme és dedicar-li temps, perquè et dic, cada vegada que ho faig és com que em senta bé. És una cosa que em relaxa molt (AG.4), que m'ajuda molt i quan acabo a sentir-me com fresc.

**E: *Bueno*, ja veuràs que trobar el moment és important i és difícil a vegades, saps què et vull dir, si tens gent a casa, si hi ha convidats, no? per això d’alguna manera jo em desperto abans que tothom i ho faig, si puc fer-ho, ho tanco.**

1. **Què t'anava a dir què creus que ha sigut lo més beneficiós de tot el curs?**

P: *Bueno*, per mi tu (EO.1), no? i ho dic en serio, que per mi ha sigut d'alta qualitat (O.1). És a dir, jo de fet, t'anava a dir cobra per fer això, saps? Perquè, si si em diguessis hòstia, no és que agafa aquest *Power Point* de l'escola... no ha fet recerca i el fa servir a tot arreu, però no és això. És a dir, se't nota que coneixes vídeos, coneixes autors, coneixes l'essència de diferents filosofies, és a dir, jo, per exemple, estava molt molt atent i m'agrada molt el contingut (O.1). A mi em sabia greu que no es pogués acabar, que no es pogués anar aprofundir. Jo moltes vegades no he participat més perquè he pensat, jo sóc el pesat de *torno*, saps? És a dir, perquè hi ha moltes coses que m'agradaria compartir en petit grup o amb tu i dius, a vegades, hòstia, hi ha un contingut perquè això m'ha passat a mi fent el nivell 3, devegades deia, escolta que jo vull escoltar el profe, vull escoltar el contingut que té i no interrompre per parlar de tu, parlar de coses teves. *Pues* a vegades he intentat mesurar no, però ja et dic que a mi l'entorn em convidava molt (O.2). A parlar del tema a obrir-te a que tu, a més, es notava que tenies un coneixement profund de diferents tendències, a més que no eres ni taxatiu, ni concloent, ni res d'això, sinó que queda tot bastant obert. Era tot bastant reflexiu que és com és la manera que m'agrada que em parlin o que em transmeten una informació.

**E: Aha...**

P: Però *bueno*, ja et dic, jo el curs el veig top (O.1) i el veig top des del moment en què es fa la meditació grupal, ho veig tot des del moment que comparteixes material, no m'he llegit cap cosa, però vídeos els he vist gairebé tots, que fas un seguiment setmanal. Una cosa que et diria de fer, però això sempre i quan cobris el curs, és establir meditacions grupals.

E: És a dir.

P: Jo que sé, convidar pues si hi ha 3 grups o 4 grups que hi ha, 1 vegada al dia o 3 vegades a la setmana, on tu et connectaràs per fer la meditació, saps? i fas una *online* i ho pensava perquè dic hòstia, jo segurament m'hagués trobat aquell espai com un compromís, saps? si hagués dit, van l'Aleix, van els companys del curs... no sé què... jo em connecto, em busco temps per connectar.

**E: Ja... Correcte, 10 minutets clar.**

P: Però clar, això és putejar-te tu perquè has d'estar compromès per connectar-te. Per això, una de les coses que vaig pensar vaig dir hòstia, si fessis una periodització de que la primera setmana ens trobem quatre vegades 10 minuts després, quatre vegades, una altra després 3, després dos saps, és a dir, fas un seguiment.

**E: Eh, *bueno*, aquesta és la idea, és veritat, però també s'ha de veure si el club li interessa o no li interessa la proposta, eh? Ja ho veurem primer si hi ha interès i després ja avaluarem. Però sí que havia pensat fer un dia de classe igual, més o menys el mateix, el mateix model i un segon dia mínim de meditació. Una meditació que es connecti qui vulgui i ja està.**

P: Sí, sí.

1. **E: Vinga va, següent *bueno*, aquí era algun dels reptes o dificultats que t’han sorgit per completar el programa?**

P: Lo que més m'ha costat, és la meditació.

1. **E: Vale la següent pregunta és, en quina mesura ha coincidit aquesta formació amb les expectatives inicials, si és que les tenies?**

P: Bueno més del que m'esperava (O.1). És així, i ja et dic, el contingut em va semblar ric en profunditat. Em va semblar bona el fet de meditar junts (EO.1) em va semblar un valor afegit que *ojalá* hi hagués més. És més del que m'esperava la veritat. I va faltar temps a les teòriques, segurament.

1. **E: Sí, sí, a veure... què destacaries positivament el curs, lo millor, si haguessis de destacar algo, què diries?**

P: La meditació grupal i compartir sobre ella i que va faltar temps per perquè la gent comentés més.

**E: *Bueno,* i nosaltres quedem un grupet de 10 que és l'ideal. El grupet ideal per mi són 10, 12 perquè l'altre eren 25. I clar, hi ha molta gent que no parla. Sí vale dos més Jordi ja estem vale.**

P: Eh, tranquil.

1. **E: Com valores aquest programa pel teu rol professional?**

P: Bueno pel meu i el de tots. Crec que prendre consciència d'un mateix i del que estàs fent és és una cosa que ens han d'educar. I sí, sí, sí, jo incorporaria a qualsevol. Si a qualsevol programa qualsevol àmbit i clar si és el futbol i una persona com tu que tens aquest impacte, *pues* molt millor, però és una cosa que jo crec que a qualsevol feina. I és això, és educar te a conèixer-te a tu mateix i a tenir consciència de tu mateix i obrir-te davant d'altres. I no sé a mi em a mi m'agrada molt (O.1).

1. **E: I la última alguna millora que implementaries en el programa?**

P: *Bueno,* les dos coses que t'he dit, és a dir, una és que el temps no hagués fet que, no sé, que la gent no volgués compartir tant a lo millor, perquè hi havia molta càrrega en la sessió pel temps que hi havia. I jo crec que així podríem haver estat més temps compartint i fent xerrades que ho vam fer, eh? Lo que passa és que sempre, jo crec que tu també ho notaves, no que estàvem sempre amb el *petardo* al cul, sí, perquè tu, perquè tu ets molt respectuós del temps i jo crec que això és positiu, perquè fer lo contrari, no dir hòstia, hi ha més, hi ha més. Hi ha més i anar-te'n a l'hora i mitja, hora i 45. A vegades falta el respecte una mica els altres, però jo crec que això ho feies molt bé, però que també notaves que el temps ens ha faltat. I l'altre, lo de les meditacions amb en grup, saps?

**E: Comentar més no fer ne més. Bueno, això està una mica en les vostres mans, no Pere? Dins del Departament de Metodologia, no sé... com ho voleu valorar?**

P: *Bueno,* clar, és a dir, jo per mi és una cosa que quan la va publicar l'Isaac, jo estava interessat en fer-la, li vaig dir a l'Isaac sí hòstia, *pues* això estaria bé, no sé quants, perquè quantes sessions sou matí i tarda?

**E: De què? De què?**

P: De quantes esteu fent? És a dir, quants inscrits hi ha hagut en total?

**E: 71, 70.**

P: Hòstia, està molt bé.

**E: Ja està molt bé. Està molt bé. Sí, sí.**

P: De puta mare tio, jo clar, nosaltres érem 10 puta mare. Eh, però que no sabia quantes classes hi havia més i quanta gent hi havia més. Així que em sembla de puta mare, jo ho veig tio, que si es pot fer com incorporat dintre del club i això em sembla brutal, sembla brutal, perquè jo crec que la gent, encara que participi més o menys, està escoltant. A més ets un tio que ets eloqüent, saps? Llavors, no sé, jo et dic que *ojalá* fos un un programa integrat a com a salut laboral. No és a dir, igual que tenies la dels idiomes.

**E: Ja ja...**

P: Ho veig molt positiu perquè dintre del club estàs educant, estàs donant un espai de formació, l'estan adaptant a diferents hores i això em sembla top.

**E: Vale Pere, merci. Anem parlant, vale?**

P: Vale, que vagi, vagi bé. Una abraçada.

**E: Una abraçada. Adéu.**

**ENTREVISTA 18. XAVI (Analista, 28 años) – Código P.18**

**Realizada *online* el día 2 de junio de 2021**

**E: Xavi, tu estàs en concret al departament de *scouting* del club?**

P: Sí sí, ara sí, des de esta temporada passada.

**E: I abans on estaves?**

P: A anàlisis.

**E: Ah, vale vale, correcte correcte.**

P: Sí sí, estava en el departament d'anàlisis des de 2015 i aquesta temporada passada vaig passar a *scouting*.

**E: Molt bé, enhorabona, anar creixent, no?**

P: Merci, i tant i tant.

1. **E: La primera pregunta és com diries que el programa t'ha impactat, si és que ho ha fet alguna manera?**

P: Si, eh, *bueno*... a mi primer tot donar-te la enhorabona pel programa, m'ha semblat *super* profitós... i la veritat que ha estat molt bé, a mi m'ha agradat molt (O.1). Eh, jo vaig començar una mica a meditar este any perquè he tingut uns moments, alguns moments de *baixon*, una mica i tal i vaig començar a indagar una mica en lo tema de meditació i llavors casi que va coincidir con que va arribar al *mail* que començava a fer el programa este i, hòstia, em va anar molt bé per agafar una mica més de hàbits i tot això, i la veritat que a mi m'ha anat molt bé.

**E: i a nivell professional t’ha impactat d'alguna manera?**

P: Bé, a nivell professional... lo que vaig utilitzar més, és l’exercici aquell de concentració (PE.3) una mica no, del 40 minuts més el descans i estes coses si que t'ajuden crec que a agafar una mica l’hàbit, no? D’intentar concentrar-te en la feina, no distreure'm, no? lo que comentaves, en el mòbil, en no sé que ja estàs centrat únicament en allò que jo crec que et fa, te faci una mica més productiu clar.

**E: Molt bé.**

P: En este sentit, doncs sí.

1. **E: llavors la segona pregunta una mica relacionada es si has modificat o has incorporat algú comportament o alguna estratègia tant a la vida personal com professional**

P: Eh... buf, estratègia no, lo que sí que intentaré incorporar a la mesura que pugui és anar fent setmanalment diferents tipus de meditacions, pel temps que tinc al final no disposo de 20, 25, 30 minuts seguits per poder fer-ho, a nivell diari segurament no podré aconseguir, però com a mínim setmanalment intentar fer unes quantes meditacions... De les pràctiques estes, també que feien, hòstia, de notar el menjar, notar com te dutxes, el aigua calenta (PE.2), sóc més conscient de totes estes coses també, no? Que no sé si al final són estratègies, però sí que, *bueno*, ho recordes intentar fer tot això més... més conscient el dia a dia (AG.4).

1. **E: Molt bé. Qué diries que ha sigut el més beneficiós per tu del programa?**

P: Lo més beneficiós... eh, *bueno*, los coneixements una mica teòrics (CT.3) que has donat, que has anat explicant que a mi sempre m'agrada conèixer noves coses i d'on, surten, que beneficis tenen etcètera, etcètera, i sobretot, jo crec que el que més beneficiós m'ha... O sigui el que més m'ha agradat del programa final és la continuïtat que et fa tindre (EO.1), no? Un programa de cada setmana, doncs la sessió, tot i que jo no la podia fer en directe, els exercicis que anaves recomanant setmanalment, doncs tot això te fa... et fa créixer, no? El tindré unes obligacions entre cometes de cada setmana la classe i cada setmana exercicis nous (PE) i tal ostia això...

1. **E: Super, perfecte. Eh, quins han sigut alguns dels reptes i dificultats per completar el programa, si és que les ha hagut?**

P: Sí... eh... T'ho vaig ficar al diari, los de meditació sí que los vaig anar fent bastant menos alguna setmana sobre tot al final que em vaig quedar allà una mica penjat i no vaig poder, no ho vaig fer tant, però sí que ho vaig seguir molt i el què més m'ha costat pel que sigui, temps, se't passa la setmana volant i ja no fas tant estos temes, però com em vas passar els vídeos vull tornar a revisar quan pugui, ara que són vacances d'estiu i tal tots estos temes que eren més de... de sensorial, no? Em semblava, d'exploració del cos i d'estes coses no els vaig arribar, fer m'agradaria fer-les.

**E: Sí, sí, l’exploració del cos,**

P: Sí, tot això al final no ho he fet no per dificultats si no per tens meu i gestió meva horaris no he trobat lo moment, lo que sí que m'ha costat més em costa és quan faig meditacions més llargues que, com a tothom, quan he provat algun dia de fer 20 minuts costa més de mantenir la concentració.

**E: Sí, és part de l'entrenament, vull dir, és normal.**

1. **En quina mesura ha coincidit al programa amb les teves expectatives?**

P: Jo no en tenia moltes o sigui no en sentit de que no esperava res de tu tot al contrari, vale? Si no anava en la intenció de hòstia *pues* a veure com va, llavors les expectatives... *bueno,* conèixer una mica més tot com funciona tot tema de meditació de *mindfulness* i tal, però no, no anava amb cap expectativa general, simplement, a aprendre a passar-ho bé i a prendre nous hàbits i tal.

1. **E: Fantàstic. Què destacaries positivament del curs? D'alguna manera, com lo millor?**

P: Lo millor... Lo millor que t'he comentat. Jo crec que... la rutina de que ens donessis uns exercicis (PE) i noves motivacions i noves coses per fer perquè això ja et marca una línia que fer, una línia de treball, diguem, no? i et fa seguir... i després tot i que no he pogut està molt presencial, però tindre la classe setmanal si que et dona un poc este punt mes de continuïtat (EO.1) La força des del programa, no? El tindre’l a aquella hora, la meditació conjunta, tot això, jo crec que això és una fortalesa important

1. **E: Vale, genial. Vinga les dos últimes ja, com valores aquest tipus de programa pel teu rol professional?**

P: Eh... *Bueno,* bé (O.1), tot lo que t'ajudi, jo crec, a estar més centrat, més concentrat... Com ho diria... A relaxar-te, a ajudar-te a concentrar, doncs, penso que és molt positiu sobre tot el tema este de concentració és el que mes aplico a la feina (PE.3), si que és veritat que m'ho prenia més per aplicar a nivell personal, no? A la meva vida personal, però.... Que els dies que vas més carregat emocionalment, relaxar-te (AG.4) tot això. A nivell de feina ja t'he comentat també és útil sobre tot ho últim que t’he comentat del mòbil, per exemple, que et pot ajudar concentrar-te, centrar-te només en una cosa de feina.

**E: La concentració, no?**

P: Sí.

1. **E: vale... implementaries alguna millora en el programa, si és que ho faries?**

P: Eh, jo *bueno*... No, m’ha agradat molt (O.1), l’única millora pot ser que he estat pensant, potser allargar-lo una miqueta més (EO.1), per terminar d'agafar l’hàbit i si la gent tingués disponible *pues* fins i tot si es pogués fer dos classes setmanals, però crec que és lo que et deia, això t'ajuda a ser.. Al tindre unes hores a la setmana i uns exercicis que anaves marcant i tal, t'ajuda això, a seguir el curs, a centrar-te més en això, seguir els hàbits, les rutines tal tal tal i, hòstia, jo crec que simplement no per millorar si no per ajudar a la gent a seguir la rutina i a... a interioritzar lo més

**E: Eh... La meva proposta és la mateixa, o sigui, mantenir la classe dels dilluns**

P: Si...

**E: i una meditació grupal de mitja hora per exemple els dijous o els divendres llavors tu tens com no, t'assegures una mica un *anclatge*.**

P: Està molt bé, sí sí...

1. **E: Vale Xavi, algú comentari final? Algo que vulguis comentar que no t'hagi preguntat?**

P: No no no no...

**E: Perfecte, molt bé**

P: *Venga*, una abraçada

**E: Una abraçada Xavi, adéu.**

**ENTREVISTA 19. ÒSCAR (Coordinador, 29 años) – Código P.19**

**Realizada *online* el día 2 de junio de 2021**

1. **E: Com diries que el programa t’ha impactat, si és que ho ha fet alguna manera?**

P: Bé... no ho havia provat mai, la veritat és que no havia tingut aquesta experiència, no? De la meditació, i la veritat és que el *feedback* positiu, eh... positiu. Trobes un moment de... un moment d’intentar, *pues*, concentrar-te amb tu mateix amb aquesta respiració en el saber fluir i tot això i bé, bé bé, bastant positiu.

**E: Molt bé, a nivell professional destacaries alguna cosa?**

P: Jo crec que no, fins a cert punt perquè no he agafat tant l’hàbit... Que no he arribat tant com per trobar aquell punt de connexió, com fer l'hàbit ja meu i trobar aquest punt de serenor o traslladat a la feina, però jo crec que en un llarg termini o mig llarg termini si que em podia afectar (O.2) però no he trobat gaires canvis, sí que en algun moment de serenor, doncs d’ansietat de feina... saps el que vull dir?

**E: Sí, perfectament.**

P: D'estrès, situació de dir: buf, calma... baixa, baixa a la terra que has de respirar i has de tal. Això sí que ho he notat una mica però no en grans moments no. M'entens el que vull dir?

**E: Perfectament, sí sí.**

1. **E: Has modificat o has incorporat algun comportament o alguna estratègia de les que s'han parlat al curs tant a nivell personal com** **a nivell laboral?**

P: Més a nivell personal del dia a dia, no? El que vas comentar el tema de conduir d’intentar estar sabent que estàs conduint, el tema de pujar escales (PE.2), sàpigues que estàs pujant escales i... i notar els músculs, la contracció i tal, ser més conscient del moment no d'aquest moviments més automàtics o de que estem al pilot automàtic, això sí que ho he... o sigui en aquest moment doncs ser més conscient d'això, o d’estar a la conducció el sàpigues com estic se col·locat, la posició i tot això, això sí que intentat hi alguns moments en venia la reflexió.

1. **E: Què diries que ha sigut el més beneficiós per tu?**

P: La respiració, el moment de tenir de calma i baixar aquestes pulsacions i de sentir i de relaxar-se, el moment que feia les meditacions de sentir després com una pau al finalitzar d'estar tranquil amb mi mateix, d’estar relaxat (PE.1), de sobretot potser aquests problemes que durant el dia o durant un moment li donem moltes i estem molt capficats en allò de saber evadir-se d’allò i portar-nos més en el en aquesta relaxació o en com enfocar-lo de una l'altra manera jo crec que es la que mes m’ha ajudat (AG.4).

**E: Brutal.**

P: Aquesta respiració de baixar aquestes pulsacions i de intentar veure tot amb un altre prisma... Això m'ha ajudat molt, és cert (O.1).

1. **E: Molt bé, algun repte o dificultat per completar el programa?**

P: M'hagués agradat fer més fer més meditacions (PE.1) això és veritat que no he fet els 7 dies encara que deies de fer encara que fèieu 3 minuts intenteu fer-ho cada dia, és lo únic que com a repte o dificultat he vist per una cosa o altra o que tenia que sortir fora o estar a reunions i tornar i dinàvem fora i tot això o qualsevol cosa i que no he pogut seguir. Aquesta és la única dificultat, la única.

1. **E: A nivell de expectatives, si és que les tenies, el curs ha coincidit amb les teves expectatives?**

P: Sí sí, i tant, i tant o sigui no tenia expectatives molt altes perquè era un tema totalment nou per mi, però no esperava sentir el que el que he sentit, no esperava ja saps e no esperava tenir tenir aquesta percepció o aquestes vivències després de meditar (O.1) m'entens? Era algo nou per mi, no tenia expectatives molt altes, però bé, la veritat és que bé.

1. **E: Molt bé. Què destacaries positivament del curs, del programa?**

P: Eh... positivament... És que a veure és tot un conjunt, el què t'ha dit de les sensacions després de fer el programa, doncs, el que jo he sentit és positiu per mi, i d'altres persones trobaven una cosa més positiva, no? Perè, sobretot, lo de les emocions em va agradar molt la tristesa, la ira, lo de la angoixa, tot això que anem parlant en la diapositiva, jo crec que és positiu saber reconèixer aquestes emocions i reconèixer com et sents, saber reconèixer en quin moment estàs tu mateix i com t'estàs sentint. Jo crec que és lo més positiu del programa o sigui al el connectar més amb tu mateix i saber com estic, com em trobo (AG) i buscar aquest benefici, jo crec que es lo més positiu

**E: Molt bé, molt bé, m'encanta.**

1. **Ja les dues últimes, com valores aquest programa, aquest tipus de programa pel teu rol professional?**

P: Eh... ho valoro positivament (O.1) perquè, *bueno*, com saps moltes vegades hem de fer molt *multitasking,* que portem de una reunió, portem una trucada, hem de fer sessions i coses que ens portem a altres coses... i saltem molt de llocs en llocs i hem de trobar moments per seure de peus a terra i de a veure calmem-nos i anem a organitzar-nos bé, potser fer una organització més acurada no del que del que toca, trobar una mica més de lògica en el dia a dia o amb l'agenda que tenim programada i de trobar un moment per tots i de no caure aquest estrès o en aquest moment d'ansietat... no? (AG.4) A vegades nosaltres ens passa i més i ho saps i en el club (riuen) és com totes, Aleix estàs disponible per ahir a les cinc i és com: si si estic disponible si si si (riuen), si no és com buah eh... no ho estic fent bé i clar, a vegades, et tires molt de pes a sobre de dir i estàs sempre *ready* he d’estar sempre per tal i has d’estar sempre preparat al 100 per 100 i a vegades pues bueno has de has de frenar i tot el que sigui organització pròpia ho hem de gestionar bé perquè sinó si ja les externes que ens venen precedides de coses com aquesta i les que podem tenir de presa de decisió tampoc les organitzem bé... podem estar fotuts (riuen).

**E: Molt bé, molt bé, molt encertat.**

1. **Quines millores implementaries en el programa, si és que ho faries?**

P: Pfff... a veure és veritat que jo l'única millora seria a nivell més presencial (EO.1), jo crec que que a nivell presencial hagués tingut més aquest ancoratge, no? Aquesta relació més entre nosaltres, més que sentim aquestes vivències, d’unir-nos més, de no estar només darrera la pantalla on potser no dic res... si no més fluïdesa a l'hora d'entre vosaltres de com ens sentim de com estem visquent el programa i potser trobar més punts en comú és potser que trobaria com a... i sí que és veritat potser a nivell presencial enlloc d’un cop a la setmana potser dos i trobar una mica més del lligam entre sessió i sessió potser no sé no sé, també entenc que ets de fora i les posicions que s'estableix que *bueno pues* que estem que és lo que dius que tenim moltes coses a l'hora sabia que no *pues* que no volies sumar moltes hores no al programa i tenir que estiguessin pendents de d'aquesta hora i mitja dos dies a la setmana i tal, però jo crec que dos dies a la setmana (EO.1) potser pot estar més lligat al programa jo feia dilluns i dijous pues ja no faig fins dilluns que ve.

**E: Clar**

P: I era una setmana seguida, potser fer una altra *pues* dijous i tenim que hi hagi aquests tres dies i fas com una mica més de continuïtat i et sents més pertinent en el que estàs fent potser, no sé...

**E: Clar, exacte, aquesta és la idea No, jo sé que els dijous us enviava un *mail* d'aquests de recordatori, la idea seria completar ho amb una meditació el dijous, saps?**

P: Correcte.

**E: Llavors la meditació d’un quart d'hora 20 minuts si vols 10 minuts per parlar qualsevol cosa.**

P: Correcte.

1. **E: Vale Òscar res, si vols comentar si vols afegir tu alguna cosa?**

P: No no no, res res, que moltes gràcies, gràcies pel programa, Aleix, que hi ha una feina darrera que és brutal de recerca, de llibres, de lectura i de seguir formant que t'ho agraeixo, t'ho agraïm tots i moltes gràcies pel programa (EO.1).

**E**: Gràcies a tu, Òscar, que vagi molt bé!

P: Adéu, adéu, adéu.

**ENTREVISTA 20. MARTÍ (Entrenador, 35 años) – Código P.20**

**Realizada *online* el día 7 de junio de 2021**

**E: Martí, entrenador del futbol formatiu, has participat en el programa de *Mindfulness* per Entrenadors, la idea d’aquesta entrevista és fer una mica un anàlisi qualitatiu i saber la teva opinió, vale, l’únic que es demana és sinceritat, no hi ha respostes correctes ni incorrectes simplement el que ha sigut la teva experiència.**

1. **Llavors la primera pregunta és com diries que el programa t’ha impactat, si és que ho ha fet d’alguna manera?**

P: Home doncs la veritat que el impacte, el impacte...ha sigut significant sobre tot pel de bueno...de tenir aquesta consciència en moments en els quals et deixes portar i allò que parlaven durant el programa i estàs en pilot automàtic doncs, doncs trobar aquests moments de de... de pausa, de reconnexió amb el moment present ja no a nivell de la feina sinó a nivell personal també. Penso que ha sigut molt profitós (O.1).

1. **E: Brutal, eh... has incorporat o has modificat algú comportament, alguna estratègia, tant a la teva vida personal com a nivell professional?**

P: *Bueno,* a la meva vida personal he intentat incorporar les meditacions intento fer entre 5 i 10 minuts de meditacions 3 vegades per setmana, de moment.

**E: Molt bé.**

P: I a nivell professional sí que ho intento... intento sobre tot durant les sessions de entrenament que és un context una miqueta menys exigent i intento doncs sobre tot a la sessió amb el company, amb el Mario quan ell dissenya la sessió s’encarrega de portar també la iniciativa durant la sessió, en aquest moment es quan jo puc, també, doncs concentrar-me una miqueta més en coses que a vegades se’t passen una miqueta desapercebudes per estar més més... més pendent de quines són les reaccions dels jugadors o de com estan sentint els conceptes que proposem (PE.3). Sí, algo he anat practicant, a alguns dels partits que vam tenir que era un context molt exigent contra el segon classificat, contra el Vilafranca, recordo que normalment abans de ...just abans de l’activació i final, abans del partit, normalment tinc un parell de minuts en els que parlo amb els jugadors després de la xerrada del Mario intento reforçar algú missatge i recordo que aquell partit les hi vaig demanar que com que era un partit molt exigent vaig demanar als jugadors que es paressin un moment, fessin una respiració profund ai (eee)... i que estiguessin concentrats no mes en el partit i en intentar fer tot allò que portaven practicant durant tants mesos no. I... bueno crec que va ser una bona practica perquè al final el jugadors mai saben no, hi ha tants...tants factors que determinen el rendiment dels jugadors que al final saps fins a quin punt això va tenir un impacte en el equip, però jo penso que... que aquestes... que aquestes pràctiques a nivell puntual, en moments així d’una miqueta d’estrès i exigents son molt positives (respira)

**E: aaa... vau guanyar?**

P: Vam guanyar, vam guanyar 3 a 2

**E: Van sortir, van rendir bé, ¿no?**

P: Sí, sí sí, el partit va anar bé, van sortir *enxufats* i era ja te dic un context complicat contra el segon classificat que havia guanyat tots els partits, el Vilafranca, jugadors de segon any, a nivell condicional estaven per sobre dels nostres, però *bueno*, van estar molt bé la veritat.

**E: Enhorabona. Mira ahir sentia una frase que deia “si saps que no resta i si tenim dubtes que... que no sumarà, no sabem si sumarà o no, però sé que segur que no restarà”**

P: *Adelante*.

**E: Fotem-li canya, saps? tampoc sé si és la realitat d’aquestes pràctiques, però això, al final m’alegro de que es vagi incorporant, jo crec que està molt bé, també cada jugador reacciona diferent al *feedback* que tinguin, però ara estem en una... jo crec que tu estàs amb una edat molt maca que... que no tenen massa soroll al cap, que no diran “què em diu aquest tio”, ara el Martí i el Mario son una mica referents no, si el Martí em diu que jo em calmi i respiri “*yo voy contigo a muerte*”, llavors crec que ara és un molt bon moment per anar incorporant aquestes estratègies.**

P: Sí sí, totalment, són esponges.

1. **E: Sí sí... què diries que ha sigut lo més beneficiós per tu del curs?**

P: Lo més beneficiós... *bueno*, jo et diria sobre tot a nivell professional, a nivell personal intentava sobre tot aquestes pràctiques de gaudir de moments puntuals, fa molt temps que ho faig i com tu saps vaig estar a la Índia

**E : que...**

P: Vaig estar amb molts companys que... que em parlaven d’aquests... d’aquests temes i des de ja fa molt temps intento gaudir de moments puntuals (PE.2) com a... aquestes pràctiques que vam fer de la dutxa, del menjar... sobre tot del menjar que, *bueno*, més fàcil perquè m’encanta o sigui intento traslladar-lo al meu dia a dia a nivell personal i... i sobre tot a nivell professional com et comentava l’impacte... l’impacte ha sigut... ha sigut gran i penso que positiu, tant a les pràctiques com als partits intento tenir moments de concentració, intento aïllar-me un pèl de, Bueno, de tot el soroll i tots els altres estímuls que hi ha durant... durant els partits i estar un pel mes concentrats per poder prendre millors decisions (PE.3).

1. **E: Brutal, molt bé Martí, algún repte de dificultat per completar el programa, si és que els has tingut?**

P: Repte..., *bueno*, repte pel meu context, trobar un espai (PE.1) on tinc un pèl més de tranquil·litat, perquè estic compartint pis amb un parell d’amics i, *bueno*, al final hi ha una miqueta sempre de de...soroll i moviment aquí a la casa on estic, però intento, vaig intentar trobar el espai adequat i aquest va ser pot ser el repte més gran.

1. **E: Perfecte. Expectatives, en quina mesura ha coincidit la formació amb les teves expectatives, si és que les tenies?**

P: Sí, *bueno*, com et comento tenia alguna petita idea del *mindfulness*, també a Estats Units, és una cosa que estan incorporant bastant, ho havia sentit a la Índia, després als Estats Units, també és una pràctica que s’està posant bastant de moda, per tant, la coneixia, tenia certes nocions, però *bueno*, a nivell d’expectatives, les ha superat moltíssim, les meves expectatives de continguts, a nivell de qualitat i sobre tot a nivell de tota la referències i, a més que s’ha anat convertint que... que penso que és un document que... és una informació que tindrem sempre amb nosaltres, que es pot compartir i... totalment ha superat moltíssim les expectatives (O.1).

1. **E: Me n’alegro molt, Martí. Va vinga 3 últimes: què destacaries positivament del curs?**

P: *Bueno,* positivament sobre tot... el fet de poder compartir amb companys amb qui normalment no estàs en un context de com el que tu platejaves o proposaves, estàs en un context més de tranquil·litat, de no jutjar, sobre tot perquè al final estem en un context competitiu, nosaltres els entrenadors que, *bueno*, trobar aquest espai en el que estàs amb altres companys que comparteixen professió i club (eee) i en un entorn molt més relaxat on pots parlar d’algunes coses, les pràctiques que vam fer que vam poder exposar alguns conflictes que havíem tingut en el passat amb algú company i el fet, bueno, d’escoltar de tenir aquest espai i penso que, que *bueno*, és el que més destacaria sobre tot (O.2).

**E: Molt bé, tio, saps que és que és algo que moltíssima gent m’està dient el mateix tio, que aquest espai de de... no sé com segur, de poder compartir, de veure a la gent explicar-se, d’obrir-se, de dir òstres, que sempre ens centrem en aquest context competitiu que sembla que tots som màquines i tal, però quan ens obrim una mica i m’agrada molt.**

P: Sí sí, totalment, brutal.

1. **E: I com valores aquest programa per el teu rol professional?**

P: Pel meu rol professional com ja he comentat molt positivament, sobre tot pel fet de trobar aquets moments de de... de deixar-te una miqueta del pilot automàtic i trobar aquest moments de pausa, de reconnexió, de realment ser capaç de... al final el rol del entrenador es tant complicat i tant excitant a la vegada que has de gestionar tants estímuls i tants factors i tantes coses que trobar aquest moment és vital o sigui crec que pel món professional, i sobre tot pel món on ens movem nosaltres del de l’entrenador de futbol és crucial (O.1)

1. **E: Eh... vinga ara si les dos últimes, quines millores implementaries en el programa, si és que ho faries?**

P: *Bueno*, millores eh... a veure jo penso que ha estat tot fantàstic, sincerament millores hauria de pensar-ho millor... potser donar-li un pel més de continuïtat (EO.1), crec que ho vaig escriure també a la entrevista, fer un pel més de seguiment com a mínim una vegada al mes un cop ja has acabat el curs com mínim seguir en contacte i aquest i aquest grup tan maco que s’ha format doncs seguir com mínim donant-li una miqueta de continuïtat, penso que estaria bé. Durant el curs en si, crec que ja et dic, les pràctiques van estar fantàstiques, l’horari perfecte, la durada crec que tu sobre tot anaves un pel de bòlid sobre tot algunes vegades en el contingut, si això alguns minuts mes de duració de cada sessió no t’agüessin vingut malament sobre tot a tu perquè tocàvem molts temes molt interesants i anàvem un pel un pel de bòlid amb tot el contingut que havia, però penso que penso que molt bé la veritat, és que poques coses a millorar, el fet aquest de donar-li un pel més de continuïtat pot ser.

1. **E: Perfecte Martí. *Pues* ja esta, algun comentari que vulguis afegir, algo que no t´hagi preguntat?**

P: Doncs no, penso que hem parlat de tot, o sigui, que res encantat d’haver format part del programa i, ja et dic, *bueno*, a veure si tenim la possibilitat sobre tot allà a Estats Units que segur que en gaudiran els entrenadors i de més, i res, esperem que es doni continuïtat, no se si través teu des dels Estats Units o a través d’alguns dels companys que formaven el grup de... que va proposar el programa de *mindfulness,* doncs seria interessant.

**E: Ho intentarem, *estamos en ello.***

P: Vale

**E: Merci Martí (riu)**

P: No home no, gràcies a tu, Aleix, que vagi molt bé.

**E: Una abraçada, que vagi bé! Adéu**

**ENTREVISTA 21. ESTEVE (Entrenador, 31 años) – Código P.21**

**Realizada *online* el día 7 de junio de 2021**

**E: Vale vale Esteve, entrenador del futbol formatiu que has participat en el programa i has accedit de forma voluntària a aquesta entrevista qualitativa i la idea és bàsicament preguntar-te sobre la teva experiència amb el programa. No hi ha respostes bones ni dolentes, i simplement sigues sincer per poder, nosaltres també doncs, avaluar amb calma, vale?**

1. **E: La primera pregunta seria com diries que el programa t’ha impactat, si és que ho ha fet d'alguna manera?**

P: Com vaig dir al l'inici, a mi em va cridar molt l'atenció quan vas presentar la idea més general a pinzellades al Barça Coach Academy del programa i, la veritat, és que m’he omplert de noves... no noves idees, però maneres d'enfocar aquesta.

**E: Pots concretar?**

Ara en parlàvem, també, de si no està a les meves mans, sempre el que està a les teves mans i pensava ostres, això m'interessa perquè fa temps que estic intentant donar més sentit a aquesta manera d'entendre, de comprendre la vida. I llavors em va cridar. I a nivell de continguts, em va agradar molt tant a nivell teòric, sobretot a els temes que han tractat com els ajuts, com els organitzat i per exemple, el tema de les emocions (EO.2) en va és una cosa que no hi havia pensat i vaig em va em va generar bastant interès, que és el tema d'on venen les emocions del passat, del la por al futur, la tristesa del passat, l'alegria del present i no hi havia reflexionat mai. Amb el tema que has anat parlant també de d'aquesta concentració plena en moment. Tots tenim un impacte i com tenir impacte. Sí que és veritat que el tema de la meditació (EO.2) que el li has donat un pes rellevant també. És el que personalment, eh? O sigui, no dic que no serveixi, però a mi m'ha costat, no m'ha acabat, no m'ha acabat de convèncer, però perquè bàsicament suposo perquè veníem les idees preestablertes que també n'hem parlat. I era més que jo, pensava que era com atreure o la meva interès eren com atreure la concentració plena o l'atenció plena del jugador en envers no jo mateix com a treure la seva concentració i com buscar la seva pròpia. Crec que és més per trobar-te a tu. La part pràctica, tot i que ja has vist que jo la part pràctica he anat afegint coses en direcció als partits i els entrenaments que anant més cap aquí que com analitzar la aquesta pel jugador com està concentrat que està assimilant dels estímuls amb i amb i per compte també. Això també m'ha anat bé, no meditació, però jo abstreure'm, pues mirar des d'un altre de vista més fred per tenir un millor anàlisi del que està succeint en el moment (PE.3).

**E: Molt bé...**

P: Me n'he anat molt, no?

**E: No, no... perfecte les coses que has contestat que potser estan a altres preguntes, jo ja ho organitzo.**

1. **E: Has modificat o has incorporat algun comportament, alguna estratègia en el teu dia a dia, tant personal com professional?**

P: Sí, el fet de prendre atenció de coses que abans no li prenia, sí que ho he intentat aplicar El problema és que no sé quina continuïtat tindrà, perquè m'havia de... o sigui, havia de pensar-hi en aplicar-ho. Llavors no sé si tindrà continuïtat. Potser sí que m'he fixat que em fixo sempre he sigut de fixar-me molt en detalls, però en fixar més amb detalls de persones, els caràcters i actituds de la gent amb detalls de d'aquesta expressió nova (RI.3). Però, en canvi, no em fixava en altres detalls. Posa... al jardí, he d'estar al jardí. A vegades sí que em miro coses i em pregunto: per què passa? Per què el vent comença moure les fulles d'aquesta manera... i sonarà una mica a filòsof això, però, sí que abans, segurament lo que era inert o no hi havia personalitat o una part psicològica al voltant, no m'hi fixava tant, anava més a la psicologia de les persones i a aquesta part més natural, més inerta de l’element, *pues* segurament no, no hi prenia tanta atenció i ara sí que intento buscar-hi detalls, també, en aquest sentit.

**E: Molt bé. I a nivell professional, destacaries alguna estratègia o comportament?**

P: És el que et deia les he intentat de portar cap a lo que a mi em cridava l'atenció que és, per exemple lo que et vaig comentar. També no sé si eres el Coach Program, el fet de que l'han les emocions de l'entrenador quan es converteix en un jugador més. El fet d'extreure per buscar aquesta concentració plena, pròpia i alhora buscar què necessita el jugador des d'una vessant més... més entrenador-observador. Què necessita el jugador per aconseguir aquesta atenció plena que li fa no tenir els nervis i pressió, centrar-se en la pilota, que és un detall que sí que ha aplicat. *Pues* sí, tant, tant a nivell d'actitud com a nivell de de transmetre el jugador o buscar contingut per fer-li arribar el jugador. Sí que sí que he aplicat certs continguts (PE.3). Ja et dic derivant una mica cap al cap a on volia anar jo que era més a la concentració dels jugadors que la meva pròpia, però sí.

**E: Perfecte. Aquesta és la idea, eh? Transmetre el que tu reps en base al que tu creus en aquesta línia, jo crec.**

1. **E: Què ha sigut el més beneficiós per tu del curs?**

P: Bueno, crec que una mica aquest gir de que... ja et dic que portava temps i, per tant, ja ho havíem parlat amb el Pau a Nova York, aquest centre en el que depèn de tu *pues* m'ha ajudat a potser tenia una idea molt general del que era centar-me en el que depèn de mi i ara *pues* detalls de *pues* lo que parlàvem de ostres l'emoció de la por no l’havia pensat mai que estava relacionada amb el futur, la gent l'analitzada com a pobre i com a emoció i prou. I no li busca aquest origen. O la tristesa o l'alegria, en aquest cas que parlem també del present i del passat, i sí que això permet donar-li una volta més a aquesta... potser una idea borrosa que tenia d'aquesta centrar-te en el que depèn de tu (AG) i, ara, puc anar més a buscar els orígens, els perquès més eficientment.

1. **E: Molt bé, molt bé. Algun repte o dificultat que t'ha sorgit per completar el programa? Posaré aquí, per exemple, lo de la meditació que has comentat, vale?**

P: He sigut una persona molt distreta i em costa en aquest sentit. O sigui, sóc molt reflexiva en aquest buscar la concentració plena i ens centrar-te en el que t'has de centrar, però necessito molts, molts estímuls per poder arribar allà i en canvi la meditació em falta. Em falta algo? Suposo que a mi em va massa el cap o li dono massa voltes a les coses, llavors, no he trobat el que segurament... si fos capaç d'assentar-me 100% li trobaria eh, però és com seria tot el contrari del que busco per un per concentrar-me que és tenir molts estímuls. Teníem molts nous reptes, però reptes del minut a minut que en generin aquesta concentració, no? busco la calma la busco en el en el caos, com si diguéssim, llavors, potser per això. I aquesta idea preestablerta que segurament és un error, però sí que m'ha costat del tema de meditació (PE.1) i m'ha costat trobar-li el propi interès. En aquest apartat.

1. **E: Vale no perfecte. En quina mesura la formació el programa coincidit amb les teves expectatives?**

P: M'ha sorprès, però m'ha sorprès en positiu i m'ha aportat (O.1). Per tant, ja és positiu tot lo que suma i et sorprèn, per a mi, ja li dona sentit al intentar anar evolucionant, no? La valoració general per a mi, molt positiva.

1. **E: No, vale, vinga les 3 últimes: què destacaries positivament del curs, com si diguéssim lo millor?**

P: Els temes m'han agradat i com estan organitzats, sobretot de contingut i Òmnium, parlaríem més del contingut. L'estructuració del marc teòric m'ha agradat molt (EO.1). I potser he trobat en jo adaptar una mica la part tàctica i portar-la a cap a casa, també el buscant l'essència en aquest marc teòric amb el portava cap a la pràctica per generar-me aquest interès. De de per lo que dèiem, *pues,* segurament a mi amb la meditació em costa trobar aquesta concentració. Però traslladar-ho al partit i intentar analitzar molt més i amb més coneixença de que analitzo amb molts estímuls m'ha permès centrar-me realment el que m'havia de centrar (PE.3).

1. **E: Com valores aquest programa pel seu rol professional?**

P: Tot lo que s'ha gestió de caràcter emocions pròpies, sobretot dels que t'envolten, de l'anàlisi d'estímuls, pues a mi tot això m'interessa molt, per tant super positiu (O.1). M'agradaria potser veure una segona versió (EO.4) teva quan si, si realment hi és o te la planteges de com partir d'aquí d'aquestes idees primer principals com enfocar-les més, perquè també et dic que no sabia cap on ho portaria jo. Amb la desconeixença, em genera curiositat cap a on aniria ara quines serien el següent pas, fer la fase dos i cap a on s'expandiria, saps?

**E: La fase dos, no?**

P: II, però, a nivell de gestió de persones, gestió de caràcter, gestió de provocarem de de formes de ser de d'aquest. Aquest prioritzem que prioritzem, pues tot això m'interessa quasi més que el marc teòric del futbol, però al final són gestors de grups i de persones, per tant, jo tot aquest camp del *coaching* que potser seria un derivat del *coaching*, no també? O una eina més pel *coaching*.

**E: Això sí, sí, sí. Una eina més podria ser així, tant el psicòleg esportiu com pel coach.**

P: Sí, pues tot això em genera molt d'interès. Per tant, crec que professionalment, creixent en aquest àmbit, és importantíssim.

1. **E: Val, eh... Implementaries alguna millora en el programa?**

P: Uf és que potser és que em costa molt dir millora o si millores perquè no em considero capaç de generar la base aquesta que diem de a partir d'on expandir-se, per tant, em sembla una mica surrealista que jo pugui dir millores, però potser aplicar-les més per dir algo, per sumar i igual et serveix amb el que el que et comentava que portar més la part tàctica a prop, acostar-la als interessos de cadascú, *pues* potser més trobar la calma pròpia. L'altre potser trobar més el... no sé si m'estic explicant...

**E: Sí, perfectament, perfectament. Sí, sí, potser passar del menjar de la dutxa i de certes coses a coses més concretes. *Pues* sí.**

P: Ser més basades, ser més basades en l'interès per propi i no tant en rutines quotidianes, potser.

**E: Molt bé.**

P: O sigui sumatori, no, no, no per corregir res.

1. **E: Sumatori a la pràctica formal. I em sembla que ja està. Si vols afegir alguna cosa més, Esteve?**

P: No res no és que res agrair-te el temps dedicat i el coneixement compartit. Ha sigut molt productiu i agrair-ho perquè com aquell qui diu ho fas per *amor al arte*.

**E: Sí, sí.**

P: Això és un pas d'agrair en un món que al final sembla que tothom es vulgui guardar lo seu ben protegit per no enriquir els demés, perquè igual marxaven després i en un món on cada dia es veu més això, *pues* és d'agrair, el voler compartir el coneixement sense buscar res a canvi, o el fet de que sigui recíproc.

**E: Correcte bueno, jo interpreto que si hem de créixer, creixem tots junts i com més gent hi tingui interès pel tema, *pues* em faran millorar a mi mateix. I és una decisió des de l'amor, no des de la por, no? I si jo d'alguna manera de vegades em queixo de certes actituds, *pues* jo de ser el primer que, que aporta Esteve, moltíssimes gràcies.**
