# Supplementary material for: Impact of mindfulness on football coaches: A mixed design
Source: PLoS One. 2024 Oct 3;19(10):e0306824. doi: 10.1371/journal.pone.0306824 (PMC11449272; doi:10.1371/journal.pone.0306824)
Supplement: Attachment — Submitted filename: Response to Reviewers.docx [file pone.0306824.s003.docx]

**Response to Reviewers**

**Reviewer #1: The manuscript (MS) is generally well-written, and appropriate methods have been implemented in the design, ethics, intervention, and analyses.
I would like the authors to address:**

**(1) Why the wait list control group received the program just after the pre- and post- evaluation? This does not allow to observe possible differences in the two follow-ups…**

We are aware of the methodological limitations of this and it was forced by the Club who wanted to provide the program to as many staff as possible and as soon as possible. In order to get the whole study done, we had to agree on this situation.

**(2) In the whole MS, the authors use the expression “decrease in difficulties in emotion regulation”. This is, in my opinion, counterintuitive and I would generally refer to “improvement in emotion regulation”.**

Agreed and changed in the manuscript.

**Please, see my specific comments below:
Abstract
- “(MxE)” please specify what MxE is**

Done.

**- “A significant decrease in difficulties in emotion regulation” I would write “significant improvement in emotion regulation”**

Done.

**Introduction (No particular comments)**

**Method
Why the wait list control group received the program just after the pre- and post- evaluation? This does not allow to observe possible differences in the two follow-ups… Please, provide a reason for this.**

We are aware of the methodological limitations of this and it was forced by the Club who wanted to provide the program to as many staff as possible and as soon as possible. In order to get the whole study done, we had to agree on this situation.

**Lines 109-114, the authors state that 48.3% of participants meditated sometimes; and that the sample consisted of coaches (39.7%), technical directors (12.1%) and coordinator (34.5%). Were these proportions maintained in the experimental and in the control group?**

Yes, proportions were very similar in both groups.

**Line 132, at this point I presume MxE stands for Mindfulness for Enhancement? Please, specify at the beginning of the manuscript and in the abstract**

Done.

**Line 147. The table is very well-done and clarifies the whole MxE programme.**

**Lines 192-193. “translated the questionnaire”? Then the authors can mention the fact that the questionnaire was not previously validated in Spanish language in the Limitation section at Lines 441-447.**

Corrected.
 **Lines 195. “of THE Difficulties IN Emotion Regulation Scale”?**

Corrected.

**Lines 199-200. Check verb tenses, please.**
Corrected.

**Lines 201-202. Please, expand this part with the description of the practice-log.**

Expanded.

**Line 221. Standard Deviation (SD)?**

Corrected.

**Results:
Line 280. Table 3 should mention somewhere that these data were relating to the sole experimental group.**

Corrected.

**Lines 345-348. If this theme “was widely highlighted”, the authors could expand a bit more this paragraph.**

Expanded.

**Discussion:
Lines 365-366. I understand that the questionnaire was measuring “difficulties in emotion regulation” and that there was a decrease in these dimensions. However, it seems odd to read “a significant decrease in difficulties in emotional regulation”. I would invite the authors to consider writing “a significant improvement in emotion regulation” here and in the abstract. All fine in all the other parts I have read so far.**

Corrected.

**Line 376. Again “significant decrease in difficulties in emotional regulation”. Please, consider rewording this sentence.**
Corrected.

**Lines 383-385: “The transfer of mindfulness …” this sentence is a bit unclear to me, I would reword it as follows: “From the interviews, it also emerged that participants started to transfer what learned in during the mindfulness sessions to daily training, gaining deeper vision on players’ behaviours: “…”**

Corrected.

**Line 443-444. “A limitation was due to the fact that the scale did not receive previous psychometric validation. However, best practices for questionnaire translations were followed for adapting the scale for use within Spanish speakers.” Or something along these lines. Please, add a reference to support that the adopted procedure was a best practice for questionnaire translations.**
Corrected.

**Line 464. “on coaches, support staff and technical directors’ mindfulness ability and emotional stability”?.**

Corrected.
